# Solvation Free Energies for Aqueous and Nonaqueous Solutions Computed Using PM7 Atomic Charges

**DOI:** 10.1021/acs.jcim.1c00885

**Published:** 2021-09-15

**Authors:** Sergei F. Vyboishchikov, Alexander A. Voityuk

**Affiliations:** † Institut de Química Computacional i Catàlisi and Departament de Química, Universitat de Girona, Carrer Maria Aurèlia Capmany 69, 17003 Girona, Spain; ‡ Institució Catalana de Recerca i Estudis Avançats (ICREA), Passeig de Lluís Companys, 23, 08010 Barcelona, Spain

## Abstract

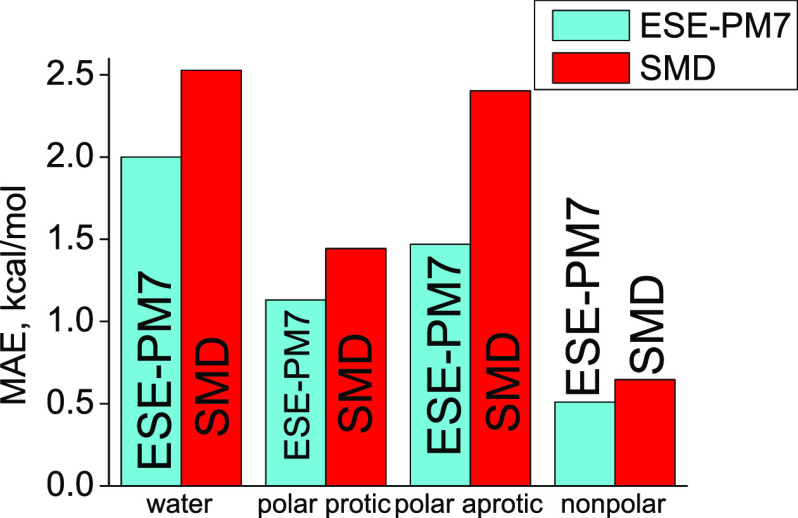

We describe a simple and accurate method, ESE-PM7, for calculating solvation free energies Δ*G*_solv_^°^ in aqueous and nonaqueous solutions. The method is based on a noniterative COSMO algorithm. Molecular geometries and atomic charges calculated using the semiempirical method PM7 are used to calculate Δ*G*_solv_^°^. The method has been tested on 92 different solvents and 988 solutes. The mean absolute errors (MAEs) in Δ*G*_solv_^°^ in aqueous solutions estimated by the ESE-PM7 approach are found to be 1.62 kcal/mol for 389 neutral solutes and 3.06 kcal/mol for 139 ions. The MAEs for neutral molecules in organic solvents are 0.97, 0.74, and 0.51 kcal/mol in polar protic, polar aprotic, and nonpolar solvents, respectively. The developed method can be employed to quickly screen Δ*G*_solv_^°^ values of extended molecular systems including pharmaceutical and biological molecules.

## Introduction

The solvation free energy Δ*G*_solv_^°^ plays an important role in computational chemistry, since it can make a significant contribution to the total free energy of chemical reactions in solution. Most practical calculations of Δ*G*_solv_^°^ are based on the continuum solvation (CS) model. Usually, the computed Δ*G*_solv_^°^ is represented by the sum of the electrostatic energy *E*_elst_ and the correction term Δ*G*_corr_^°^, which mainly describes nonelectrostatic effects:
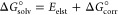
1

The existing CS methods differ in the treatment of both *E*_elst_ and Δ*G*_corr_^°^. The most popular CS approaches are the polarizable continuum model (PCM)^1−13^ and the generalized Born (GB) method,^14,15^ including SM*x*^16−20^ and SMVLE^21^ methods. The solvation methods in general^2^ and PCM methods in particular^1^ were reviewed in detail elsewhere.

The general idea of the PCM is that the solute placed in a cavity interacts with the solvent represented by a continuum with certain electrical properties. The polarization of the solvent by the solute is described by an electric charge distribution on the surface of the cavity. The charge distribution, in turn, is represented either by a continuous surface charge density σ(**r**) or by discrete induced charges {*q*_*i*_}. The electrostatic energy *E*_elst_ is calculated from the energy of the induced surface charge density σ(**r**) in the electrostatic potential of the molecule *V*(**r**):
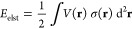
2where ∫d^2^**r** is a surface integral over the molecular cavity. The electrostatic potential *V*(**r**) is evaluated either from the electron density ρ(**r**) and nuclear charges {*Z*_*A*_} located in nuclear positions {**R**_*A*_}:
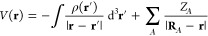
3or from atomic charges {*Q*_*A*_}
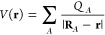
4

Although the first approach (eq 3) is in principle more accurate than the discrete-charge formulation (eq 4), it suffers from the so-called outlying-charge problem.^22,23^

The cavity surface is typically constructed as a superposition of overlapping atom-centered spheres,^24^ often smoothed near the seam of two spheres.^25,26^ Then a set of surface grid points is generated and each atomic sphere is divided into spherical triangular tesserae.^24^ Alternatively, the cavity can be bounded by an electron density isosurface corresponding to a certain ρ value.^27^

The nonelectrostatic effects that make an important contribution to Δ*G*_solv_^°^ include cavitation (formation of a cavity in the solvent), dispersion interactions, and specific interactions (e.g., the formation of strong hydrogen bonds in aqueous solution). Also, polarization of the solute by the solvent can be included in Δ*G*_corr_^°^.

Among the most efficient PCM methods are COSMO, developed by Klamt and Schüürmann,^28−32^ and the closely related C-PCM.^33,34^ In our recent papers,^35−37^ we presented a method for calculating solvation energies, dubbed uESE (universal easy solvation evaluation), based on the COSMO electrostatic term supplemented by an easily computed term Δ*G*_corr_^°^. For aqueous solutions, uESE adopts the following form for the correction term:
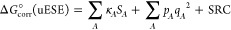
5where *S*_*A*_ are atomic surface areas; *q*_*A*_ are surface charges induced on these areas; κ_*A*_ and *p*_*A*_ are adjustable element-specific parameters. The first term, ∑_*A*_ κ_*A*_*S*_*A*_, corresponds to the cavitation energy plus solute–solvent dispersion interaction. This correction is similar to that used in many PCM^19,21,38^ and GB^14,16,39,40^ models. The ∑_*A*_ *p*_*A*_*q*_*A*_^2^ term are electrostatics and polarization effects; SRC is the short-range correction that describes hydrogen bonds as well as a part of the Pauli repulsion. The same idea was employed for nonaqueous solutions, although with a slightly modified form of the Δ*G*_corr_^°^ term.

The uESE method is suitable for neutral and ionic solutes in many different solvents. The molecular charge distribution in the uESE method is represented by discrete nuclear-centered charges {*Q*_*A*_} computed using the DFT method. The best results were achieved using CM5 charges,^41^ although other charge schemes give acceptable results as well.^35^ Since the CM5 charges are derived using the Hirshfeld charge scheme,^42^ the approach becomes time-consuming or even inapplicable to large-size systems. Thus, there is a need to develop a similar solvation method based on more readily available atomic charges for extended molecules including biopolymers. In this context, semiempirical methods that are about 3 orders of magnitude faster than DFT are ideal for calculating atomic charges in very large molecules.

Since the PM7 method^43^ is widely regarded as the most advanced semiempirical approach, in this work we present a solvation energy scheme based on geometries and atomic charges derived from PM7 calculations.

Much effort has recently been made to elaborate fast and accurate CS methods for the computational treatment of solvent effects for large molecules. The ddCOSMO linear-scaling algorithm proposed by Lipparini et al.^44−46^ enables very fast solution of the COSMO polarization equation. Its combination with the ESE-PM7 (easy solvation evaluation with PM7 charges) method will be of great benefit by treating extended systems.

## Method

The total solvation free energy in the ESE-PM7 method is evaluated from the electrostatic *E*_elst_ and the correction Δ*G*_corr_^°^ terms according to eq 1.

### Electrostatic Term

The calculation of *E*_elst_ for an *N*-atomic molecule in ESE-PM7 is very similar to that in our previous works^35−37^ and follows closely Klamt and Schüürmann’s original COSMO formulation.^28^

1. PM7 atomic charges {*Q*_*A*_} and coordinates {**R**_*A*_} are read in from an output of an unperturbed (gas-phase) Gaussian^47^ or MOPAC^48^ calculation.

2. The van der Waals cavity surface is constructed as a superposition of spheres with modified Bondi radii^49^ (*vide infra*) using the GEPOL93 algorithm^24^ with 32 tesserae per atom. This gives the coordinates {**r**_*i*_} for *M* surface points and the associated areas {*S*_*i*_}.

3. An *M* × *M* matrix **A** and *M* × *N* matrix **B** are constructed:
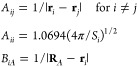
6

4. The working equation **Aq** = −**BQ** is solved with respect to the surface charges **q** = {*q*_*i*_}.

5. The resulting surface charges {*q*_*i*_} are used to compute the electrostatic energy:
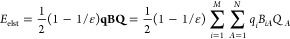
7where ε is the dielectric constant of the solvent. In contrast to our previous paper,^37^ we now use the same “Born-type”^50^ factor 1 – 1/ε for all solvents.

As before, in ESE-PM7 we employ a noniterative implementation of COSMO, based on gas-phase atomic charges. The waiver of the iterative polarization calculation leads to a large saving in computing time. The effects of solute polarization are approximately taken into account by the correction term Δ*G*_corr_^°^.

### Correction (Nonelectrostatic) Term

In the present approach, we employ a Δ*G*_corr_^°^ form (eq 4) slightly different from that given by eq 5. First of all, we discarded the SRC term used previously^36,37^ since it is not fully size consistent. Furthermore, the ∑_*A*_ *p*_*A*_*q*_*A*_^2^ term is replaced by a linear term, ∑_*A*_ *g*_*A*_*q*_*A*_. The working Δ*G*_corr_^°^ expression for polar protic solvents is as follows:
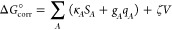
8

The *ζV* term implicitly describes the solute polarization and offers additional flexibility through the adjustable parameter ζ. Such a volume-dependent term was introduced by Andreussi et al.^38,51^ According to Hille et al.,^52^ the replacement of the volume by the solute polarizability leads to more accurate results.^52^ The latter approach is elegant but hardly applicable to real models, since calculating the solute polarizability is fairly expensive.

All solvents are divided into four classes: (A) water; (B) nonaqueous polar protic solvents (mainly alcohols and phenols); (C) polar aprotic solvents; (D) nonpolar solvents, i.e., those with ε < 9. The same partition was employed in our previous work.^37^ For polar protic solvents (classes A and B), Δ*G*_corr_^°^ is used in the form given by eq 8. To improve the flexibility of the model for solvent classes C and D, we have introduced an additional adjustable parameter—the solvent-dependent shift ξ_solv_*S* = ξ_solv_∑_*A*_ *S*_*A*_:
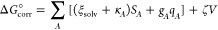
9

Note that the parameters κ_*A*_ and *g*_*A*_ are element-specific, whereas ξ_solv_ is solvent-specific but element-independent. In fact, ξ_solv_ is optional: omitting it will result in only a slight loss of accuracy.^37^

The atomic radii employed for cavity construction were modified. The same set of radii is used for all solvents, whereas κ_*A*_, *g*_*A*_, and ζ were fitted and tested separately for various classes of solvents. The nonlinear-least-squares fitting of van der Waals radii is described in the Supporting Information. Subsequently, Σ (eq 10) was minimized with respect to the adjustable parameters κ_*A*_, *g*_*A*_, and ζ:
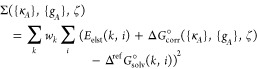
10where the index *i* runs over the molecules within a given subset and *k* runs over subsets; *w*_*k*_ is a suitable weighting factor. Each subset corresponds to a specific solvent. Also, different weighting factors were used for ions and neutral solutes. Since eqs 8 and 9 are linear with respect to the adjustable parameters κ_*A*_, *g*_*A*_, and ζ, the fitting is equivalent to solving a system of linear equations. Thus, the fitting problem is reduced to a single matrix inversion. Finally, the solvent-dependent parameter ξ_solv_ was found for each solvent by a separate least-squares fit.

Experimental values of Δ*G*_solv_^°^ from the Minnesota Solvation Database^53^ were used as a reference. The PM7 charges were calculated for molecular geometries optimized at the PM7 level using the Gaussian 16 program.^47^ The final parameters κ_*A*_, *g*_*A*_, ζ, and ξ_solv_ for each of the solvent classes A–D are given in Table 1.

**Table 1:** Van der Waals Radii *R*_*A*_^vdW^ (Å) and Parameters ζ (kcal/mol·Å^–3^), κ_*A*_ (kcal/mol·Å^–2^), and *g*_*A*_ (kcal/mol) for Main-Group Elements for Various Classes of Solvents^a^

	H	C	N	O	F	S	Cl	Br	I
*R*_*A*_^vdW^	0.90	1.80	1.96	1.52	1.47	2.34	1.75	1.85	1.98
Solvent Class A: Water; ζ = 0									
κ_*A*_	0.124	0.057	–0.036	0.070	0.069	0.038	–0.016	–0.047	–0.066
*g*_*A*_	10.0	–4.47	6.28	1.26	–3.67	–7.00	14.0	22.3	26.9
Solvent Class B: Polar Protic; ζ = −0.076									
κ_*A*_	0.037	0.097	0.017	–0.014	0.075	0.076	0.008	–0.034	–0.031
*g*_*A*_	3.45	–6.09	6.71	13.2	4.87	–13.4	16.3	23.3	17.7
Solvent Class C: Polar Aprotic; ζ = −0.178									
κ_*A*_	–0.119	0.165	0.148	–0.074	0.053	0.129	0.010	0.012	
*g*_*A*_	–7.99	8.88	11.0	32.8	18.9	18.5	32.0	33.0	
Solvent Class D: Nonpolar; ζ = −0.129									
κ_*A*_	–0.133	0.098	0.082	–0.065	0.057	0.106	0.061	0.048	0.052
*g*_*A*_	–39.4	–22.6	–17.2	–6.22	–10.3	–31.5	–21.8	–22.2	–29.3

a The ξ_solv_ values are given in the Supporting Information.

## Results and Discussion

The method was tested on several independent data sets that include both neutral and ionic solutes.

### Aqueous Solutions

In Table 2 we show a statistical evaluation of ESE-PM7 for aqueous solutions. Our test set of hydration energies Δ^ref^*G*_solv_^°^ (“MNSol”) includes all suitable entries presented in the Minnesota Solvation Database:^53^ 389 neutral molecules, 59 cations, and 80 anions. Five structures, for which PM7 yields qualitatively wrong geometries, were discarded. Also several other sets were tested: the data set of 464 solutes used by Kříž and Řezáč;^54^ the subset of 141 solutes from Mobley et al.’s data set^55^ (“Mobley”); Guthrie’s SAMPL1 “blind challenge” data set (“Blind”) containing 63 neutral pharmacologically important molecules;^56^ reduced Guthrie’s data set (53 molecules)^56^ used by Kříž and Řezáč^54^ (“SAMPL1”); reduced Guthrie’s SAMPL4 data set^57^ used by Kříž and Řezáč^54^ (“SAMPL4”); ionic data set (six cations and four anions) by Kříž and Řezáč^54^ (“C10”).

**Table 2:** Mean Signed Error (MSE), Mean Absolute Error (MAE), and Standard Deviation (SD) of the Hydration Free Energy in kcal/mol for Various Data Sets by the ESE-PM7 Method with Respect to Reference Values in Comparison with the PM7/COSMO2 Method^a^

	ESE-PM7			ESE-PM7(SN)			PM7/COSMO2
solutes	MSE	MAE	SD	MSE	MAE	SD	SD
MNSol (528)^b^	0.19	2.00	2.79	0.17	1.90	2.62	
neutrals (389)	–0.04	1.62	2.21	–0.10	1.48	1.96	
cations (59)	1.25	3.13	3.91	1.38	3.37	4.20	
anions (80)	0.52	3.01	4.03	0.58	2.85	3.72	
MNSol* (464)^c^	0.35	1.91	2.64	0.30	1.82	2.53	2.62^d^
neutrals (330)	0.08	1.46	1.90	–0.04	1.33	1.72	2.24^d^
cations (59)	1.25	3.13	3.91	1.38	3.37	4.20	2.87^d^
anions (75)	0.86	2.91	3.91	0.93	2.73	3.56	3.69^d^
Mobley141 (141)^c^	–0.43	1.25	1.72	–0.36	1.19	1.65	2.54^e^
Blind (63)^c^	–0.83	2.53	3.49	–0.53	2.30	2.94	
SAMPL1 (53)^c^	–0.24	2.35	3.50	–0.15	2.30	2.91	3.73^d^
SAMPL4 (42)^c^	–0.29	1.28	1.60	–0.25	1.34	1.59	1.92^d^
C10 (10)^c^	1.00	1.65	2.22	1.08	1.77	2.31	2.28^d^

a The complete lists of solutes, the calculated hydration free energies, and the reference values are given in the Supporting Information.

b Fitting set; for an explanation, see text.

c Test set; for an explanation, see text.

d Data from ref (54).

e Data from ref (54), “Mobley266” data set.

A comparison with the PM7/COSMO2 method^54^ done on the MNSol* data set of Kříž and Řezáč shows the same average performance of the methods with a standard deviation (SD; root-mean-square error) of about 2.6 kcal/mol. As seen, ESE-PM7 is clearly more advantageous for the neutral solutes but less accurate for ions. Also, ESE-PM7 is markedly more accurate when applied to the SAMPL1, SAMPL4, and Mobley data sets. For the C10 set of ions, ESE-PM7 and PM7/COSMO2 show similar performances.

A comparison with other solvation methods is provided in Table 3. With respect to the DFT-based SMD^19^ method, which has become a method of choice for routine Δ*G*_solv_^°^ computations,^47^ our noniterative ESE-PM7 method yields a substantially higher accuracy on the MNSol database (2.00 kcal/mol vs 2.53 kcal/mol). Although for the neutral solutes ESE-PM7 is not as accurate as SMD, it shows a much better performance for ions. With a mean absolute error (MAE) of about 3 kcal/mol, ESE-PM7 approaches the experimental accuracy. When tested on the “blind challenge” data set, the performance of ESE-PM7 is similar to that of SMD/DFT. Note that our DFT-based uESE method^37^ yields substantially more accurate results in all categories.

**Table 3:** Mean Signed Error (MSE), Mean Absolute Error (MAE), and Standard Deviation (SD) of the Hydration Free Energy in kcal/mol for Various Data Sets by the ESE-PM7 Method with Respect to Reference Values in Comparison with the uESE, SMD, and SMD/PM3 Methods^a^

	ESE-PM7			SMD/DFT		uESE/DFT		SMD/PM3	
solutes	MSE	MAE	SD	MSE	MAE	MSE	MAE	MSE	MAE
MNSol (528)	0.19	2.00	2.79	2.06^b^	2.53^b^	–0.06^c^	1.47^c^	1.3^d^	2.3^d^
neutrals (389)	–0.04	1.62	2.21	0.57^b^	1.15^b^	–0.14^c^	0.99^c^	1.1^d^	1.5^d^
cations (59)	1.25	3.13	3.91	3.44^b^	3.76^b^	0.12^c^	2.73^c^	5.3^d^	5.5^d^
anions (80)	0.52	3.01	4.03	8.92^b^	8.92^b^	0.19^c^	2.83^c^	–1.1^d^	3.2^d^

a The complete lists of solutes and the calculated hydration free energies and the reference values are given in the Supporting Information.

b Data from ref (36).

c Data from ref (37).

d Data from ref (58) (Table III).

In comparison with another semiempirical-based method, SMD/PM3^58^ (Table 3), ESE-PM7 gives a better overall performance on the MNSol data set (MAE of 2.00 kcal/mol vs 2.3 kcal/mol), with a substantial advantage for ions and a slightly higher MAE (1.62 kcal/mol vs 1.5 kcal/mol) for the neutral solutes.

**Figure 1:**
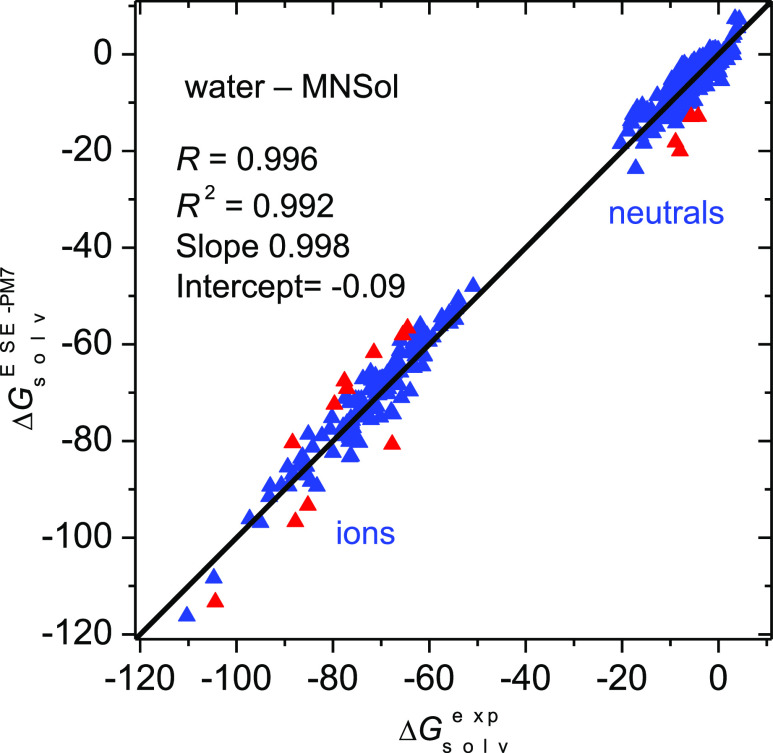
Hydration free energies (in kcal/mol) for 528 molecules and ions calculated by ESE-PM7 method versus experimental values. Red points denote outliers with a deviation greater than 7 kcal/mol.

In order to better evaluate the validity of our approach, Figure 1 shows hydration energies calculated for the MNSol data set, with failures (|Δ^calc^*G*_solv_^°^ – Δ^ref^*G*_solv_^°^| > 7 kcal/mol) indicated in red. There are four problematic neutral solutes as well as 11 ionic solutes. To sum up, the ESE-PM7 method yields more accurate estimates of hydration free energies than the PM7/COSMO2 method, but it is less accurate than the best DFT-based methods, in particular for neutral species. Nevertheless, an MAE of about 1.6 kcal/mol for the neutrals provides sufficient confidence for most practical cases.

Since in certain cases an accuracy improvement may be still desirable, we have considered the possibility of introducing separate parameters for sulfur and nitrogen in high oxidation states (the ESE-PM7(SN) model). Indeed, this extra flexibility results in markedly better estimates (see Table 2) for the neutral solutes and anions. A substantial improvement (by 0.22 kcal/mol in MAE) achieved for the “Blind” data set shows that the ESE-PM7 model allows for a more flexible adjustment when needed. However, we do not consider this scheme as a general approach, since the explicit dependence on the coordination of atoms may lead to wrong estimates for the system with untypical molecular configurations.

### Nonaqueous Solutions

The statistics for the ESE-PM7 method for nonaqueous solvents (solvent classes B–D) are listed in Tables 4–6. Table 4 shows the results for polar protic solvents in comparison with two established DFT-based solvation schemes, uESE-CM5 and SMD. The overall performance of the ESE-PM7 method is better than that of the standard SMD method, with an MAE 0.17 kcal/mol lower. In particular, a better performance of ESE-PM7 is found for Δ*G*_solv_^°^ in octanol, *m*-cresol, *sec*-butanol, and methanol. The advantage of ESE-PM7 is particularly pronounced for ionic solutes in methanol, with an MAE about 1.3 kcal/mol lower than that of SMD. For hexanol and isopropanol ESE-PM7 has roughly the same performance as SMD, whereas it is slightly less accurate for the other eight solvents but still gives good estimates of Δ*G*_solv_^°^, mostly within 1 kcal/mol.

**Table 4:** MSE and MAE of the Solvation Free Energy in kcal/mol for 14 Polar Protic Solvents Computed Using the ESE-PM7 Model in Comparison with uESE-CM5 and SMD (Total of 467 Entries)

	uESE-CM5^b^		SMD^c^		ESE-PM7		
solvent^a^	MSE	MAE	MSE	MAE	MSE	MAE	SD
octanol (247)	0.04	0.75	0.62	1.24	–0.03	1.02	1.40
heptanol (12)	0.25	0.47	0.72	0.74	0.20	0.85	0.95
*m*-cresol (7)	0.54	0.68	1.56	1.56	–0.09	1.13	1.33
benzyl alcohol (10)	0.09	0.35	0.42	0.66	–0.34	0.67	1.00
hexanol (14)	0.16	0.44	0.69	0.77	0.12	0.82	0.93
pentanol (22)	0.29	0.66	0.40	0.72	0.21	0.97	1.17
*sec*-butanol (9)	0.26	0.44	–0.23	0.53	–0.16	0.39	0.55
isobutanol (17)	0.09	0.63	–0.01	0.56	0.60	0.75	1.00
methoxyethanol (6)	–0.15	0.44	0.20	0.83	–0.99	0.99	1.21
butanol (21)	0.38	0.68	0.12	0.64	0.07	0.94	1.33
isopropanol (7)	–0.31	0.63	–0.91	1.02	–1.03	1.03	1.53
propanol (7)	–0.27	0.57	–0.66	0.81	–0.97	0.99	1.50
ethanol (8)	–0.60	0.82	–1.21	1.29	–1.33	1.33	1.65
methanol: cations (29)	–0.62	2.77	–0.32	2.44	0.14	2.18	2.86
methanol: anions (51)	–0.07	1.73	3.50	3.70	0.09	1.69	2.27
methanol: all ions (80)	–0.35	2.13	2.11	3.24	0.11	1.87	2.50
all polar protic (467)	0.02	0.93	0.74	1.44	–0.03	1.13	1.59

a The number of entries in the data set is given in parentheses.

b Data from ref (37).

c Data from ref (37) calculated using the Gaussian program package.^47^

Compared to the DFT-based uESE-CM5 method, ESE-PM7 does not achieve quite the same accuracy for neutral solutes, except for *sec*-butanol. However, for ions in methanol ESE-PM7 is more accurate, with an MAE about 0.25 kcal/mol lower. Overall, the ESE-PM7 results for protic solvents appear to be very convincing.

In comparison with the Δ*G*_solv_^°^ computed for methanol by Kromann et al. using the SMD approach in combination with several semiempirical methods,^58^ ESE-PM7 with an SD of 2.86 kcal/mol is superior to DFTB/SMD^†^, which shows the best performance yielding an SD of 4.7 kcal/mol.

**Figure 2:**
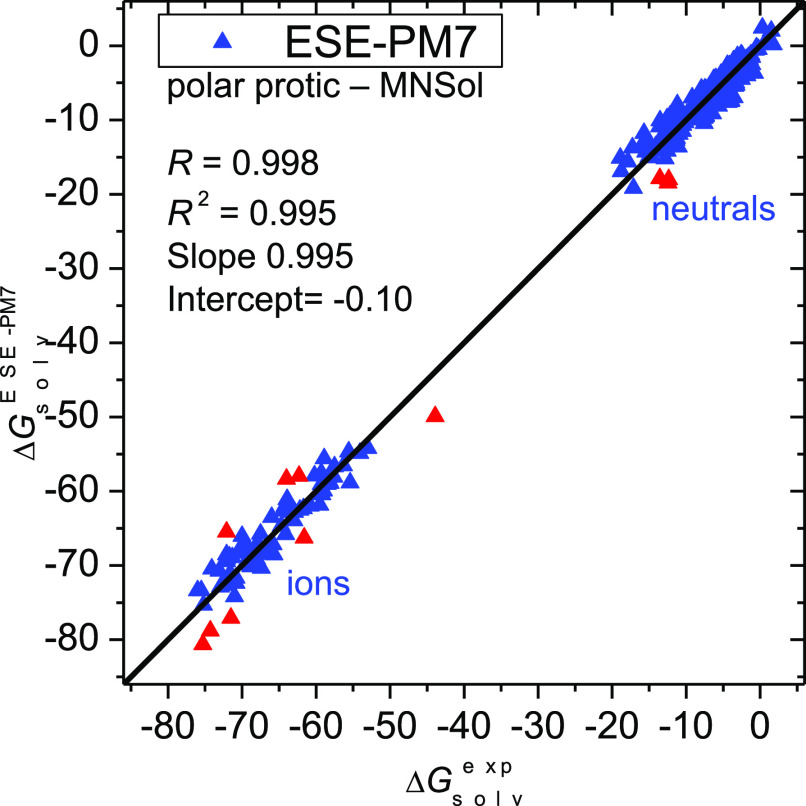
Solvation free energies (in kcal/mol) in nonaqueous protic solvents (class B) for 467 molecules and ions calculated by our ESE-PM7 method versus experimental values. Red points denote outliers with a deviation greater than 4 kcal/mol.

A good agreement between the experimental and ESE-PM7 calculated solvation free energies is illustrated in Figure 2. For only 12 solutes out of 467 is the deviation beyond 4 kcal/mol, and none deviates more than by 7 kcal/mol. The most problematic cases are ions i191 (2,4-dinitrophenolate), i047 (NH_4_^+^), and i148 (hydrazinium N_2_H_5_^+^). The found deviations are possibly due to the formation of strong hydrogen bonds.

Statistics for class C (polar aprotic) solvents in comparison with SMD and uESE-CM5 are presented in Table 5. According to these data, the general performance of ESE-PM7 is significantly better than that of SMD, with an MAE nearly 1 kcal/mol smaller. The advantage of the ESE-PM7 method is particularly pronounced for ionic solutes. For neutral solutes, both ESE-PM7 and SMD yield virtually the same error. Taken individually, ESE-PM7 is more accurate for neutral solutes in 12 solvents, while for the other eight (dichloroethane, MIBK, PhCN, MeNO_2_, MeCN, DMF, DMSO, NMF), SMD is somewhat superior. The accurate ESE-CM5 method is superior to ESE-PM7 for most polar aprotic solvents, with the exception of cyclohexanone, PhC(O)Me, MeCN (in the case of anions), and DMSO (in the case of cations). The ESE-PM7 method performs significantly better than all semiempirical methods considered by Kromann et al.:^58^ the standard deviations of DFTB/SMD showing the best performance are 12.5 kcal/mol for DMSO and 10.9 kcal/mol for acetonitrile, which are much larger than the SD of ESE-PM7. For other solvents, the best SD found in ref (58) is 2.7 kcal/mol (PM6/COSMO), which is still larger than that yielded by ESE-PM7.

**Table 5:** MSE and MAE of the Solvation Free Energy in kcal/mol for 20 Polar Aprotic Solvents Computed Using the ESE-PM7 Model in Comparison with uESE-CM5 and SMD (Total of 338 Entries)

	uESE-CM5^b^		SMD^c^		ESE-PM7		
solvent^a^	MSE	MAE	MSE	MAE	MSE	MAE	SD
bromoethane (7)	–0.04	0.61	–0.67	0.80	–0.09	0.72	1.05
2-methylpyridine (6)	0.09	0.53	–0.03	0.60	0.11	0.54	0.71
*o*-dichlorobenzene (11)	–0.06	0.41	–0.85	0.85	0.00	0.80	1.05
dichloroethane (39)	0.07	0.51	–0.18	0.49	0.06	0.57	0.77
4-methyl-2-pentanone (MIBK) (13)	0.21	1.01	–0.02	0.77	0.22	0.94	1.21
pyridine (7)	0.07	0.48	–0.19	0.65	0.00	0.61	0.91
cyclohexanone (10)	0.31	1.05	0.28	0.96	0.18	0.88	1.28
acetophenone (9)	0.20	0.69	–0.26	0.61	0.06	0.58	0.87
butanone (13)	0.00	0.81	–0.64	0.96	–0.09	0.90	1.16
benzonitrile (PhCN) (7)	–0.01	0.56	–0.64	0.77	–0.13	0.82	1.13
*o*-nitrotoluene (6)	0.01	0.16	–0.14	0.51	–0.06	0.41	0.60
nitroethane (7)	0.03	0.30	–0.37	0.58	–0.07	0.68	0.84
nitrobenzene (15)	0.01	0.22	–0.43	0.62	–0.07	0.54	0.73
acetonitrile (MeCN)							
neutral solutes (7)	0.65	0.69	–0.56	0.74	0.24	0.92	1.21
cations (39)	–0.28	2.01	7.65	7.93	–0.53	2.41	4.01
anions (30)	0.25	1.82	–2.63	3.01	0.60	1.60	1.96
all ions (69)	–0.18	1.97	3.18	5.79	–0.04	2.06	3.28
nitromethane (MeNO_2_) (7)	0.06	0.53	–0.46	0.85	–0.01	0.78	0.94
dimethylformamide (DMF) (7)	0.12	0.49	0.07	0.65	0.02	0.67	0.90
dimethylacetamide (DMA) (7)	0.11	0.54	–0.01	0.76	0.03	0.64	0.89
sulfolane (7)	0.12	0.54	1.42	1.48	0.04	0.86	1.04
dimethyl sulfoxide (DMSO)							
neutral solutes (7)	0.12	0.75	0.53	0.88	–1.03	1.62	2.59
cations (4)	0.15	2.25	8.31	8.31	0.30	1.85	2.53
anions (66)	–0.14	2.01	–1.69	3.55	–0.39	3.00	3.95
methylformamide (NMF) (7)	0.17	0.85	0.23	0.86	0.07	0.96	1.15
polar aprotic							
neutrals (199)	0.10	0.59	–0.18	0.73	–0.01	0.74	1.07
cations (43)	–0.24	2.03	7.71	7.96	–0.46	2.36	3.90
anions (96)	–0.02	1.95	–1.99	3.38	–0.08	2.57	3.46
all polar aprotic (338)	0.02	1.16	0.31	2.40	–0.08	1.47	2.45

a The number of entries in the data set is given in parentheses.

b Calculated using the Gaussian program package.^47^

c Data from ref (37) calculated using the Gaussian program package.^47^

**Figure 3:**
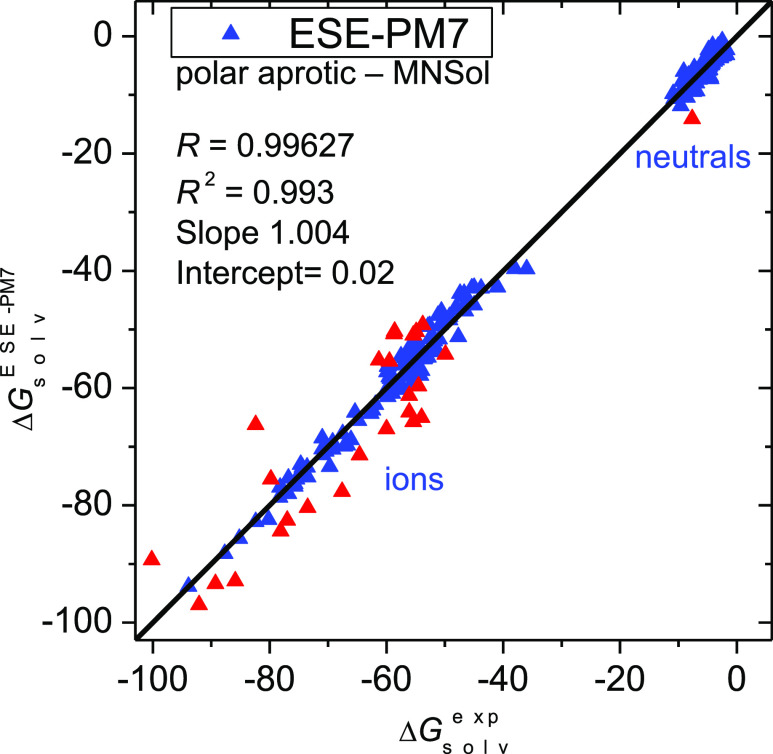
Solvation free energies (in kcal/mol) in polar aprotic solvents (class C) for 339 molecules and ions calculated by the ESE-PM7 method versus experimental values. Red points denote outliers with a deviation greater than 4 kcal/mol.

The situation for individual solutes in polar aprotic solvents is visualized in Figure 3. The only strongly deviating neutral solute is dimethyl sulfoxide (0503dim), belonging to the solutes (containing S–O bond) that give a large error for aqueous solutions as well (*vide supra*). Also various ions have significant errors (14 of 140 ions have an error beyond 6 kcal/mol) . Still, 99 ions have a deviation below 3 kcal/mol, and 80 of them even have a deviation below 2 kcal/mol.

The comparative statistics for class D (nonpolar) solvents are presented in Table 6. Both DFT-based reference methods, SMD and uESE-CM5, are quite accurate, with an MAE well below 0.7 kcal/mol. Our ESE-PM7 method shows a similar performance, with a total MAE of 0.77 kcal/mol. For 46 of 57 nonpolar solvents tested, ESE-PM7 yields a smaller error than SMD. Only for seven solvents does SMD work better, while for four solvents ESE-PM7 and SMD provide virtually the same performance (MAE).

**Table 6:** MSE and MAE of the Solvation Free Energy in kcal/mol for 57 Nonpolar Solvents Computed Using the ESE-PM7 Model in Comparison with uESE-CM5 and SMD (Total of 1554 Entries)

	uESE-CM5		SMD^b^		ESE-PM7		
solvent^a^	MSE	MAE	MSE	MAE	MSE	MAE	SD
pentane (26)	–0.01	0.28	–0.22	0.35	–0.01	0.34	0.50
hexane (59)	–0.01	0.36	–0.01	0.52	0.03	0.48	0.65
heptane (69)	0.02	0.37	0.22	0.55	0.07	0.42	0.60
isooctane (32)	–0.03	0.39	–0.32	0.45	–0.02	0.39	0.55
octane (38)	–0.01	0.28	0.06	0.43	0.01	0.36	0.50
nonane (26)	–0.02	0.22	0.04	0.37	0.01	0.18	0.22
decane (39)	–0.04	0.30	0.02	0.43	–0.02	0.29	0.47
undecane (13)	0.02	0.33	0.27	0.49	0.05	0.40	0.46
dodecane (8)	–0.12	0.34	0.06	0.30	0.02	0.18	0.21
cyclohexane (92)	–0.04	0.46	0.32	0.60	–0.01	0.49	0.68
perfluorobenzene (15)	0.06	0.36	0.49	0.56	0.03	0.38	0.46
pentadecane (9)	–0.12	0.31	0.39	0.48	0.04	0.13	0.16
hexadecane (198)	–0.05	0.45	0.32	0.68	–0.04	0.45	0.71
decalin (27)	0.02	0.30	0.57	0.67	0.02	0.37	0.51
carbon tetrachloride (79)	0.01	0.35	0.02	0.53	0.02	0.45	0.60
isopropyltoluene (6)	0.02	0.22	0.49	0.49	0.01	0.15	0.17
mesitylene (7)	0.02	0.25	0.09	0.54	0.05	0.39	0.50
tetrachloroethene (10)	0.02	0.26	0.57	0.74	0.01	0.18	0.21
benzene (75)	0.14	0.51	0.58	0.81	0.20	0.71	1.05
*sec*-butylbenzene (5)	0.04	0.16	0.12	0.25	0.04	0.19	0.21
*tert*-butylbenzene (14)	0.03	0.25	0.09	0.40	0.04	0.29	0.44
butylbenzene (10)	0.03	0.26	0.34	0.50	0.05	0.32	0.45
trimethylbenzene (11)	0.01	0.21	0.23	0.46	0.02	0.20	0.28
isopropylbenzene (19)	–0.02	0.25	–0.05	0.39	–0.04	0.28	0.46
toluene (51)	0.01	0.30	0.35	0.58	–0.01	0.39	0.52
triethylamine (7)	–0.03	0.58	0.77	0.98	–0.01	0.60	0.82
xylene (48)	0.03	0.34	0.45	0.60	0.01	0.39	0.53
ethylbenzene (29)	–0.03	0.30	0.17	0.47	–0.04	0.35	0.46
carbon disulfide (15)	–0.13	0.43	0.12	0.65	–0.14	0.71	1.16
tetralin (9)	–0.31	0.71	–1.08	1.30	–0.35	0.71	1.17
dibutyl ether (15)	0.06	0.60	0.61	0.79	0.06	0.58	0.86
diisopropyl ether (22)	0.03	0.91	0.41	0.76	–0.01	1.00	1.23
hexadecyl iodide (9)	–0.03	0.23	–0.07	0.42	–0.02	0.17	0.22
phenyl ether (6)	–0.09	0.35	–1.05	1.05	–0.11	0.48	0.76
fluorooctane (6)	–0.01	0.06	–0.48	0.48	–0.02	0.14	0.18
ethoxybenzene (7)	–0.09	0.34	0.08	0.45	–0.13	0.47	0.74
anisole (8)	–0.05	0.27	0.31	0.51	–0.08	0.46	0.75
diethyl ether (72)	–0.01	0.78	0.26	0.82	0.03	0.89	1.13
bromoform (12)	–0.04	0.24	0.72	0.72	–0.05	0.26	0.44
iodobenzene (20)	–0.07	0.45	–0.20	0.34	–0.06	0.50	0.75
chloroform (109)	0.00	0.64	0.28	0.79	0.06	0.81	1.15
dibromoethane (10)	–0.10	0.34	0.63	0.70	–0.08	0.34	0.47
butyl acetate (22)	0.08	0.56	1.08	1.15	0.08	0.66	0.92
bromooctane (5)	–0.04	0.16	–0.88	0.88	–0.05	0.27	0.32
bromobenzene (27)	–0.07	0.39	–0.48	0.51	–0.05	0.51	0.70
fluorobenzene (7)	–0.13	0.51	–0.79	0.83	–0.16	0.69	0.96
chlorobenzene (38)	–0.07	0.39	–0.63	0.65	–0.03	0.47	0.66
chlorohexane (11)	–0.01	0.17	–1.10	1.10	–0.02	0.30	0.40
ethyl acetate (24)	0.10	0.85	0.99	1.10	0.01	0.97	1.34
acetic acid (7)	0.06	0.46	2.37	2.37	–0.01	0.73	0.98
aniline (10)	0.22	0.70	0.76	0.78	0.20	0.84	1.23
dimethylpyridine (6)	–0.05	0.59	0.13	0.69	0.00	0.52	0.62
tetrahydrofuran (7)	–0.13	0.58	0.33	0.77	–0.17	0.72	0.97
decanol (11)	0.06	0.62	1.17	1.22	0.11	0.91	1.00
tributyl phosphate (16)	0.04	0.54	0.38	0.56	0.02	0.43	0.52
nonanol (10)	0.03	0.83	0.67	0.73	0.09	1.33	1.44
dichloromethane (11)	–0.18	0.67	–0.61	0.65	–0.29	0.83	1.14
all nonpolar (1554)	–0.01	0.44	0.22	0.65	0.01	0.51	0.77

a The number of entries in the data set is given in parentheses.

b Calculated using the Gaussian program package.^47^

Overall, ESE-PM7 is a reliable method with an MAE below 1 kcal/mol for all nonpolar solvents, with the exception of nonanol. In terms of the SD, there are just 10 solvents out of 57 that are just slightly beyond 1 kcal/mol.

**Figure 4:**
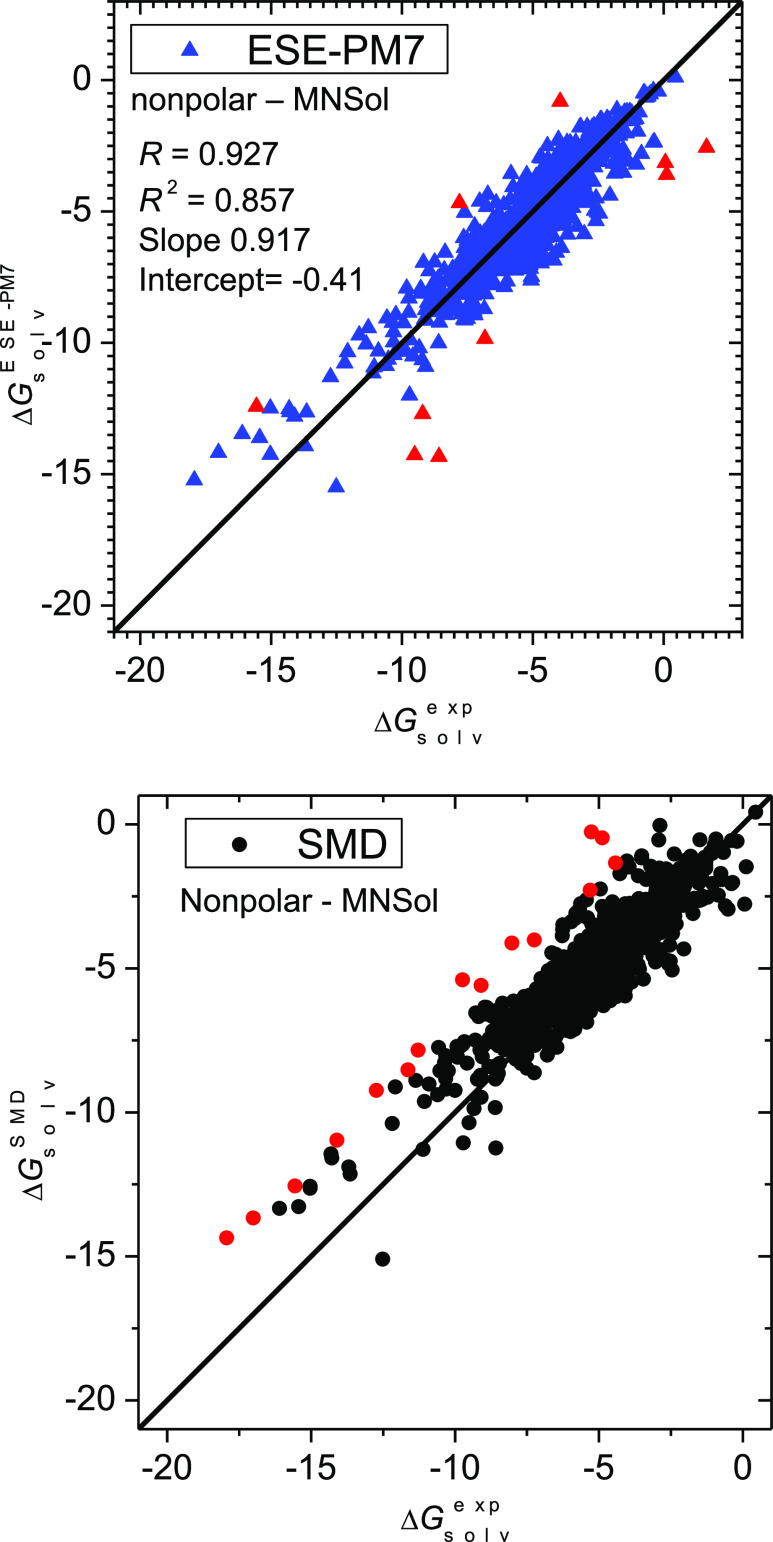
Solvation free energies (in kcal/mol) in nonpolar solvents (class D) for 1554 molecules calculated by the ESE-PM7 method versus experimental values. Red points denote outliers with a deviation greater than 3 kcal/mol. SMD results are given for comparison.

A graphical illustration of the solvation energies in nonpolar solutes is given in Figure 4. A strong deviation (>4 kcal/mol) is registered for 0400hyd (dihydrogen), 0447pho (diethyl-4-nitrophenylthiophosphonate), and 0441pho (dimethyl-4-nitrophenylthiophosphonate). The latter molecules are probably difficult due to a terminal phosphorus-coordinated sulfur, while H_2_ lacks an electrostatic contribution due to zero charges. Still, the overall performance of ESE-PM7 for nonpolar solvents is convincing: 1336 of 1554 molecules show an error below 1 kcal/mol, 1512 show an error within 2 kcal/mol, and only for 10 of them does the deviation exceed 3 kcal/mol. The corresponding numbers for the DFT-based SMD method are as follows: 1262, 1480, and 15 solutes, respectively.

## Conclusions

We have developed a simple and accurate method, ESE-PM7, for calculating the solvation free energies in aqueous and nonaqueous solutions. The very fast estimation of Δ*G*_solv_^°^ is due to (1) the use of geometries and atomic charges of solutes calculated with the semiempirical method PM7 and (2) the noniterative COSMO algorithm employed to calculate the solute–solvent electrostatic interaction. The parametrization of the nonelectrostatic term Δ*G*_corr_^°^ for 92 solvents divided into four classes (water, polar protic, polar aprotic, and nonpolar solvents) allows quite accurate predictions of Δ*G*_solv_^°^. The MAE of the ESE-PM7 method in aqueous solutions is found to be 1.62 kcal/mol for 389 neutral solutes and 3.06 kcal/mol for 139 ions. For nonaqueous solutions, ESE-PM7 provides more accurate estimates of Δ*G*_solv_^°^, with MAEs for neutral solutes of 0.97, 0.74, and 0.51 kcal/mol in polar protic, polar aprotic, and nonpolar solvents, respectively.

The ESE-PM7 method is intended for a standalone application and can be used to quickly estimate Δ*G*_solv_^°^ of extended molecular systems and to screen multiple data on solvation in drug design. Furthermore, it can be used in combination with PM7 for molecular dynamics simulations over a nanosecond scale.

## Data and Software Availability

The ESE-PM7 program executables and instructions are openly available for download at http://iqc.udg.es/~vybo/ESE-PM7.

## Supporting Information Available

Least-squares fitting of atomic radii; parameters ξ_solv_ for solvent classes C and D; ESE-PM7-calculated Δ*G*_solv_^°^ in various solvents (PDF)
