# Supplementary material for: Solvation Free Energies for Aqueous and Nonaqueous Solutions Computed Using PM7 Atomic Charges
Source: J Chem Inf Model. 2021 Sep 15;61(9):4544–53. doi: 10.1021/acs.jcim.1c00885 (PMC8479861; doi:10.1021/acs.jcim.1c00885)
Supplement: Supplementary file 1 — ci1c00885_si_001.pdf [file ci1c00885_si_001.pdf]

Solvation Free Energies For Aqueous And Non-Aqueous Solutions Computed Using PM7 Atomic Charges

Sergei F. Vyboishchikov[†] **and Alexander A. Voityuk**[†‡]
[†] Institut de Química Computacional i Catàlisi and Departament de Química, Universitat de Girona, Carrer Maria Aurèlia Capmany 69, 17003 Girona, Spain
[‡] Institució Catalana de Recerca i Estudis Avançats (ICREA), Passeig de Lluís Companys 23, 08010 Barcelona, Spain.

Supporting Information

Content

Part 1. Least-squares fitting of atomic radii .... p. 1
Part 2. Parameters ξ_{solv} for solvent classes **C** and **D** .... p. 2
Part 3. ESE-PM7-calculated solvation free energies in water........ p. 3
Part 4. ESE-PM7-calculated solvation free energies in non-aqueous solvents........ p. 27
Table S13. ESE-PM7 solvation free energies in non-aqueous polar protic solvents ....p. 27
Table S14. ESE-PM7 solvation free energies in polar aprotic solvents........p. 35
Table S15. ESE-PM7 solvation free energies in nonpolar solvents........p. 41

Part 1. Least-squares fitting of atomic radii

We adjusted the atomic van der Waals radii $\{R_A^{\mathrm{vdW}}\}$ by minimizing the sum of squared residuals Σ of the calculated ($E_{\mathrm{elst}} + \Delta G^{\circ}_{\mathrm{corr}}$) and reference ($\Delta^{\mathrm{ref}} G^{\circ}_{\mathrm{solv}}$) values of solvation free energy in water:

$$\Sigma(\{R_A^{\mathrm{vdW}}\}) = \sum_i \left(E_{\mathrm{elst}}(i) + \Delta G^{\circ}_{\mathrm{corr}}(\{R_A^{\mathrm{vdW}}\}, \{\kappa_A\}, \{g_A\}) - \Delta^{\mathrm{ref}} G^{\circ}_{\mathrm{solv}}(i)\right)^2$$

where the index i runs over the molecules within a fitting set. The non-linear minimization was done for a test set including 100 neutral molecules, 30 cations and 30 anions. Many nonlinear least-squares parametrization runs were carried out starting from fairly different initial radii values. The resulting optimized parameters with a low error function were tested in extensive survey calculations in order to choose the set with the most balanced results.

Part 2. Parameters ξ_{solv}.

Table S1. Optimized solvent-dependent parameters ξ_{solv} for class **C** and class **D** solvents in kcal/mol/Å^2.

Solvent class C – polar aprotic			
**Solvent**	ξ_{solv}	**Solvent**	ξ_{solv}
bromoethane	-0.00890	*o*-nitrotoluene	0.00358
2-methylpyridine	-0.00565	nitroethane	0.00302
o-dichlorobenzene	-0.00199	nitrobenzene	0.00351
dichloroethane	-0.00504	acetonitrile	0.00076
4-methyl-2-pentanone	-0.00884	nitromethane	0.00582
pyridine	-0.00321	dimethyl formamide	0.00265
cyclohexanone	-0.00536	dimethyl acetamide	0.00161
acetophenone	-0.00101	sulfolane	0.00519
butanone	-0.00202	dimethyl sulfoxide	0.00324
benzonitrile	-0.00277	methyl formamide	0.00416

Solvent class D – non–polar			
**Solvent**	ξ_{solv}	**Solvent**	ξ_{solv}
pentane	-0.00780	tetralin	0.00698
hexane	-0.00502	dibutyl ether	0.00320
heptane	-0.00533	diisopropyl ether	0.00096
isooctane	-0.00429	hexadecyl iodide	0.00869
octane	-0.00422	diphenyl ether	0.01040
nonane	-0.00529	fluoroctane	0.01140
decane	-0.00364	ethoxybenzene	0.01000
undecane	-0.00219	anisole	0.00821
dodecane	-0.00230	diethyl ether	0.00300
cyclohexane	-0.00300	bromoform	0.00441
perfluorobenzene	-0.01030	iodobenzene	0.00930
pentadecane	-0.00246	chloroform	0.00485
hexadecane	-0.00109	dibromoethane	0.00744
decalin	-0.00079	butyl acetate	0.00315
carbontet	-0.00462	bromooctane	0.01770
isopropyltoluene	-0.00485	bromobenzene	0.01150
mesitylene	-0.00578	fluorobenzene	0.01060
tetrachloroethene	-0.00664	chlorobenzene	0.01150
benzene	-0.00488	chlorohexane	0.01630
sec-butylbenzene	-0.00333	ethyl acetate	0.00244
tert-butylbenzene	-0.00266	acetic acid	0.01470
butylbenzene	-0.00337	aniline	0.01350
trimethylbenzene	-0.00338	dimethylpyridine	0.01330
isopropylbenzene	-0.00331	tetrahydrofuran	0.01310
toluene	-0.00578	decanol	0.00419
triethylamine	-0.00139	tributyl phosphate	0.00208
xylene	-0.00475	nonanol	0.00811
ethylbenzene	-0.00427	dichloromethane	0.01210
carbon disulfide	-0.00059		

Part 3. Solvation free energies in water. ESE-PM7 calculations versus experimental values from the Minnesota Solvation Database.[1,2]

Table S2. ESE-PM7 solvation free energies in water in kcal/mol.
MNSol - Neutrals(389)

Solute	ΔG^{o}_{solv}(exp)[1,2]	ΔG^{o}_{solv}(calc)	Formula
0001met	2.00	0.862	CH_4
0002eth	1.83	0.763	C_2H_6
0003pro	1.96	0.820	C_3H_8
0004nbu	2.08	0.888	C_4H_{10}
0005npe	2.33	0.940	C_5H_{12}
0006nhe	2.49	1.080	C_6H_{14}
0007nhe	2.62	1.068	C_7H_{16}
0008noc	2.89	1.183	C_8H_{18}
0010met	2.32	0.943	C_4H_{10}
0011dim	2.50	1.070	C_5H_{12}
0012met	2.52	1.026	C_6H_{14}
0013dim	2.88	1.032	C_7H_{16}
0014tri	2.85	1.229	C_8H_{18}
0016cyc	0.75	-0.107	C_3H_6
0017cyc	1.20	0.090	C_5H_{10}
0018cyc	1.23	0.624	C_6H_{12}
0019met	1.71	0.799	C_7H_{14}
0020cis	1.58	0.986	C_8H_{16}
0021eth	1.27	0.416	C_2H_4
0022pro	1.27	-0.082	C_3H_6
0023str	0.61	-0.565	C_4H_6
0024met	1.16	-0.470	C_4H_8
0025buta	1.38	-0.137	C_4H_8
0026cyc	0.56	-1.106	C_5H_8
0027pen	1.66	-0.006	C_5H_{10}
0028Epe	1.34	-0.305	C_5H_{10}
0029hex	1.68	0.320	C_6H_{12}
0030eth	-0.01	0.189	C_2H_2
0031pro	-0.31	-1.175	C_3H_4
0032but	-0.16	-0.825	C_4H_6
0033pen	0.01	-0.749	C_5H_8
0034hex	0.29	-0.413	C_6H_{10}
0035ben	-0.87	-2.493	C_6H_6
0036tol	-0.89	-2.701	C_7H_8
0037eth	-0.80	-2.518	C_8H_{10}
0038oxy	-0.90	-2.975	C_8H_{10}
0039mxy	-0.84	-2.889	C_8H_{10}
0040pxy	-0.81	-2.826	C_8H_{10}
0041nap	-2.39	-4.264	$C_{10}H_8$
0042ant	-4.23	-5.356	$C_{14}H_{10}$
0044met	-5.11	-4.339	CH_4O
0045eth	-5.01	-4.786	C_2H_6O
0046eth	-9.30	-8.952	$C_2H_6O_2$
0047pro	-4.83	-4.189	C_3H_8O
0048pro	-4.76	-3.942	C_3H_8O
0049but	-4.72	-4.235	$C_4H_{10}O$
0050met	-4.51	-4.303	$C_4H_{10}O$
0051cyc	-5.49	-4.216	$C_5H_{10}O$
0052pen	-4.47	-3.770	$C_5H_{12}O$

0053phe	-6.62	-5.681	C_6H_6O
0054hex	-4.36	-3.734	$C_6H_{14}O$
0055ocr	-5.87	-5.753	C_7H_8O
0056mcr	-5.49	-5.795	C_7H_8O
0057pcr	-6.14	-5.716	C_7H_8O
0058hep	-4.24	-3.665	$C_7H_{16}O$
0060dim	-1.92	-2.171	C_2H_6O
0061tet	-3.47	-3.361	C_4H_8O
0062dio	-5.05	-5.342	$C_4H_8O_2$
0063die	-1.76	-2.314	$C_4H_{10}O$
0064met	-1.66	-2.296	$C_4H_{10}O$
0065met	-2.01	-2.746	$C_4H_{10}O$
0066dim	-4.84	-4.419	$C_4H_{10}O_2$
0067but	-2.21	-2.538	$C_5H_{12}O$
0068ani	-2.45	-3.856	C_7H_8O
0070eth	-3.50	-3.779	C_2H_4O
0071proa	-3.44	-3.776	C_3H_6O
0072but	-3.18	-3.315	C_4H_8O
0073pen	-3.03	-3.286	$C_5H_{10}O$
0074ben	-4.02	-5.013	C_7H_6O
0075pro	-3.85	-3.632	C_3H_6O
0076but	-3.64	-3.521	C_4H_8O
0077cyc	-4.68	-3.373	C_5H_8O
0078pen	-3.53	-3.186	$C_5H_{10}O$
0079pen	-3.41	-3.566	$C_5H_{10}O$
0080hex	-3.29	-3.057	$C_6H_{12}O$
0081dim	-2.89	-2.963	$C_6H_{12}O$
0082hep	-3.04	-2.993	$C_7H_{14}O$
0083hep	-2.93	-2.661	$C_7H_{14}O$
0084met	-4.58	-4.807	C_8H_8O
0085non	-2.67	-2.549	$C_9H_{18}O$
0086eth	-6.70	-7.251	$C_2H_4O_2$
0087pro	-6.47	-6.667	$C_3H_6O_2$
0088but	-6.36	-6.190	$C_4H_8O_2$
0089pen	-6.16	-6.084	$C_5H_{10}O_2$
0090hex	-6.21	-5.983	$C_6H_{12}O_2$
0091met	-2.78	-5.160	$C_2H_4O_2$
0092ethb	-2.65	-5.246	$C_3H_6O_2$
0093met	-3.32	-4.781	$C_3H_6O_2$
0094met	-2.93	-4.352	$C_4H_8O_2$
0095eth	-3.10	-4.949	$C_4H_8O_2$
0096met	-2.83	-3.869	$C_5H_{10}O_2$
0097pro	-2.86	-4.326	$C_5H_{10}O_2$
0098met	-2.57	-3.756	$C_6H_{12}O_2$
0099but	-2.55	-4.301	$C_6H_{12}O_2$
0100met	-2.49	-3.740	$C_7H_{14}O_2$
0101pen	-2.45	-4.172	$C_7H_{14}O_2$
0103eth	-4.50	-2.078	C_2H_7N
0104dim	-4.29	-1.286	C_2H_7N
0105aze	-5.56	-1.893	C_3H_7N
0106pro	-4.39	-1.952	C_3H_9N
0107tri	-3.23	-0.510	C_3H_9N
0108pyr	-5.48	-1.981	C_4H_9N
0109pip	-7.40	-3.699	$C_4H_{10}N_2$
0110but	-4.29	-2.019	$C_4H_{11}N$

0111die	-4.07	-1.067	$C_4H_{11}N$
0112Nme	-7.77	-3.098	$C_5H_{12}N_2$
0113pen	-4.10	-1.885	$C_5H_{13}N$
0114NNd	-7.58	-2.227	$C_6H_{14}N_2$
0115dip	-3.66	-0.886	$C_6H_{15}N$
0116pyr	-4.70	-4.295	C_5H_5N
0117met	-5.57	-5.927	$C_5H_6N_2$
0118ani	-5.49	-6.491	C_6H_7N
0119met	-4.63	-4.285	C_6H_7N
0120met	-4.77	-4.451	C_6H_7N
0121met	-4.94	-4.573	C_6H_7N
0122Nme	-4.68	-5.737	C_7H_9N
0123dim	-4.86	-4.576	C_7H_9N
0124dim	-4.72	-4.415	C_7H_9N
0125dim	-4.60	-4.251	C_7H_9N
0126eth	-3.89	-3.475	C_2H_3N
0127pro	-3.85	-2.883	C_3H_5N
0128butb	-3.64	-2.697	C_4H_7N
0129ben	-4.10	-3.136	C_7H_5N
0130nit	-3.71	-6.632	$C_2H_5NO_2$
0131nit	-3.34	-6.069	$C_3H_7NO_2$
0132nit	-3.14	-5.537	$C_3H_7NO_2$
0133nit	-3.08	-5.900	$C_4H_9NO_2$
0134nit	-4.12	-5.318	$C_6H_5NO_2$
0135met	-3.59	-5.137	$C_7H_7NO_2$
0136met	-1.24	0.827	CH_4S
0137ethb	-1.30	0.657	C_2H_6S
0138pro	-1.05	0.845	C_3H_8S
0139thi	-2.55	-2.022	C_6H_6S
0140dim	-1.54	0.518	C_2H_6S
0141dim	-1.83	1.166	$C_2H_6S_2$
0142die	-1.43	-0.283	$C_4H_{10}S$
0143dip	-1.27	0.162	$C_6H_{14}S$
0144thi	-2.73	-2.595	C_7H_8S
0145pro	-5.08	-4.281	C_3H_6O
0146met	-6.77	-6.747	$C_3H_8O_2$
0147met	-6.55	-4.892	C_3H_9NO
0148but	0.04	-0.427	C_4H_4
0149mor	-7.17	-4.765	C_4H_9NO
0150mhy	-9.51	-7.952	$C_7H_6O_2$
0151phy	-10.48	-8.586	$C_7H_6O_2$
0153flu	-0.22	-1.545	CH_3F
0154dif	-0.11	-3.163	$C_2H_4F_2$
0157flu	-0.78	-1.584	C_6H_5F
0160chl	-0.56	-0.020	CH_3Cl
0161dic	-1.36	-0.925	CH_2Cl_2
0162tri	-1.07	-1.600	$CHCl_3$
0163chl	-0.63	-0.203	C_2H_5Cl
0165tri	-0.25	-1.028	$C_2H_3Cl_3$
0166tri	-1.95	-1.521	$C_2H_3Cl_3$
0167chla	-0.27	0.296	C_3H_7Cl
0168chl	-0.25	-0.328	C_3H_7Cl
0169chl	-0.59	-0.152	C_2H_3Cl
0170chl	-0.57	-0.786	C_3H_5Cl
0171Zdi	-1.17	-1.129	$C_2H_2Cl_2$

0172Edi	-0.76	-0.369	$C_2H_2Cl_2$
0173tri	-0.39	-1.222	C_2HCl_3
0174chl	-1.12	-2.029	C_6H_5Cl
0175odi	-1.36	-2.212	$C_6H_4Cl_2$
0176pdi	-1.01	-1.598	$C_6H_4Cl_2$
0177bro	-0.82	0.107	CH_3Br
0178dib	-2.11	-1.647	CH_2Br_2
0179tri	-1.98	-3.845	$CHBr_3$
0180bro	-0.70	0.294	C_2H_5Br
0182bro	-0.56	0.624	C_3H_7Br
0183bro	-0.48	0.473	C_3H_7Br
0184bro	-0.41	0.707	C_4H_9Br
0185bro	-0.08	0.870	$C_5H_{11}Br$
0186bro	-1.46	-2.699	C_6H_5Br
0187dib	-2.30	-3.377	$C_6H_4Br_2$
0197bro	1.79	-1.114	CF_3Br
0198chl	-0.77	-1.994	CH_2FCl
0199chl	-0.50	-2.505	CHF_2Cl
0200tet	3.16	4.103	CF_4
0201bro	-0.13	-2.124	C_2HF_3ClBr
0202bro	-1.95	-0.440	C_2H_4ClBr
0203bro	0.52	-1.736	C_2HF_4Br
0204tet	0.05	-1.824	C_2Cl_4
0205chl	0.06	-1.146	$C_2H_2F_3Cl$
0206tri	1.77	-0.086	$C_2F_3Cl_3$
0207tri	-4.31	-5.874	$C_2H_3OF_3$
0209chl	0.11	-3.705	$C_3H_2OF_5Cl$
0211tri	-4.16	-5.126	$C_3H_5OF_3$
0212hex	-3.77	-3.768	$C_3H_2OF_6$
0213bis	-3.92	-0.668	$C_4H_8SCl_2$
0214tri	-0.12	-1.981	$C_4H_5OF_3$
0215pbr	-7.13	-5.894	C_6H_5OBr
0216amm	-4.29	-3.757	H_3N
0217wat	-6.31	-6.994	H_2O
0218pho	0.60	-5.493	H_3P
0219hyd	-0.70	1.103	H_2S
0220tri	-8.70	-4.190	$C_3H_9O_4P$
0221tri	-7.80	-4.600	$C_6H_{15}O_4P$
0222tri	-6.10	-2.880	$C_9H_{21}O_4P$
0223die	-1.63	0.677	$C_4H_{10}S_2$
0225pipa	-5.11	-1.410	$C_5H_{11}N$
0227Nme	-6.34	-3.966	$C_5H_{11}NO$
0228met	-4.56	-2.360	CH_5N
0229hyd	-6.26	-5.525	H_4N_2
0230eth	-5.51	-5.632	$C_6H_8N_2$
0233ethb	-9.71	-9.130	C_2H_5NO
0234ENmb	-10.00	-8.507	C_3H_7NO
0235ZNmb	-10.00	-8.252	C_3H_7NO
0236oct	-4.09	-3.602	$C_8H_{18}O$
0237oct	-2.29	-3.173	$C_8H_{16}O$
0238met	-2.04	-3.663	$C_9H_{18}O_2$
0239oct	-2.88	-2.918	$C_8H_{16}O$
0240met	-3.91	-4.344	$C_8H_8O_2$
0242dii	-0.53	-2.851	$C_6H_{14}O$
0244tet	-3.12	-2.735	$C_5H_{10}O$

0245thi	-1.42	-0.347	C_4H_4S
0246eth	-2.22	-4.058	$C_8H_{10}O$
0400hyd	2.33	1.782	H_2
0401amia	-9.63	-9.228	$C_9H_{12}N_2O$
0402adn	-13.60	-16.214	$C_6H_7N_5$
0403thi	-10.40	-10.682	$C_6H_8N_2O_2$
0405hex	3.94	5.475	C_2F_6
0406oct	4.28	7.207	C_3F_8
0407tet	-1.15	-1.880	$C_2H_2Cl_4$
0408hex	-1.40	-2.351	C_2Cl_6
0409clb	0.07	-0.292	C_4H_9Cl
0410clp	0.07	0.400	$C_5H_{11}Cl$
0411chp	0.07	0.100	$C_5H_{11}Cl$
0412clt	-1.92	-3.281	C_7H_7Cl
0413clt	-1.15	-2.072	C_7H_7Cl
0414dcl	-2.73	-3.779	$C_{12}H_8Cl_2$
0415dcl	-2.45	-3.524	$C_{12}H_8Cl_2$
0416dcl	-1.99	-3.540	$C_{12}H_7Cl_3$
0417brp	-0.86	-0.428	C_3H_5Br
0418bri	-0.03	0.775	C_4H_9Br
0419brt	-2.37	-3.107	C_7H_7Br
0420pbr	-1.39	-2.657	C_7H_7Br
0421dfl	1.69	0.170	CF_2Cl_2
0422ftc	0.82	-0.930	$CFCl_3$
0423brt	-0.93	-2.994	CCl_3Br
0424clp	2.86	3.461	C_2F_5Cl
0425dbr	-9.00	-8.093	$C_7H_3NOBr_2$
0426dcl	-5.22	-3.186	$C_7H_3NCl_2$
0427dcl	-10.81	-9.938	$C_7H_5NSCl_2$
0428ami	-11.96	-12.589	$C_6H_3N_2O_2Cl_3$
0433pho	-6.61	-2.655	$C_4H_7O_4PCl_2$
0437pho	-6.92	-5.277	$C_9H_{13}O_3PS_2$
0438pho	-3.86	-2.167	$C_{10}H_{13}O_3PSCl_2$
0440pho	-7.28	-3.224	$C_9H_{12}O_4PCl$
0441pho	-7.62	-5.381	$C_8H_{10}NO_5PS$
0442pho	-4.09	-3.298	$C_{11}H_{15}O_3PSClBr$
0444pho	-5.06	-1.353	$C_8H_8O_3PSCl_3$
0445pho	-5.70	-2.116	$C_8H_8O_3PSCl_2Br$
0447pho	-6.27	-6.555	$C_{10}H_{14}NO_5PS$
0449pho	-5.10	-5.959	$C_{15}H_{14}NO_2PS$
0471dim	-5.22	-4.792	C_7H_9N
0506nit	-3.95	-7.328	CH_3NO_2
0571dim	-4.84	-4.689	C_7H_9N
0574eth	-4.74	-4.304	C_7H_9N
0939tet	3.04	-0.130	$C_4H_{12}Si$
n005	-5.31	-4.233	CH_6N_2
n006	-4.48	-3.168	$C_2H_8N_2$
n007	-13.80	-12.911	CH_4N_2O
n008	-10.90	-9.769	C_7H_7NO
n009	-5.56	-6.425	C_7H_9N
n010	-5.67	-6.719	C_7H_9N
n011	-5.55	-6.558	C_7H_9N
n013	-4.62	-5.408	$C_8H_{11}N$
n014	-3.58	-5.124	$C_8H_{11}N$
n015	-9.92	-11.017	$C_6H_8N_2$

n016	-9.72	-5.201	$C_2H_8N_2$
n017	-8.58	-3.981	H_2O_2
n018	-5.28	-2.473	CH_4O_2
n019	-5.32	-2.479	$C_2H_6O_2$
n191	-16.59	-13.010	$C_4H_4N_2O_2$
n200	-16.92	-11.162	$C_4H_3N_2O_2F$
n201	-15.46	-12.154	$C_5H_3N_2O_2F_3$
n202	-17.74	-12.474	$C_4H_3N_2O_2Cl$
n203	-18.17	-13.788	$C_4H_3N_2O_2Br$
test0001	-8.84	-14.276	$C_9H_{14}O_6$
test0004	1.07	0.617	$C_8H_4F_6$
test0005	-11.01	-8.497	$C_{10}H_{13}NO_2$
test0006	-9.76	-7.477	$C_{10}H_{13}NO$
test0007	-4.23	-2.496	$C_4H_8OCl_2$
test0008	-4.97	-9.623	$C_6H_{10}O_4$
test0009	-3.28	-5.422	$C_6H_{14}O_2$
test0011	-6.00	-8.430	$C_7H_{12}O_4$
test0012	-2.93	-4.313	$C_3H_8O_2$
test0013	-6.34	-10.238	$C_6H_{10}O_4$
test0014	-3.54	-5.333	$C_6H_{14}O_2$
test0016	-3.82	-5.932	$C_7H_6O_2$
test0017	-9.81	-7.762	$C_3H_4N_2$
test1001	-5.70	-3.603	$C_2H_4N_2O_6$
test1002	-5.00	-5.201	$C_3H_6N_2O_6$
test1003	-2.10	-1.522	$C_4H_9NO_3$
test1004	-1.80	-1.670	$C_4H_9NO_3$
test1005	-1.90	-1.423	$C_4H_9NO_3$
test1006	-8.20	-5.970	$C_2H_5NO_4$
test1007	-8.20	-7.888	$C_{14}H_{20}NO_2Cl$
test1008	-9.80	-7.307	$C_7H_{14}N_2O_2S$
test1009	-7.70	-10.286	$C_9H_{17}N_5S$
test1010	-10.00	-9.994	$C_{10}H_{12}N_3O_3PS_2$
test1011	-3.50	-4.493	$C_{13}H_{16}N_3O_4F_3$
test1012	-17.20	-23.664	$C_{16}H_{18}N_4O_7S$
test1013	-9.70	-9.734	$C_9H_{13}N_2O_2Br$
test1014	-9.00	-4.955	$C_9H_8NO_2SCl_3$
test1015	-9.50	-8.808	$C_{12}H_{11}NO_2$
test1016	-9.60	-9.194	$C_{12}H_{15}NO_3$
test1017	-6.50	-6.016	$C_{11}H_{16}O_2PS_3Cl$
test1018	-3.40	-4.430	$C_{10}H_6Cl_8$
test1019	-7.10	-2.061	$C_{12}H_{14}O_4PCl_3$
test1020	-14.00	-15.941	$C_{15}H_{15}N_4O_6SCl$
test1021	-1.50	-4.052	CNO_2Cl_3
test1022	-5.00	-2.082	$C_9H_{11}NO_3PSCl_3$
test1023	-5.70	-7.604	$C_{14}H_{17}NO_4PS_2Cl$
test1024	-6.50	-3.861	$C_{12}H_{21}N_2O_3PS$
test1025	-9.90	-6.849	$C_8H_6O_3Cl_2$
test1027	-5.70	-12.927	$C_{11}H_{13}N_4O_4F_3$
test1028	-6.20	-12.268	$C_{10}H_{12}N_2O_5$
test1029	-4.20	-12.879	$C_9H_6O_3SCl_6$
test1030	-5.50	-6.306	$C_{12}H_8OCl_6$
test1031	-6.10	-8.935	$C_9H_{22}O_4P_2S_4$
test1033	-2.60	-4.190	$C_{10}H_5Cl_7$
test1034	-5.20	-4.136	$C_9H_{14}O$
test1035	-5.40	-3.615	$C_6H_6Cl_6$

test1036	-8.20	-10.699	$C_{10}H_{19}O_6PS_2$
test1037	-10.70	-7.434	$C_5H_{10}N_2O_2S$
test1039	-15.50	-18.406	$C_{14}H_{15}N_5O_6S$
test1040	-8.00	-20.030	$C_{13}H_{19}N_3O_6S$
test1041	-6.00	-4.188	$C_3H_5NO_4$
test1043	-6.70	-6.548	$C_{10}H_{14}NO_5PS$
test1044	-3.60	-4.492	$C_{10}H_{21}NOS$
test1045	-4.40	-4.679	$C_7H_{17}O_2PS_3$
test1046	-2.50	-6.584	$C_{14}H_{16}N_3O_4F_3$
test1047	-8.40	-9.443	$C_{10}H_{19}N_5S$
test1048	-7.80	-7.271	$C_9H_9NOCl_2$
test1049	-16.40	-12.195	$C_{10}H_8N_3OCl$
test1050	-10.20	-12.239	$C_7H_{12}N_5Cl$
test1051	-20.30	-18.491	$C_{15}H_{16}N_4O_5S$
test1052	-11.10	-8.251	$C_9H_{13}N_2O_2Cl$
test1053	-6.70	-9.941	$C_{10}H_{19}N_5S$
test1054	-16.20	-16.924	$C_{12}H_{13}N_5O_6S_2$
test1055	-12.70	-8.613	$C_4H_8O_4PCl_3$
test1056	-3.30	-6.296	$C_{13}H_{16}N_3O_4F_3$
test1057	-4.10	-4.073	$C_{10}H_{21}NOS$
test1058	-11.20	-13.354	$C_{12}H_{10}N_4O_2$
test1059	-7.40	-10.378	$C_{20}H_{14}N_2O_2$
test1060	-8.90	-18.150	$C_{14}H_{12}N_4O_2$
test1061	-8.00	-7.362	$C_{14}H_9NO_2$
test1063	-9.40	-11.050	$C_{11}H_{18}N_4O_2$
test2001	-9.94	-9.531	$C_9H_8O_4$
test2003	-8.72	-7.347	$C_{11}H_{14}O_3$
test2004	-12.64	-12.535	$C_8H_{10}N_4O_2$
test2006	-15.83	-10.652	$C_4H_3N_2O_2Cl$
test2007	-18.06	-14.264	$C_3H_3N_3O_3$
test2010	-9.40	-10.134	$C_{13}H_8O_3F_2$
test2011	-9.20	-7.755	$C_9H_{10}O_3$
test2013	-8.42	-8.635	$C_{15}H_{13}O_2F$
test2015	-2.33	-2.562	C_6Cl_6
test2017	-7.00	-7.490	$C_{13}H_{18}O_2$
test2018	-18.72	-15.887	$C_4H_3N_2O_2I$
test2019	-10.78	-10.278	$C_{16}H_{14}O_3$
test2020	-9.51	-7.809	$C_8H_8O_3$
test2021	-10.21	-10.670	$C_{14}H_{14}O_3$
test2022	-9.45	-11.059	$C_6H_6N_2O_2$
test2023	3.43	7.387	C_4F_8
test2024	-5.22	-4.004	$C_6NO_2Cl_5$
test2025	-9.61	-5.724	$C_8H_5NO_2$
test2026	-9.37	-7.318	$C_{10}H_{12}O_3$
test2027	-8.61	-12.881	$C_4H_8O_2S$
test2029	-0.80	-2.848	$C_5H_9O_3F_3$
test3001	-14.83	-11.586	$C_8H_9NO_2$
test3002	-13.93	-11.474	$C_8H_9NO_2$
test3003	-12.75	-14.999	$C_{16}H_{14}O_3$
test3004	-11.61	-10.506	$C_8H_9NO_2$
test3005	-10.91	-9.981	$C_{10}H_{13}NO_2$
test3007	-10.32	-8.963	$C_8H_8O_3$
test3014	-9.15	-7.788	$C_8H_8O_3$
test3015	-8.93	-7.165	$C_8H_8O_3$
test3019	-6.71	-8.434	$C_{14}H_{12}NO_2Cl$

test3020	-6.30	-11.793	$C_{14}H_{11}NO_2Cl_2$
test3021	-5.68	-10.025	$C_{14}H_{10}NO_2F_3$
test4001	-1.73	-4.387	C_6H_5I
test4002	-2.49	-3.814	CH_2I_2
test4003	-0.89	-0.674	CH_3I
test4004	-0.72	-0.268	C_2H_5I
test4006	-0.59	0.085	C_3H_7I
test4007	-0.25	0.079	C_4H_9I
test4008	-0.12	0.225	$C_5H_{11}I$
test4009	-0.46	0.116	C_3H_7I

Table S3. ESE-PM7 solvation free energies in water in kcal/mol.
MNSol - Cations(59)

**Solute**	**ΔG^{o}_{solv}(exp)[1,2]**	**ΔG^{o}_{solv}(calc)**	**Formula**
c050	-76.60	-80.093	$CH_7O_2^+$
c051	-73.60	-73.441	$C_2H_9O_2^+$
c052	-66.30	-68.377	$C_2H_9O_2^+$
c053	-62.20	-59.191	$C_4H_{13}O_2^+$
c054	-66.30	-63.896	$C_3H_9O_2^+$
c056	-55.70	-55.679	$C_8H_{11}O_2^+$
c088	-87.80	-96.712	$H_5O_2^+$
i003	-76.40	-80.002	CH_6N^+
i004	-71.50	-72.711	$C_3H_{10}N^+$
i005	-69.60	-70.196	$C_3H_{10}N^+$
i006	-67.30	-65.942	$C_4H_{12}N^+$
i007	-68.70	-66.035	$C_6H_{14}N^+$
i008	-72.00	-72.720	$C_3H_8N^+$
i009	-68.60	-70.213	$C_2H_8N^+$
i010	-63.40	-61.606	$C_4H_{12}N^+$
i011	-60.50	-58.170	$C_6H_{16}N^+$
i012	-61.60	-58.561	$C_6H_{12}N^+$
i013	-61.10	-62.444	$C_3H_{10}N^+$
i014	-54.60	-52.674	$C_6H_{16}N^+$
i015	-50.90	-48.072	$C_9H_{22}N^+$
i018	-72.40	-69.380	$C_6H_8N^+$
i019	-70.30	-67.304	$C_7H_{10}N^+$
i020	-69.60	-67.156	$C_7H_{10}N^+$
i021	-69.80	-67.071	$C_7H_{10}N^+$
i023	-65.80	-71.011	$C_6H_9N_2^+$
i024	-62.60	-61.558	$C_7H_{10}N^+$
i025	-62.20	-57.400	$C_8H_{12}N^+$
i026	-57.20	-55.523	$C_8H_{12}N^+$
i027	-55.90	-53.628	$C_9H_{14}N^+$
i028	-54.00	-50.640	$C_{10}H_{16}N^+$
i029	-67.40	-66.980	$C_{10}H_{10}N^+$
i030	-70.90	-72.813	$C_2H_6N^+$
i031	-67.70	-67.146	$C_3H_8N^+$
i032	-66.00	-64.343	$C_4H_{10}N^+$
i033	-64.20	-61.762	$C_5H_{12}N^+$
i034	-63.30	-59.711	$C_6H_{14}N^+$
i035	-61.40	-64.494	$C_4H_6N^+$
i036	-61.10	-62.268	$C_5H_6N^+$
i037	-56.00	-55.112	$C_9H_8N^+$
i039	-66.00	-65.750	$C_4H_{11}N_2^+$
i040	-75.30	-71.369	$C_2H_4N^+$

i047	-85.20	-93.275	H_4N^+
i048	-84.60	-88.316	$H_5N_2^+$
i050	-93.00	-89.375	CH_5O^+
i051	-88.40	-80.366	$C_2H_7O^+$
i052	-79.70	-72.448	$C_2H_7O^+$
i053	-71.50	-61.739	$C_4H_{11}O^+$
i054	-77.10	-69.161	$C_3H_7O^+$
i056	-64.50	-57.869	$C_8H_9O^+$
i088	-110.30	-116.241	H_3O^+
i093	-71.20	-66.547	$C_7H_{10}NO^+$
i094	-75.90	-78.166	$C_6H_7N_2O_2^+$
i095	-69.60	-68.011	$C_4H_{10}NO^+$
i098	-73.90	-74.454	$C_2H_6NO^+$
i099	-67.20	-63.659	$C_7H_8NO^+$
i106	-64.50	-56.664	$C_2H_7S^+$
i112	-67.70	-65.407	$C_2H_7OS^+$
i125	-74.70	-71.403	$C_6H_7NCl^+$
i126	-74.10	-71.369	$C_6H_7NCl^+$

Table S4. ESE-PM7 solvation free energies in water in kcal/mol. MNSol[1,2] - Anions(80)

Solute	ΔG^{o}_{solv}(exp)	ΔG^{o}_{solv} (calc)	Formula
c001	-67.90	-73.886	$C_2H_3O^-$
c046b	-64.00	-69.656	CH_2NO^-
c065	-80.00	-82.369	$CH_5O_2^-$
c066	-78.50	-78.952	$C_2H_7O_2^-$
c067	-75.80	-77.267	$C_3H_9O_2^-$
c068	-76.00	-75.766	$C_3H_9O_2^-$
c069	-76.30	-75.350	$C_4H_{11}O_2^-$
c070	-72.10	-73.312	$C_4H_{11}O_2^-$
c071	-75.10	-74.345	$C_3H_7O_2^-$
c072	-75.40	-72.002	$C_7H_9O_2^-$
c073	-77.90	-77.058	$C_3H_9O_3^-$
c078	-73.30	-74.676	$C_2H_7O_3^-$
c082	-80.60	-77.609	$CH_5O_3^-$
c083	-77.10	-76.367	$C_2H_7O_3^-$
c089	-86.90	-85.056	$H_3O_2^-$
c091	-73.20	-74.874	$H_2O_3^-$
c114	-85.50	-85.257	H_2OF^-
c115	-67.50	-74.429	H_2OCl^-
c116	-63.20	-64.785	H_2OBr^-
c121	-68.00	-66.616	$C_2H_4O_2F_3^-$
c122	-61.60	-56.553	$C_3H_3O_2F_6^-$
i001	-76.50	-83.278	C_2H^-
i043	-72.20	-65.737	$CH_1N_2^-$
i044	-62.90	-62.395	$C_6H_6N^-$
i045	-54.60	-54.855	$C_{12}H_{10}N^-$
i046	-70.20	-75.025	CN^-
i058	-76.20	-83.129	CHO_2^-
i059	-77.60	-78.535	$C_2H_3O_2^-$
i060	-76.20	-77.423	$C_3H_5O_2^-$
i061	-74.60	-74.251	$C_6H_{11}O_2^-$
i062	-74.00	-75.080	$C_3H_3O_2^-$
i063	-68.50	-69.228	$C_3H_3O_3^-$
i064	-71.20	-69.970	$C_7H_5O_2^-$

i065	-95.00	-96.870	CH_3O^-
i066	-90.70	-89.160	$C_2H_5O^-$
i067	-88.30	-86.876	$C_3H_7O^-$
i068	-86.30	-83.213	$C_3H_7O^-$
i069	-84.20	-81.318	$C_4H_9O^-$
i070	-82.30	-78.948	$C_4H_9O^-$
i071	-86.60	-83.575	$C_3H_5O^-$
i072	-85.10	-78.643	$C_7H_7O^-$
i073	-89.40	-85.417	$C_3H_7O_2^-$
i074	-71.90	-67.460	$C_6H_5O^-$
i075	-70.20	-67.304	$C_7H_7O^-$
i076	-71.10	-67.126	$C_7H_7O^-$
i077	-72.00	-66.743	$C_7H_7O^-$
i078	-85.30	-87.045	$C_2H_5O_2^-$
i080	-73.80	-67.164	$C_6H_5O_2^-$
i081	-77.60	-67.611	$C_6H_5O_2^-$
i082	-93.20	-91.503	$CH_3O_2^-$
i083	-89.20	-89.333	$C_2H_5O_2^-$
i084	-76.50	-79.255	$C_2H_3O^-$
i085	-76.20	-76.192	$C_3H_5O^-$
i086	-73.70	-73.800	$C_5H_9O^-$
i089	-104.70	-108.360	HO^-
i090	-97.30	-96.099	HO_2^-
i091	-83.30	-89.371	O_2^-
i100	-60.10	-59.394	$C_6H_4NO_3^-$
i101	-61.90	-56.064	$C_6H_4NO_3^-$
i102	-57.80	-56.193	$C_6H_4NO_3^-$
i103	-76.50	-72.088	$CH_2NO_2^-$
i104	-57.40	-54.321	$C_6H_5N_2O_2^-$
i105	-80.20	-75.176	$C_2H_4NO^-$
i107	-73.80	-73.320	CH_3S^-
i108	-71.80	-72.047	$C_2H_5S^-$
i109	-70.50	-70.452	$C_3H_7S^-$
i110	-63.40	-64.009	$C_6H_5S^-$
i111	-72.10	-75.550	HS^-
i113	-67.70	-80.696	$C_2H_5OS^-$
i114	-104.40	-113.278	F^-
i115	-74.50	-80.288	Cl^-
i116	-68.30	-68.267	Br^-
i117	-54.10	-51.573	CCl_3^-
i118	-59.30	-58.493	$C_2O_2F_3^-$
i119	-69.70	-69.353	$C_2H_2O_2Cl^-$
i120	-62.30	-60.688	$C_2HO_2Cl_2^-$
i121	-77.50	-71.181	$C_2H_2OF_3^-$
i122	-65.50	-58.075	$C_3HOF_6^-$
i123	-66.10	-61.356	$C_6H_4OCl^-$
i124	-66.00	-59.212	$C_6H_4OCl^-$

Table S5. ESE-PM7 solvation free energies in water in kcal/mol.
MNSol*[3] - Neutrals(330)

**Solute**	**ΔG^o_{solv}(exp)**	**ΔG^o_{solv} (calc)**	**Formula**
0001met	2.00	0.862	CH_4
0002eth	1.83	0.763	C_2H_6
0003pro	1.96	0.820	C_3H_8
0004nbu	2.08	0.888	C_4H_{10}

0005npe	2.33	0.940	C_5H_{12}
0006nhe	2.49	1.080	C_6H_{14}
0007nhe	2.62	1.068	C_7H_{16}
0008noc	2.89	1.183	C_8H_{18}
0010met	2.32	0.943	C_4H_{10}
0011dim	2.50	1.070	C_5H_{12}
0012met	2.52	1.026	C_6H_{14}
0013dim	2.88	1.032	C_7H_{16}
0014tri	2.85	1.229	C_8H_{18}
0016cyc	0.75	-0.107	C_3H_6
0017cyc	1.20	0.090	C_5H_{10}
0018cyc	1.23	0.624	C_6H_{12}
0019met	1.71	0.799	C_7H_{14}
0020cis	1.58	0.986	C_8H_{16}
0021eth	1.27	0.416	C_2H_4
0022pro	1.27	-0.082	C_3H_6
0023str	0.61	-0.565	C_4H_6
0024met	1.16	-0.470	C_4H_8
0025buta	1.38	-0.137	C_4H_8
0026cyc	0.56	-1.106	C_5H_8
0027pen	1.66	-0.006	C_5H_{10}
0028Epe	1.34	-0.305	C_5H_{10}
0029hex	1.68	0.320	C_6H_{12}
0030eth	-0.01	0.189	C_2H_2
0031pro	-0.31	-1.175	C_3H_4
0032but	-0.16	-0.825	C_4H_6
0033pen	0.01	-0.749	C_5H_8
0034hex	0.29	-0.413	C_6H_{10}
0035ben	-0.87	-2.493	C_6H_6
0036tol	-0.89	-2.701	C_7H_8
0037eth	-0.80	-2.518	C_8H_{10}
0038oxy	-0.90	-2.975	C_8H_{10}
0039mxy	-0.84	-2.889	C_8H_{10}
0040pxy	-0.81	-2.826	C_8H_{10}
0041nap	-2.39	-4.264	$C_{10}H_8$
0042ant	-4.23	-5.356	$C_{14}H_{10}$
0044met	-5.11	-4.339	CH_4O
0045eth	-5.01	-4.786	C_2H_6O
0046eth	-9.30	-8.952	$C_2H_6O_2$
0047pro	-4.83	-4.189	C_3H_8O
0048pro	-4.76	-3.942	C_3H_8O
0049but	-4.72	-4.235	$C_4H_{10}O$
0050met	-4.51	-4.303	$C_4H_{10}O$
0051cyc	-5.49	-4.216	$C_5H_{10}O$
0052pen	-4.47	-3.770	$C_5H_{12}O$
0053phe	-6.62	-5.681	C_6H_6O
0054hex	-4.36	-3.734	$C_6H_{14}O$
0055ocr	-5.87	-5.753	C_7H_8O
0056mcr	-5.49	-5.795	C_7H_8O
0057pcr	-6.14	-5.716	C_7H_8O
0058hep	-4.24	-3.665	$C_7H_{16}O$
0060dim	-1.92	-2.171	C_2H_6O
0061tet	-3.47	-3.361	C_4H_8O
0062dio	-5.05	-5.342	$C_4H_8O_2$
0063die	-1.76	-2.314	$C_4H_{10}O$

0064met	-1.66	-2.296	$C_4H_{10}O$
0065met	-2.01	-2.746	$C_4H_{10}O$
0066dim	-4.84	-4.419	$C_4H_{10}O_2$
0067but	-2.21	-2.538	$C_5H_{12}O$
0068ani	-2.45	-3.856	C_7H_8O
0070eth	-3.50	-3.779	C_2H_4O
0071proa	-3.44	-3.776	C_3H_6O
0072but	-3.18	-3.315	C_4H_8O
0073pen	-3.03	-3.286	$C_5H_{10}O$
0074ben	-4.02	-5.013	C_7H_6O
0075pro	-3.85	-3.632	C_3H_6O
0076but	-3.64	-3.521	C_4H_8O
0077cyc	-4.68	-3.373	C_5H_8O
0078pen	-3.53	-3.186	$C_5H_{10}O$
0079pen	-3.41	-3.566	$C_5H_{10}O$
0080hex	-3.29	-3.057	$C_6H_{12}O$
0081dim	-2.89	-2.963	$C_6H_{12}O$
0082hep	-3.04	-2.993	$C_7H_{14}O$
0083hep	-2.93	-2.661	$C_7H_{14}O$
0084met	-4.58	-4.807	C_8H_8O
0085non	-2.67	-2.549	$C_9H_{18}O$
0086eth	-6.70	-7.251	$C_2H_4O_2$
0087pro	-6.47	-6.667	$C_3H_6O_2$
0088but	-6.36	-6.190	$C_4H_8O_2$
0089pen	-6.16	-6.084	$C_5H_{10}O_2$
0090hex	-6.21	-5.983	$C_6H_{12}O_2$
0091met	-2.78	-5.160	$C_2H_4O_2$
0092ethb	-2.65	-5.246	$C_3H_6O_2$
0093met	-3.32	-4.781	$C_3H_6O_2$
0094met	-2.93	-4.352	$C_4H_8O_2$
0095eth	-3.10	-4.949	$C_4H_8O_2$
0096met	-2.83	-3.869	$C_5H_{10}O_2$
0097pro	-2.86	-4.326	$C_5H_{10}O_2$
0098met	-2.57	-3.756	$C_6H_{12}O_2$
0099but	-2.55	-4.301	$C_6H_{12}O_2$
0100met	-2.49	-3.740	$C_7H_{14}O_2$
0101pen	-2.45	-4.172	$C_7H_{14}O_2$
0103eth	-4.50	-2.078	C_2H_7N
0104dim	-4.29	-1.286	C_2H_7N
0105aze	-5.56	-1.893	C_3H_7N
0106pro	-4.39	-1.952	C_3H_9N
0107tri	-3.23	-0.510	C_3H_9N
0108pyr	-5.48	-1.981	C_4H_9N
0109pip	-7.40	-3.699	$C_4H_{10}N_2$
0110but	-4.29	-2.019	$C_4H_{11}N$
0111die	-4.07	-1.067	$C_4H_{11}N$
0112Nme	-7.77	-3.098	$C_5H_{12}N_2$
0113pen	-4.10	-1.885	$C_5H_{13}N$
0114NNd	-7.58	-2.227	$C_6H_{14}N_2$
0115dip	-3.66	-0.886	$C_6H_{15}N$
0116pyr	-4.70	-4.295	C_5H_5N
0117met	-5.57	-5.927	$C_5H_6N_2$
0118ani	-5.49	-6.491	C_6H_7N
0119met	-4.63	-4.285	C_6H_7N
0120met	-4.77	-4.451	C_6H_7N

0121met	-4.94	-4.573	C_6H_7N
0122Nme	-4.68	-5.737	C_7H_9N
0123dim	-4.86	-4.576	C_7H_9N
0124dim	-4.72	-4.415	C_7H_9N
0125dim	-4.60	-4.251	C_7H_9N
0126eth	-3.89	-3.475	C_2H_3N
0127pro	-3.85	-2.883	C_3H_5N
0128butb	-3.64	-2.697	C_4H_7N
0129ben	-4.10	-3.136	C_7H_5N
0130nit	-3.71	-6.632	$C_2H_5NO_2$
0131nit	-3.34	-6.069	$C_3H_7NO_2$
0132nit	-3.14	-5.537	$C_3H_7NO_2$
0133nit	-3.08	-5.900	$C_4H_9NO_2$
0134nit	-4.12	-5.318	$C_6H_5NO_2$
0135met	-3.59	-5.137	$C_7H_7NO_2$
0136met	-1.24	0.827	CH_4S
0137ethb	-1.30	0.657	C_2H_6S
0138pro	-1.05	0.845	C_3H_8S
0139thi	-2.55	-2.022	C_6H_6S
0140dim	-1.54	0.518	C_2H_6S
0142die	-1.43	-0.283	$C_4H_{10}S$
0143dip	-1.27	0.162	$C_6H_{14}S$
0144thi	-2.73	-2.595	C_7H_8S
0145pro	-5.08	-4.281	C_3H_6O
0146met	-6.77	-6.747	$C_3H_8O_2$
0147met	-6.55	-4.892	C_3H_9NO
0148but	0.04	-0.427	C_4H_4
0149mor	-7.17	-4.765	C_4H_9NO
0150mhy	-9.51	-7.952	$C_7H_6O_2$
0151phy	-10.48	-8.586	$C_7H_6O_2$
0153flu	-0.22	-1.545	CH_3F
0154dif	-0.11	-3.163	$C_2H_4F_2$
0157flu	-0.78	-1.584	C_6H_5F
0160chl	-0.56	-0.020	CH_3Cl
0161dic	-1.36	-0.925	CH_2Cl_2
0162tri	-1.07	-1.600	$CHCl_3$
0163chl	-0.63	-0.203	C_2H_5Cl
0165tri	-0.25	-1.028	$C_2H_3Cl_3$
0166tri	-1.95	-1.521	$C_2H_3Cl_3$
0167chla	-0.27	0.296	C_3H_7Cl
0168chl	-0.25	-0.328	C_3H_7Cl
0169chl	-0.59	-0.152	C_2H_3Cl
0170chl	-0.57	-0.786	C_3H_5Cl
0171Zdi	-1.17	-1.129	$C_2H_2Cl_2$
0172Edi	-0.76	-0.369	$C_2H_2Cl_2$
0173tri	-0.39	-1.222	C_2HCl_3
0174chl	-1.12	-2.029	C_6H_5Cl
0175odi	-1.36	-2.212	$C_6H_4Cl_2$
0176pdi	-1.01	-1.598	$C_6H_4Cl_2$
0177bro	-0.82	0.107	CH_3Br
0178dib	-2.11	-1.647	CH_2Br_2
0179tri	-1.98	-3.845	$CHBr_3$
0180bro	-0.70	0.294	C_2H_5Br
0182bro	-0.56	0.624	C_3H_7Br
0183bro	-0.48	0.473	C_3H_7Br

0184bro	-0.41	0.707	C_4H_9Br
0185bro	-0.08	0.870	$C_5H_{11}Br$
0186bro	-1.46	-2.699	C_6H_5Br
0187dib	-2.30	-3.377	$C_6H_4Br_2$
0197bro	1.79	-1.114	CF_3Br
0198chl	-0.77	-1.994	CH_2FCl
0199chl	-0.50	-2.505	CHF_2Cl
0200tet	3.16	4.103	CF_4
0201bro	-0.13	-2.124	C_2HF_3ClBr
0202bro	-1.95	-0.440	C_2H_4ClBr
0203bro	0.52	-1.736	C_2HF_4Br
0204tet	0.05	-1.824	C_2Cl_4
0205chl	0.06	-1.146	$C_2H_2F_3Cl$
0206tri	1.77	-0.086	$C_2F_3Cl_3$
0207tri	-4.31	-5.874	$C_2H_3OF_3$
0209chl	0.11	-3.705	$C_3H_2OF_5Cl$
0211tri	-4.16	-5.126	$C_3H_5OF_3$
0212hex	-3.77	-3.768	$C_3H_2OF_6$
0213bis	-3.92	-0.668	$C_4H_8SCl_2$
0214tri	-0.12	-1.981	$C_4H_5OF_3$
0215pbr	-7.13	-5.894	C_6H_5OBr
0216amm	-4.29	-3.757	H_3N
0217wat	-6.31	-6.994	H_2O
0218pho	0.60	-5.493	H_3P
0219hyd	-0.70	1.103	H_2S
0220tri	-8.70	-4.190	$C_3H_9O_4P$
0221tri	-7.80	-4.600	$C_6H_{15}O_4P$
0222tri	-6.10	-2.880	$C_9H_{21}O_4P$
0223die	-1.63	0.677	$C_4H_{10}S_2$
0225pipa	-5.11	-1.410	$C_5H_{11}N$
0227Nme	-6.34	-3.966	$C_5H_{11}NO$
0228met	-4.56	-2.360	CH_5N
0229hyd	-6.26	-5.525	H_4N_2
0230eth	-5.51	-5.632	$C_6H_8N_2$
0233ethb	-9.71	-9.130	C_2H_5NO
0234ENmb	-10.00	-8.507	C_3H_7NO
0235ZNmb	-10.00	-8.252	C_3H_7NO
0236oct	-4.09	-3.602	$C_8H_{18}O$
0237oct	-2.29	-3.173	$C_8H_{16}O$
0238met	-2.04	-3.663	$C_9H_{18}O_2$
0239oct	-2.88	-2.918	$C_8H_{16}O$
0240met	-3.91	-4.344	$C_8H_8O_2$
0242dii	-0.53	-2.851	$C_6H_{14}O$
0244tet	-3.12	-2.735	$C_5H_{10}O$
0245thi	-1.42	-0.347	C_4H_4S
0246eth	-2.22	-4.058	$C_8H_{10}O$
0400hyd	2.33	1.782	H_2
0401amia	-9.63	-9.228	$C_9H_{12}N_2O$
0402adn	-13.60	-16.214	$C_6H_7N_5$
0403thi	-10.40	-10.682	$C_6H_8N_2O_2$
0405hex	3.94	5.475	C_2F_6
0406oct	4.28	7.207	C_3F_8
0407tet	-1.15	-1.880	$C_2H_2Cl_4$
0408hex	-1.40	-2.351	C_2Cl_6
0409clb	0.07	-0.292	C_4H_9Cl

0410clp	0.07	0.400	$C_5H_{11}Cl$
0411chp	0.07	0.100	$C_5H_{11}Cl$
0412clt	-1.92	-3.281	C_7H_7Cl
0413clt	-1.15	-2.072	C_7H_7Cl
0414dcl	-2.73	-3.779	$C_{12}H_8Cl_2$
0415dcl	-2.45	-3.524	$C_{12}H_8Cl_2$
0416dcl	-1.99	-3.540	$C_{12}H_7Cl_3$
0417brp	-0.86	-0.428	C_3H_5Br
0418bri	-0.03	0.775	C_4H_9Br
0419brt	-2.37	-3.107	C_7H_7Br
0420pbr	-1.39	-2.657	C_7H_7Br
0421dfl	1.69	0.170	CF_2Cl_2
0422ftc	0.82	-0.930	$CFCl_3$
0423brt	-0.93	-2.994	CCl_3Br
0424clp	2.86	3.461	C_2F_5Cl
0425dbr	-9.00	-8.093	$C_7H_3NOBr_2$
0426dcl	-5.22	-3.186	$C_7H_3NCl_2$
0427dcl	-10.81	-9.938	$C_7H_5NSCl_2$
0428ami	-11.96	-12.589	$C_6H_3N_2O_2Cl_3$
0433pho	-6.61	-2.655	$C_4H_7O_4PCl_2$
0437pho	-6.92	-5.277	$C_9H_{13}O_3PS_2$
0438pho	-3.86	-2.167	$C_{10}H_{13}O_3PSCl_2$
0440pho	-7.28	-3.224	$C_9H_{12}O_4PCl$
0441pho	-7.62	-5.381	$C_8H_{10}NO_5PS$
0442pho	-4.09	-3.298	$C_{11}H_{15}O_3PSClBr$
0444pho	-5.06	-1.353	$C_8H_8O_3PSCl_3$
0445pho	-5.70	-2.116	$C_8H_8O_3PSCl_2Br$
0447pho	-6.27	-6.555	$C_{10}H_{14}NO_5PS$
0449pho	-5.10	-5.959	$C_{15}H_{14}NO_2PS$
0471dim	-5.22	-4.792	C_7H_9N
0506nit	-3.95	-7.328	CH_3NO_2
0571dim	-4.84	-4.689	C_7H_9N
0574eth	-4.74	-4.304	C_7H_9N
n005	-5.31	-4.233	CH_6N_2
n006	-4.48	-3.168	$C_2H_8N_2$
n007	-13.80	-12.911	CH_4N_2O
n008	-10.90	-9.769	C_7H_7NO
n009	-5.56	-6.425	C_7H_9N
n010	-5.67	-6.719	C_7H_9N
n011	-5.55	-6.558	C_7H_9N
n013	-4.62	-5.408	$C_8H_{11}N$
n014	-3.58	-5.124	$C_8H_{11}N$
n015	-9.92	-11.017	$C_6H_8N_2$
n016	-9.72	-5.201	$C_2H_8N_2$
n017	-8.58	-3.981	H_2O_2
n018	-5.28	-2.473	CH_4O_2
n019	-5.32	-2.479	$C_2H_6O_2$
n191	-16.59	-13.010	$C_4H_4N_2O_2$
n200	-16.92	-11.162	$C_4H_3N_2O_2F$
n201	-15.46	-12.154	$C_5H_3N_2O_2F_3$
n202	-17.74	-12.474	$C_4H_3N_2O_2Cl$
n203	-18.17	-13.788	$C_4H_3N_2O_2Br$
test0001	-8.84	-14.276	$C_9H_{14}O_6$
test0004	1.07	0.617	$C_8H_4F_6$
test0005	-11.01	-8.497	$C_{10}H_{13}NO_2$

test0006	-9.76	-7.477	$C_{10}H_{13}NO$
test0007	-4.23	-2.496	$C_4H_8OCl_2$
test0008	-4.97	-9.623	$C_6H_{10}O_4$
test0009	-3.28	-5.422	$C_6H_{14}O_2$
test0011	-6.00	-8.430	$C_7H_{12}O_4$
test0012	-2.93	-4.313	$C_3H_8O_2$
test0013	-6.34	-10.238	$C_6H_{10}O_4$
test0014	-3.54	-5.333	$C_6H_{14}O_2$
test0016	-3.82	-5.932	$C_7H_6O_2$
test0017	-9.81	-7.762	$C_3H_4N_2$
test1058	-11.20	-13.354	$C_{12}H_{10}N_4O_2$
test2001	-9.94	-9.531	$C_9H_8O_4$
test2003	-8.72	-7.347	$C_{11}H_{14}O_3$
test2004	-12.64	-12.535	$C_8H_{10}N_4O_2$
test2006	-15.83	-10.652	$C_4H_3N_2O_2Cl$
test2007	-18.06	-14.264	$C_3H_3N_3O_3$
test2010	-9.40	-10.134	$C_{13}H_8O_3F_2$
test2011	-9.20	-7.755	$C_9H_{10}O_3$
test2013	-8.42	-8.635	$C_{15}H_{13}O_2F$
test2015	-2.33	-2.562	C_6Cl_6
test2017	-7.00	-7.490	$C_{13}H_{18}O_2$
test2018	-18.72	-15.887	$C_4H_3N_2O_2I$
test2019	-10.78	-10.278	$C_{16}H_{14}O_3$
test2020	-9.51	-7.809	$C_8H_8O_3$
test2021	-10.21	-10.670	$C_{14}H_{14}O_3$
test2022	-9.45	-11.059	$C_6H_6N_2O_2$
test2023	3.43	7.387	C_4F_8
test2024	-5.22	-4.004	$C_6NO_2Cl_5$
test2025	-9.61	-5.724	$C_8H_5NO_2$
test2026	-9.37	-7.318	$C_{10}H_{12}O_3$
test2027	-8.61	-12.881	$C_4H_8O_2S$
test2029	-0.80	-2.848	$C_5H_9O_3F_3$
test3001	-14.83	-11.586	$C_8H_9NO_2$
test3002	-13.93	-11.474	$C_8H_9NO_2$
test3003	-12.75	-14.999	$C_{16}H_{14}O_3$
test3004	-11.61	-10.506	$C_8H_9NO_2$
test3005	-10.91	-9.981	$C_{10}H_{13}NO_2$
test3007	-10.32	-8.963	$C_8H_8O_3$
test3014	-9.15	-7.788	$C_8H_8O_3$
test3015	-8.93	-7.165	$C_8H_8O_3$
test3019	-6.71	-8.434	$C_{14}H_{12}NO_2Cl$
test3020	-6.30	-11.793	$C_{14}H_{11}NO_2Cl_2$
test3021	-5.68	-10.025	$C_{14}H_{10}NO_2F_3$
test4001	-1.73	-4.387	C_6H_5I
test4002	-2.49	-3.814	CH_2I_2
test4003	-0.89	-0.674	CH_3I
test4004	-0.72	-0.268	C_2H_5I
test4006	-0.59	0.085	C_3H_7I
test4007	-0.25	0.079	C_4H_9I
test4008	-0.12	0.225	$C_5H_{11}I$
test4009	-0.46	0.116	C_3H_7I

Table S6. ESE-PM7 solvation free energies in water in kcal/mol. MNSol*[3] - Cations(59)

Solute	ΔG^o_{solv}(exp)	ΔG^o_{solv}(calc)	Formula

c050	-76.60	-80.093	$CH_7O_2^+$
c051	-73.60	-73.441	$C_2H_9O_2^+$
c052	-66.30	-68.377	$C_2H_9O_2^+$
c053	-62.20	-59.191	$C_4H_{13}O_2^+$
c054	-66.30	-63.896	$C_3H_9O_2^+$
c056	-55.70	-55.679	$C_8H_{11}O_2^+$
c088	-87.80	-96.712	$H_5O_2^+$
i003	-76.40	-80.002	CH_6N^+
i004	-71.50	-72.711	$C_3H_{10}N^+$
i005	-69.60	-70.196	$C_3H_{10}N^+$
i006	-67.30	-65.942	$C_4H_{12}N^+$
i007	-68.70	-66.035	$C_6H_{14}N^+$
i008	-72.00	-72.720	$C_3H_8N^+$
i009	-68.60	-70.213	$C_2H_8N^+$
i010	-63.40	-61.606	$C_4H_{12}N^+$
i011	-60.50	-58.170	$C_6H_{16}N^+$
i012	-61.60	-58.561	$C_6H_{12}N^+$
i013	-61.10	-62.444	$C_3H_{10}N^+$
i014	-54.60	-52.674	$C_6H_{16}N^+$
i015	-50.90	-48.072	$C_9H_{22}N^+$
i018	-72.40	-69.380	$C_6H_8N^+$
i019	-70.30	-67.304	$C_7H_{10}N^+$
i020	-69.60	-67.156	$C_7H_{10}N^+$
i021	-69.80	-67.071	$C_7H_{10}N^+$
i023	-65.80	-71.011	$C_6H_9N_2^+$
i024	-62.60	-61.558	$C_7H_{10}N^+$
i025	-62.20	-57.400	$C_8H_{12}N^+$
i026	-57.20	-55.523	$C_8H_{12}N^+$
i027	-55.90	-53.628	$C_9H_{14}N^+$
i028	-54.00	-50.640	$C_{10}H_{16}N^+$
i029	-67.40	-66.980	$C_{10}H_{10}N^+$
i030	-70.90	-72.813	$C_2H_6N^+$
i031	-67.70	-67.146	$C_3H_8N^+$
i032	-66.00	-64.343	$C_4H_{10}N^+$
i033	-64.20	-61.762	$C_5H_{12}N^+$
i034	-63.30	-59.711	$C_6H_{14}N^+$
i035	-61.40	-64.494	$C_4H_6N^+$
i036	-61.10	-62.268	$C_5H_6N^+$
i037	-56.00	-55.112	$C_9H_8N^+$
i039	-66.00	-65.750	$C_4H_{11}N_2^+$
i040	-75.30	-71.369	$C_2H_4N^+$
i047	-85.20	-93.275	H_4N^+
i048	-84.60	-88.316	$H_5N_2^+$
i050	-93.00	-89.375	CH_5O^+
i051	-88.40	-80.366	$C_2H_7O^+$
i052	-79.70	-72.448	$C_2H_7O^+$
i053	-71.50	-61.739	$C_4H_{11}O^+$
i054	-77.10	-69.161	$C_3H_7O^+$
i056	-64.50	-57.869	$C_8H_9O^+$
i088	-110.30	-116.241	H_3O^+
i093	-71.20	-66.547	$C_7H_{10}NO^+$
i094	-75.90	-78.166	$C_6H_7N_2O_2^+$
i095	-69.60	-68.011	$C_4H_{10}NO^+$
i098	-73.90	-74.454	$C_2H_6NO^+$
i099	-67.20	-63.659	$C_7H_8NO^+$

i106	-64.50	-56.664	$C_2H_7S^+$
i112	-67.70	-65.407	$C_2H_7OS^+$
i125	-74.70	-71.403	$C_6H_7NCl^+$
i126	-74.10	-71.369	$C_6H_7NCl^+$

Table S7. ESE-PM7 solvation free energies in water in kcal/mol. MNSol*[3] - Anions(75)

Solute	ΔG^o_{solv}(exp)	ΔG^o_{solv}(calc)	Formula
c001	-67.90	-73.886	$C_2H_3O^-$
c046b	-64.00	-69.656	CH_2NO^-
c065	-80.00	-82.369	$CH_5O_2^-$
c066	-78.50	-78.952	$C_2H_7O_2^-$
c067	-75.80	-77.267	$C_3H_9O_2^-$
c068	-76.00	-75.766	$C_3H_9O_2^-$
c069	-76.30	-75.350	$C_4H_{11}O_2^-$
c070	-72.10	-73.312	$C_4H_{11}O_2^-$
c071	-75.10	-74.345	$C_3H_7O_2^-$
c072	-75.40	-72.002	$C_7H_9O_2^-$
c073	-77.90	-77.058	$C_3H_9O_3^-$
c078	-73.30	-74.676	$C_2H_7O_3^-$
c082	-80.60	-77.609	$CH_5O_3^-$
c083	-77.10	-76.367	$C_2H_7O_3^-$
c089	-86.90	-85.056	$H_3O_2^-$
c114	-85.50	-85.257	H_2OF^-
c115	-67.50	-74.429	H_2OCl^-
c116	-63.20	-64.785	H_2OBr^-
c121	-68.00	-66.616	$C_2H_4O_2F_3^-$
c122	-61.60	-56.553	$C_3H_3O_2F_6^-$
i001	-76.50	-83.278	C_2H^-
i043	-72.20	-65.737	$CH_1N_2^-$
i044	-62.90	-62.395	$C_6H_6N^-$
i045	-54.60	-54.855	$C_{12}H_{10}N^-$
i046	-70.20	-75.025	CN^-
i058	-76.20	-83.129	CHO_2^-
i059	-77.60	-78.535	$C_2H_3O_2^-$
i060	-76.20	-77.423	$C_3H_5O_2^-$
i061	-74.60	-74.251	$C_6H_{11}O_2^-$
i062	-74.00	-75.080	$C_3H_3O_2^-$
i063	-68.50	-69.228	$C_3H_3O_3^-$
i064	-71.20	-69.970	$C_7H_5O_2^-$
i065	-95.00	-96.870	CH_3O^-
i066	-90.70	-89.160	$C_2H_5O^-$
i067	-88.30	-86.876	$C_3H_7O^-$
i068	-86.30	-83.213	$C_3H_7O^-$
i069	-84.20	-81.318	$C_4H_9O^-$
i070	-82.30	-78.948	$C_4H_9O^-$
i071	-86.60	-83.575	$C_3H_5O^-$
i072	-85.10	-78.643	$C_7H_7O^-$
i073	-89.40	-85.417	$C_3H_7O_2^-$
i074	-71.90	-67.460	$C_6H_5O^-$
i075	-70.20	-67.304	$C_7H_7O^-$
i076	-71.10	-67.126	$C_7H_7O^-$
i077	-72.00	-66.743	$C_7H_7O^-$
i078	-85.30	-87.045	$C_2H_5O_2^-$
i080	-73.80	-67.164	$C_6H_5O_2^-$

i081	-77.60	-67.611	$C_6H_5O_2^-$
i082	-93.20	-91.503	$CH_3O_2^-$
i083	-89.20	-89.333	$C_2H_5O_2^-$
i084	-76.50	-79.255	$C_2H_3O^-$
i085	-76.20	-76.192	$C_3H_5O^-$
i086	-73.70	-73.800	$C_5H_9O^-$
i089	-104.70	-108.360	HO^-
i090	-97.30	-96.099	HO_2^-
i100	-60.10	-59.394	$C_6H_4NO_3^-$
i101	-61.90	-56.064	$C_6H_4NO_3^-$
i102	-57.80	-56.193	$C_6H_4NO_3^-$
i103	-76.50	-72.088	$CH_2NO_2^-$
i104	-57.40	-54.321	$C_6H_5N_2O_2^-$
i105	-80.20	-75.176	$C_2H_4NO^-$
i107	-73.80	-73.320	CH_3S^-
i108	-71.80	-72.047	$C_2H_5S^-$
i109	-70.50	-70.452	$C_3H_7S^-$
i110	-63.40	-64.009	$C_6H_5S^-$
i111	-72.10	-75.550	HS^-
i113	-67.70	-80.696	$C_2H_5OS^-$
i117	-54.10	-51.573	CCl_3^-
i118	-59.30	-58.493	$C_2O_2F_3^-$
i119	-69.70	-69.353	$C_2H_2O_2Cl^-$
i120	-62.30	-60.688	$C_2HO_2Cl_2^-$
i121	-77.50	-71.181	$C_2H_2OF_3^-$
i122	-65.50	-58.075	$C_3HOF_6^-$
i123	-66.10	-61.356	$C_6H_4OCl^-$
i124	-66.00	-59.212	$C_6H_4OCl^-$

Table S8. ESE-PM7 solvation free energies in water in kcal/mol. Mobley141[4] (141)

Solute	ΔG^o_{solv}(exp)	ΔG^o_{solv}(calc)	Formula
111_trimethoxyethane	-4.42	-6.796	$C_5H_{12}O_3$
1122_tetrachloroethane	-2.47	-2.631	$C_2H_2Cl_4$
11_diacetoxyethane	-4.97	-9.621	$C_6H_{10}O_4$
11_dichloroethane	-0.84	-0.965	$C_2H_4Cl_2$
11_dichloroethene	0.25	-0.437	$C_2H_2Cl_2$
1234_tetrachlorobenzene	-1.34	-2.309	$C_6H_2Cl_4$
1235_tetrachlorobenzene	-1.62	-1.775	$C_6H_2Cl_4$
123_trichlorobenzene	-1.24	-2.274	$C_6H_3Cl_3$
123_trimethylbenzene	-1.21	-3.258	C_9H_{12}
1245_tetrachlorobenzene	-1.34	-1.846	$C_6H_2Cl_4$
124_trichlorobenzene	-1.12	-1.841	$C_6H_3Cl_3$
124_trimethylbenzene	-0.86	-3.168	C_9H_{12}
12_dibromoethane	-2.33	-0.477	$C_2H_4Br_2$
12_dichloroethane	-1.79	-1.552	$C_2H_4Cl_2$
12_dichloropropane	-1.27	-1.509	$C_3H_6Cl_2$
135_trichlorobenzene	-0.78	-1.166	$C_6H_3Cl_3$
135_trimethylbenzene	-0.90	-3.171	C_9H_{12}
13_dichlorobenzene	-0.98	-1.603	$C_6H_4Cl_2$
13_dichloropropane	-1.89	-0.740	$C_3H_6Cl_2$
13_dimethylnaphthalene	-2.47	-4.663	$C_{12}H_{12}$
14_dichlorobutane	-2.32	-0.700	$C_4H_8Cl_2$
14_dimethylnaphthalene	-2.82	-4.783	$C_{12}H_{12}$
1_bromoheptane	0.34	0.891	$C_7H_{15}Br$

1_bromohexane	0.18	0.904	$C_6H_{13}Br$
1_bromooctane	0.52	1.064	$C_8H_{17}Br$
1_chlorobutane	-0.16	0.364	C_4H_9Cl
1_chloroheptane	0.29	0.585	$C_7H_{15}Cl$
1_chlorohexane	0.00	0.334	$C_6H_{13}Cl$
1_ethylnaphthalene	-2.40	-4.322	$C_{12}H_{12}$
1_iodoheptane	0.27	0.296	$C_7H_{15}I$
1_iodohexane	0.08	0.274	$C_6H_{13}I$
1_methylcyclohexene	-8.41	-1.416	C_7H_{12}
1_methyl_imidazole	-2.89	-6.896	$C_4H_6N_2$
1_methylnaphthalene	0.67	-4.602	$C_{11}H_{10}$
1_methyl_pyrrole	-2.44	-4.621	C_5H_7N
1_naphthol	-7.67	-7.326	$C_{10}H_8O$
1_naphthylamine	-7.28	-8.642	$C_{10}H_9N$
1_nitropentane	-2.82	-5.833	$C_5H_{11}NO_2$
225_trimethylhexane	2.93	1.250	C_9H_{20}
22_dimethylbutane	2.51	1.115	C_6H_{14}
22_dimethylpentane	2.88	1.116	C_7H_{16}
234_trimethylpentane	2.56	1.205	C_8H_{18}
23_dimethylbuta_13_diene	0.40	-0.842	C_6H_{10}
23_dimethylbutane	2.34	1.106	C_6H_{14}
23_dimethylnaphthalene	-2.78	-4.519	$C_{12}H_{12}$
23_dimethylpentane	2.52	1.035	C_7H_{16}
23_dimethylphenol	-6.16	-5.887	$C_8H_{10}O$
23_dimethylpyridine	-4.82	-4.582	C_7H_9N
24_dimethylpentan_3_one	-2.74	-2.871	$C_7H_{14}O$
24_dimethylphenol	-6.01	-5.762	$C_8H_{10}O$
25_dimethylphenol	-5.91	-5.699	$C_8H_{10}O$
25_dimethyltetrahydrofuran	-2.92	-3.583	$C_6H_{12}O$
26_dimethylaniline	-5.21	-6.415	$C_8H_{11}N$
26_dimethylnaphthalene	-2.63	-4.286	$C_{12}H_{12}$
26_dimethylphenol	-5.26	-5.646	$C_8H_{10}O$
2_bromo_2_methylpropane	0.84	0.620	C_4H_9Br
2_butoxyethanol	-6.25	-6.723	$C_6H_{14}O_2$
2_chloro_111_trimethoxyethane	-4.59	-7.933	$C_5H_{11}O_3Cl$
2_chloro_2_methylpropane	1.09	-0.576	C_4H_9Cl
2_chloroaniline	-4.91	-6.187	C_6H_6NCl
2_chlorophenol	-4.55	-4.670	C_6H_5OCl
2_chloropyridine	-4.39	-4.191	C_5H_4NCl
2_ethoxyethanol	-6.69	-7.138	$C_4H_{10}O_2$
2_ethylpyridine	-4.33	-4.101	C_7H_9N
2_ethyltoluene	-1.04	-2.884	C_9H_{12}
2_fluorophenol	-5.29	-4.164	C_6H_5OF
2_iodophenol	-6.20	-6.970	C_6H_5OI
2_isobutylpyrazine	-5.04	-5.246	$C_8H_{12}N_2$
2_methoxy_111_trimethoxyethane	-5.73	-7.597	$C_6H_{14}O_4$
2_methoxyaniline	-6.12	-7.119	C_7H_9NO
2_methoxyphenol	-5.57	-5.985	$C_7H_8O_2$
2_methyl_but_2_ene	1.31	-1.018	C_5H_{10}
2_methylbut_2_ene	1.31	-1.018	C_5H_{10}
2_methylbuta_13_diene	0.68	-0.508	C_5H_8
2_methylbutan_1_ol	-4.42	-3.593	$C_5H_{12}O$
2_methylbutan_2_ol	-4.43	-4.045	$C_5H_{12}O$
2_methylbutane	2.38	0.968	C_5H_{12}
2_methylhexane	2.93	1.072	C_7H_{16}

2_methylpent_1_ene	1.47	-0.055	C_6H_{12}
2_methylpentan_2_ol	-3.92	-3.862	$C_6H_{14}O$
2_methylpentan_3_ol	-3.88	-3.403	$C_6H_{14}O$
2_methylpropan_1_ol	-4.50	-3.797	$C_4H_{10}O$
2_methylpropan_2_ol	-4.47	-4.174	$C_4H_{10}O$
2_methyltetrahydrofuran	-3.30	-3.300	$C_5H_{10}O$
2_methylthiophene	-1.38	-1.086	C_5H_6S
2_naphthol	-8.11	-7.226	$C_{10}H_8O$
2_naphthylamine	-7.47	-8.262	$C_{10}H_9N$
2_nitroaniline	-7.37	-9.884	$C_6H_6N_2O_2$
2_nitrophenol	-4.58	-10.157	$C_6H_5NO_3$
2_phenylethanol	-6.79	-6.664	$C_8H_{10}O$
2_propoxyethanol	-6.40	-6.646	$C_5H_{12}O_2$
333_trimethoxypropionitrile	-6.40	-8.514	$C_6H_{11}NO_3$
33_dimethylpentane	2.56	1.276	C_7H_{16}
34_dimethylphenol	-6.50	-6.012	$C_8H_{10}O$
35_dimethylphenol	-6.27	-5.991	$C_8H_{10}O$
3_acetylpyridine	-8.26	-6.727	C_7H_7NO
3_chloroaniline	-5.82	-6.401	C_6H_6NCl
3_chlorophenol	-6.62	-5.151	C_6H_5OCl
3_chloropyridine	-4.01	-3.934	C_5H_4NCl
3_cyanophenol	-9.65	-6.037	C_7H_5NO
3_cyanopyridine	-6.75	-4.981	$C_6H_4N_2$
3_ethylphenol	-6.25	-5.618	$C_8H_{10}O$
3_ethylpyridine	-4.59	-4.319	C_7H_9N
3_formylpyridine	-7.10	-6.707	C_6H_5NO
3_methoxyaniline	-7.29	-8.233	C_7H_9NO
3_methoxyphenol	-7.66	-6.896	$C_7H_8O_2$
3_methyl_1h_indole	-5.88	-7.438	C_9H_9N
3_methyl_but_1_ene	1.83	0.379	C_5H_{10}
3_methylbut_1_ene	1.82	0.379	C_5H_{10}
3_methylbutan_1_ol	-4.42	-3.765	$C_5H_{12}O$
3_methylbutan_2_one	-3.24	-3.279	$C_5H_{10}O$
3_methylbutanoic_acid	-6.09	-5.752	$C_5H_{10}O_2$
3_methylheptane	2.97	1.087	C_8H_{18}
3_methylhexane	2.71	0.962	C_7H_{16}
3_methylpentane	2.51	0.996	C_6H_{14}
3_nitroaniline	-8.84	-9.205	$C_6H_6N_2O_2$
3_nitrophenol	-9.62	-8.043	$C_6H_5NO_3$
3_nitrotoluene	-3.45	-4.988	$C_7H_7NO_2$
3_phenylpropanol	-6.92	-7.181	$C_9H_{12}O$
4_acetylpyridine	-7.62	-6.251	C_7H_7NO
4_chloro_3_methylphenol	-6.79	-5.260	C_7H_7OCl
4_chloroaniline	-5.90	-6.388	C_6H_6NCl
4_chlorophenol	-7.03	-5.238	C_6H_5OCl
4_cyanophenol	-10.17	-6.688	C_7H_5NO
4_cyanopyridine	-6.02	-4.660	$C_6H_4N_2$
4_ethylphenol	-6.13	-5.449	$C_8H_{10}O$
4_ethyltoluene	-0.95	-2.683	C_9H_{12}
4_fluorophenol	-6.19	-4.939	C_6H_5OF
4_formylpyridine	-7.00	-6.456	C_6H_5NO
4_isopropyltoluene	-0.68	-2.658	$C_{10}H_{14}$
4_methoxyacetophenone	-4.40	-6.086	$C_9H_{10}O_2$
4_methoxyaniline	-7.48	-7.552	C_7H_9NO
4_methyl_1h_imidazole	-10.27	-8.046	$C_4H_6N_2$

4_methylacetophenone	-4.70	-4.988	$C_9H_{10}O$
4_methylbenzaldehyde	-4.27	-5.282	C_8H_8O
4_methylpentan_2_ol	-3.73	-3.264	$C_6H_{14}O$
4_methylpentan_2_one	-3.05	-2.979	$C_6H_{12}O$
4_nitrophenol	-5.90	-9.146	$C_6H_5NO_3$
4_n_propylphenol	-10.64	-5.210	$C_9H_{12}O$
4_tert_butylphenol	-5.91	-5.428	$C_{10}H_{14}O$
acenaphthene	-3.15	-5.234	$C_{12}H_{10}$

Table S9. ESE-PM7 solvation free energies in water in kcal/mol. Blind[5] (63)

Solute	ΔG^o_{solv}(exp)	ΔG^o_{solv}(calc)	Formula
cup08001	-5.73	-3.597	$C_2H_4N_2O_6$
cup08002	-4.95	-5.188	$C_3H_6N_2O_6$
cup08003	-2.09	-1.507	$C_4H_9NO_3$
cup08004	-1.82	-1.684	$C_4H_9NO_3$
cup08005	-1.88	-1.420	$C_4H_9NO_3$
cup08006	-8.18	-5.975	$C_2H_5NO_4$
cup08007	-8.21	-7.892	$C_{14}H_{20}NO_2Cl$
cup08008	-9.84	-7.490	$C_7H_{14}N_2O_2S$
cup08009	-7.65	-10.357	$C_9H_{17}N_5S$
cup08010	-10.03	-10.000	$C_{10}H_{12}N_3O_3PS_2$
cup08011	-3.51	-9.473	$C_{13}H_{16}N_3O_4F_3$
cup08012	-17.17	-23.556	$C_{16}H_{18}N_4O_7S$
cup08013	-9.73	-9.733	$C_9H_{13}N_2O_2Br$
cup08014	-9.01	-4.625	$C_9H_8NO_2SCl_3$
cup08015	-9.45	-8.807	$C_{12}H_{11}NO_2$
cup08016	-9.61	-9.292	$C_{12}H_{15}NO_3$
cup08017	-6.50	-8.192	$C_{11}H_{16}O_2PS_3Cl$
cup08018	-3.44	-4.436	$C_{10}H_6Cl_8$
cup08019	-7.07	-2.148	$C_{12}H_{14}O_4PCl_3$
cup08020	-14.01	-15.942	$C_{15}H_{15}N_4O_6SCl$
cup08021	-1.45	-4.056	CNO_2Cl_3
cup08022	-5.04	-0.817	$C_9H_{11}NO_3PSCl_3$
cup08023	-5.74	-7.437	$C_{14}H_{17}NO_4PS_2Cl$
cup08024	-6.48	-4.012	$C_{12}H_{21}N_2O_3PS$
cup08025	-9.86	-6.856	$C_8H_6O_3Cl_2$
cup08026	-4.71	-3.190	$C_7H_3NCl_2$
cup08027	-5.66	-12.767	$C_{11}H_{13}N_4O_4F_3$
cup08028	-6.23	-12.269	$C_{10}H_{12}N_2O_5$
cup08029	-4.23	-12.877	$C_9H_6O_3SCl_6$
cup08030	-4.82	-6.309	$C_{12}H_8OCl_6$
cup08031	-6.10	-8.491	$C_9H_{22}O_4P_2S_4$
cup08032	-9.13	-9.362	$C_9H_{12}N_2O$
cup08033	-2.55	-4.195	$C_{10}H_5Cl_7$
cup08034	-5.18	-4.134	$C_9H_{14}O$
cup08035	-5.44	-3.764	$C_6H_6Cl_6$
cup08036	-8.15	-11.638	$C_{10}H_{19}O_6PS_2$
cup08037	-10.65	-7.439	$C_5H_{10}N_2O_2S$
cup08038	-7.19	-5.383	$C_8H_{10}NO_5PS$
cup08039	-15.54	-21.516	$C_{14}H_{15}N_5O_6S$
cup08040	-7.98	-20.016	$C_{13}H_{19}N_3O_6S$
cup08041	-5.99	-4.189	$C_3H_5NO_4$
cup08042	-10.18	-10.139	$C_7H_{13}N_3O_3S$
cup08043	-6.74	-6.713	$C_{10}H_{14}NO_5PS$

cup08044	-3.64	-4.489	$C_{10}H_{21}NOS$
cup08045	-4.37	-3.704	$C_7H_{17}O_2PS_3$
cup08046	-2.45	-4.952	$C_{14}H_{16}N_3O_4F_3$
cup08047	-8.43	-9.713	$C_{10}H_{19}N_5S$
cup08048	-7.78	-7.231	$C_9H_9NOCl_2$
cup08049	-16.43	-12.179	$C_{10}H_8N_3OCl$
cup08050	-10.22	-12.241	$C_7H_{12}N_5Cl$
cup08051	-20.25	-18.488	$C_{15}H_{16}N_4O_5S$
cup08052	-11.14	-8.249	$C_9H_{13}N_2O_2Cl$
cup08053	-6.68	-9.946	$C_{10}H_{19}N_5S$
cup08054	-16.23	-17.176	$C_{12}H_{13}N_5O_6S_2$
cup08055	-12.74	-9.632	$C_4H_8O_4PCl_3$
cup08056	-3.25	-6.296	$C_{13}H_{16}N_3O_4F_3$
cup08057	-4.13	-4.149	$C_{10}H_{21}NOS$
cup08058	-11.24	-13.544	$C_{12}H_{10}N_4O_2$
cup08059	-7.44	-10.373	$C_{20}H_{14}N_2O_2$
cup08060	-8.94	-18.208	$C_{14}H_{12}N_4O_2$
cup08061	-7.97	-7.369	$C_{14}H_9NO_2$
cup08062	-6.66	-9.932	$C_{14}H_{15}N_3$
cup08063	-9.41	-10.375	$C_{11}H_{18}N_4O_2$

Table S10. ESE-PM7 solvation free energies in water in kcal/mol. SAMPL1[3,5] (53)

**Solute**	**ΔG^{o}_{solv}(exp)**	**ΔG^{o}_{solv}(calc)**	**Formula**
1_2_Dinitroxypropane	-4.95	-5.223	$C_3H_6N_2O_6$
1_Aminoanthraquinone	-7.97	-7.381	$C_{14}H_9NO_2$
2_Butylnitrate	-1.82	-1.461	$C_4H_9NO_3$
4_Amino_4_nitroazobenzene	-11.24	-13.328	$C_{12}H_{10}N_4O_2$
4_Dimethylaminoazobenzene	-6.66	-9.037	$C_{14}H_{15}N_3$
Alachlor	-8.21	-8.086	$C_{14}H_{20}NO_2Cl$
Aldicarb	-9.84	-6.958	$C_7H_{14}N_2O_2S$
Ametryn	-7.65	-10.303	$C_9H_{17}N_5S$
Azinphos_methyl	-10.03	-7.815	$C_{10}H_{12}N_3O_3PS_2$
Benefin	-3.51	-9.468	$C_{13}H_{16}N_3O_4F_3$
Bromacil	-9.73	-9.818	$C_9H_{13}N_2O_2Br$
Butylnitrate	-2.09	-1.516	$C_4H_9NO_3$
Captan	-9.01	-3.597	$C_9H_8NO_2SCl_3$
Carbaryl	-9.45	-8.543	$C_{12}H_{11}NO_2$
Carbofuran	-9.61	-10.205	$C_{12}H_{15}NO_3$
Carbophenothion	-6.50	-8.920	$C_{11}H_{16}O_2PS_3Cl$
Chlordane	-3.44	-4.435	$C_{10}H_6Cl_8$
Chlorfenvinphos	-7.07	-3.036	$C_{12}H_{14}O_4PCl_3$
Chloropicrin	-1.45	-4.005	CNO_2Cl_3
Chlorpyrifos	-5.04	-1.474	$C_9H_{11}NO_3PSCl_3$
Diazinon	-6.48	-3.453	$C_{12}H_{21}N_2O_3PS$
Dicamba	-9.86	-7.097	$C_8H_6O_3Cl_2$
Dichlobenil	-4.71	-3.165	$C_7H_3NCl_2$
Dinitramine	-5.66	-16.277	$C_{11}H_{13}N_4O_4F_3$
Endrin	-4.82	-5.966	$C_{12}H_8OCl_6$
Ethion	-6.10	-8.079	$C_9H_{22}O_4P_2S_4$
Ethylenglycol_mononitrate	-8.18	-6.675	$C_2H_5NO_4$
Fenuron	-9.13	-9.226	$C_9H_{12}N_2O$
Heptachlor	-2.55	-4.258	$C_{10}H_5Cl_7$
Isobutylnitrate	-1.88	-1.422	$C_4H_9NO_3$
Isophorone	-5.18	-4.132	$C_9H_{14}O$

Lindane	-5.44	-3.266	$C_6H_6Cl_6$
Malathion	-8.15	-9.125	$C_{10}H_{19}O_6PS_2$
Methomyl	-10.65	-7.861	$C_5H_{10}N_2O_2S$
Methyl_parathion	-7.19	-5.378	$C_8H_{10}NO_5PS$
Nitralin	-7.98	-23.103	$C_{13}H_{19}N_3O_6S$
Nitroglycol	-5.73	-5.043	$C_2H_4N_2O_6$
Nitroxyacetone	-5.99	-5.864	$C_3H_5NO_4$
Parathion	-6.74	-3.053	$C_{10}H_{14}NO_5PS$
Pebulate	-3.64	-4.186	$C_{10}H_{21}NOS$
Phorate	-4.37	-4.391	$C_7H_{17}O_2PS_3$
Pirimor	-9.41	-11.122	$C_{11}H_{18}N_4O_2$
Profluralin	-2.45	-4.681	$C_{14}H_{16}N_3O_4F_3$
Prometryn	-8.43	-9.514	$C_{10}H_{19}N_5S$
Propanil	-7.78	-7.224	$C_9H_9NOCl_2$
Pyrazon	-16.43	-12.456	$C_{10}H_8N_3OCl$
Simazine	-10.22	-12.670	$C_7H_{12}N_5Cl$
Sulfometuron_methyl	-20.25	-18.971	$C_{15}H_{16}N_4O_5S$
Terbacil	-11.14	-8.089	$C_9H_{13}N_2O_2Cl$
Terbutryn	-6.68	-9.957	$C_{10}H_{19}N_5S$
Trichlorfon	-12.74	-8.272	$C_6H_{12}O_4PCl_3$
Trifluralin	-3.25	-8.978	$C_{13}H_{16}N_3O_4F_3$
Vernolate	-4.13	-4.102	$C_{10}H_{21}NOS$

Table S11. ESE-PM7 solvation free energies in water in kcal/mol. SAMPL4[5,6] (42)

Solute	ΔG^o_{solv}(exp)	ΔG^o_{solv}(calc)	Formula
1_1_Diphenylethene	-2.78	-4.684	$C_{14}H_{12}$
1_2_Dimethoxybenzene	-5.33	-6.044	$C_8H_{10}O_2$
1_2_Hydroxyethylamino_9_10_anthraquinone	-14.21	-10.739	$C_{16}H_{13}NO_3$
1_3_Bis_nitrooxybutane	-4.29	-4.574	$C_4H_8N_2O_6$
1_3_Bis_nitrooxypropane	-4.80	-4.943	$C_3H_6N_2O_6$
1_4_Diamino_9_10_anthraquinone	-11.85	-11.767	$C_{14}H_{10}N_2O_2$
1_Amino_4_hydroxy_9_10_anthraquinone	-9.53	-8.681	$C_{14}H_9NO_3$
1_Benzylimidazole	-7.63	-10.030	$C_{10}H_{10}N_2$
1_Butoxy_2_propanol	-5.73	-6.450	$C_7H_{16}O_2$
1_Ethyl_2_methylbenzene	-0.85	-2.885	C_9H_{12}
2_6_Dichlorosyringaldehyde	-8.68	-6.735	$C_9H_8O_4Cl_2$
2_6_Dimethoxyphenol	-6.96	-7.774	$C_8H_{10}O_3$
2_Amino_9_10_anthraquinone	-11.53	-8.289	$C_{14}H_9NO_2$
2_Chlorosyringaldehyde	-7.78	-6.284	$C_9H_9O_4Cl$
2_Ethoxyethyl_acetate	-5.31	-6.611	$C_6H_{12}O_3$
2_Ethylphenol	-5.66	-5.561	$C_8H_{10}O$
2_Methoxyphenol	-5.94	-5.985	$C_7H_8O_2$
2_Methylbenzaldehyde	-3.93	-5.209	C_8H_8O
3_4_Dichlorophenol	-7.29	-5.354	$C_6H_4OCl_2$
3_5_Dichlorosyringol	-6.44	-4.658	$C_8H_8O_3Cl_2$
3_Chlorosyringol	-6.86	-5.054	$C_8H_9O_3Cl$
4_5_Dichloroguaiacol	-3.58	-5.033	$C_7H_6O_2Cl_2$
4_Methyl_2_methoxyphenol	-5.80	-7.756	$C_8H_{10}O_2$
4_Propylguaiacol	-5.26	-5.293	$C_{10}H_{14}O_2$
9_10_Dihydroanthracene	-3.76	-6.372	$C_{14}H_{12}$
Amitriptyline	-7.43	-6.589	$C_{20}H_{23}N$
Carveol	-4.44	-5.429	$C_{10}H_{16}O$
Cyklohexene	0.14	-1.046	C_6H_{10}
Dibenzo_p_dioxin	-3.16	-2.473	$C_{12}H_8O_2$
Dihydrocarvone	-3.75	-3.679	$C_{10}H_{16}O$
Diphenhydramine	-9.34	-8.392	$C_{17}H_{21}NO$
Diphenylether	-2.32	-3.762	$C_{12}H_{10}O$
E_3_Hexenylacetate	-2.61	-6.106	$C_8H_{14}O_2$
Geraniol	-4.97	-5.893	$C_{10}H_{18}O$
Hexylacetate	-2.29	-4.119	$C_8H_{16}O_2$

Hexylnitrate	-1.66	-1.358	$C_6H_{13}NO_3$
Linalyl_acetate	-2.49	-5.533	$C_{12}H_{20}O_2$
Menthol	-3.20	-3.187	$C_{10}H_{20}O$
Menthone	-2.53	-3.055	$C_{10}H_{18}O$
Nerol	-3.96	-5.785	$C_{10}H_{18}O$
Piperitone	-4.51	-3.785	$C_{10}H_{16}O$
l_Perillaldehyde	-4.09	-4.655	$C_{10}H_{14}O$

Table S12. ESE-PM7 solvation free energies in water in kcal/mol. C10[5] (10)

Solute	ΔG^{o}_{solv}(exp)	ΔG^{o}_{solv}(calc)	Formula
23dimethylpyridine_plus	-56.21	-55.312	$C_7H_{10}N^+$
3ethylpyridine_plus	-57.38	-56.861	$C_7H_{10}N^+$
4methylpyridine_plus	-58.32	-58.530	$C_6H_8N^+$
butanoate	-74.75	-76.453	$C_4H_7O_2^-$
butylamine_plus	-71.19	-69.698	$C_4H_{12}N^+$
dibutylamine_plus	-59.31	-55.943	$C_8H_{20}N^+$
elthylamine_plus	-73.36	-74.728	$C_2H_8N^+$
pentanoate	-75.25	-74.975	$C_5H_9O_2^-$
propanol_minus	-88.25	-86.862	$C_3H_7O^-$
trifluoropropan2ol_minus	-77.74	-72.505	$C_3H_4OF_3^-$

Part 4. Solvation free energies in non-aqueous solvents. ESE-PM7 calculations versus experimental values.

Table S13. ESE-PM7 solvation free energies in non-aqueous polar protic solvents in kcal/mol.

Solvent	Solute	ΔG^{o}_{solv}(exp)[1,2]	ΔG^{o}_{solv}(calc)	Formula
octanol	0001met	0.51	0.189	CH_4
octanol	0002eth	-0.64	-0.573	C_2H_6
octanol	0003pro	-1.26	-1.192	C_3H_8
octanol	0004nbu	-1.86	-1.817	C_4H_{10}
octanol	0005npe	-2.45	-2.433	C_5H_{12}
octanol	0006nhe	-3.01	-3.040	C_6H_{14}
octanol	0007nhe	-3.74	-3.669	C_7H_{16}
octanol	0008noc	-4.18	-4.292	C_8H_{18}
octanol	0010met	-1.45	-1.787	C_4H_{10}
octanol	0011dim	-1.74	-2.381	C_5H_{12}
octanol	0016cyc	-1.60	-1.555	C_3H_6
octanol	0017cyc	-2.65	-2.978	C_5H_{10}
octanol	0018cyc	-3.46	-3.312	C_6H_{12}
octanol	0019met	-3.21	-3.808	C_7H_{14}
octanol	0021eth	-0.27	-0.449	C_2H_4
octanol	0022pro	-1.14	-1.552	C_3H_6
octanol	0023str	-2.10	-2.253	C_4H_6
octanol	0024met	-2.03	-2.553	C_4H_8
octanol	0025buta	-1.89	-2.338	C_4H_8
octanol	0029hex	-2.94	-3.245	C_6H_{12}
octanol	0030eth	-0.51	-0.251	C_2H_2
octanol	0031pro	-1.59	-2.126	C_3H_4
octanol	0033pen	-2.79	-3.122	C_5H_8
octanol	0034hex	-3.43	-3.482	C_6H_{10}
octanol	0035ben	-3.72	-4.716	C_6H_6
octanol	0036tol	-4.55	-5.524	C_7H_8
octanol	0037eth	-5.08	-6.068	C_8H_{10}
octanol	0038oxy	-5.07	-6.489	C_8H_{10}
octanol	0039mxy	-5.25	-6.346	C_8H_{10}
octanol	0040pxy	-5.19	-6.312	C_8H_{10}

octanol	0041nap	-6.97	-7.966	$C_{10}H_8$
octanol	0042ant	-10.47	-10.680	$C_{14}H_{10}$
octanol	0044met	-3.87	-3.719	CH_4O
octanol	0045eth	-4.36	-4.704	C_2H_6O
octanol	0046eth	-7.44	-7.834	$C_2H_6O_2$
octanol	0047pro	-5.02	-4.805	C_3H_8O
octanol	0048pro	-4.62	-4.564	C_3H_8O
octanol	0049but	-5.71	-5.562	$C_4H_{10}O$
octanol	0050met	-4.78	-5.442	$C_4H_{10}O$
octanol	0052pen	-6.40	-5.905	$C_5H_{12}O$
octanol	0053phe	-8.69	-7.730	C_6H_6O
octanol	0054hex	-7.06	-6.550	$C_6H_{14}O$
octanol	0055ocr	-8.49	-8.396	C_7H_8O
octanol	0056mcr	-8.20	-8.553	C_7H_8O
octanol	0057pcr	-8.84	-8.371	C_7H_8O
octanol	0058hep	-7.75	-7.150	$C_7H_{16}O$
octanol	0059dec	-9.88	-9.191	$C_{10}H_{22}O$
octanol	0060dim	-2.06	-2.237	C_2H_6O
octanol	0061tet	-3.93	-4.270	C_4H_8O
octanol	0062dio	-4.89	-5.214	$C_4H_8O_2$
octanol	0063die	-2.89	-3.600	$C_4H_{10}O$
octanol	0064met	-3.63	-3.619	$C_4H_{10}O$
octanol	0065met	-4.64	-3.943	$C_4H_{10}O$
octanol	0066dim	-4.55	-4.414	$C_4H_{10}O_2$
octanol	0067but	-3.49	-4.493	$C_5H_{12}O$
octanol	0068ani	-5.47	-6.160	C_7H_8O
octanol	0069met	-3.23	-2.579	CH_2O
octanol	0071proa	-4.13	-3.934	C_3H_6O
octanol	0072but	-4.62	-4.170	C_4H_8O
octanol	0074ben	-6.13	-6.466	C_7H_6O
octanol	0075pro	-3.15	-3.717	C_3H_6O
octanol	0076but	-3.78	-4.239	C_4H_8O
octanol	0077cyc	-5.01	-4.635	C_5H_8O
octanol	0078pen	-4.35	-4.558	$C_5H_{10}O$
octanol	0079pen	-4.36	-4.953	$C_5H_{10}O$
octanol	0080hex	-5.02	-5.170	$C_6H_{12}O$
octanol	0081dim	-4.53	-5.077	$C_6H_{12}O$
octanol	0082hep	-5.65	-5.779	$C_7H_{14}O$
octanol	0084met	-6.74	-6.863	C_8H_8O
octanol	0086eth	-6.35	-6.545	$C_2H_4O_2$
octanol	0087pro	-6.86	-6.551	$C_3H_6O_2$
octanol	0088but	-7.58	-6.808	$C_4H_8O_2$
octanol	0089pen	-8.22	-7.467	$C_5H_{10}O_2$
octanol	0090hex	-8.82	-8.097	$C_6H_{12}O_2$
octanol	0091met	-2.82	-3.988	$C_2H_4O_2$
octanol	0093met	-3.54	-4.100	$C_3H_6O_2$
octanol	0094met	-4.06	-4.291	$C_4H_8O_2$
octanol	0095eth	-4.06	-4.730	$C_4H_8O_2$
octanol	0096met	-4.59	-4.568	$C_5H_{10}O_2$
octanol	0097pro	-4.55	-4.810	$C_5H_{10}O_2$
octanol	0098met	-5.13	-5.142	$C_6H_{12}O_2$
octanol	0099but	-4.96	-5.516	$C_6H_{12}O_2$
octanol	0103eth	-4.09	-3.154	C_2H_7N
octanol	0106pro	-4.77	-3.721	C_3H_9N
octanol	0107tri	-3.60	-2.492	C_3H_9N
octanol	0109pip	-5.80	-6.001	$C_4H_{10}N_2$
octanol	0110but	-5.33	-4.474	$C_4H_{11}N$
octanol	0111die	-4.75	-3.654	$C_4H_{11}N$

octanol	0115dip	-6.02	-4.851	$C_6H_{15}N$
octanol	0116pyr	-5.34	-5.654	C_5H_5N
octanol	0117met	-5.87	-7.197	$C_5H_6N_2$
octanol	0118ani	-6.71	-8.842	C_6H_7N
octanol	0119met	-6.14	-6.290	C_6H_7N
octanol	0120met	-6.40	-6.442	C_6H_7N
octanol	0121met	-6.60	-6.558	C_6H_7N
octanol	0122Nme	-6.94	-8.605	C_7H_9N
octanol	0126eth	-3.15	-3.601	C_2H_3N
octanol	0127pro	-3.66	-3.729	C_3H_5N
octanol	0128butb	-4.25	-4.251	C_4H_7N
octanol	0129ben	-6.09	-5.312	C_7H_5N
octanol	0130nit	-3.93	-6.799	$C_2H_5NO_2$
octanol	0131nit	-4.44	-6.940	$C_3H_7NO_2$
octanol	0132nit	-4.23	-6.411	$C_3H_7NO_2$
octanol	0133nit	-5.11	-7.520	$C_4H_9NO_2$
octanol	0134nit	-6.63	-7.229	$C_6H_5NO_2$
octanol	0135met	-6.80	-7.521	$C_7H_7NO_2$
octanol	0138pro	-3.52	-2.962	C_3H_8S
octanol	0139thi	-5.99	-5.696	C_6H_6S
octanol	0141dim	-4.24	-3.577	$C_2H_6S_2$
octanol	0142die	-4.09	-4.982	$C_4H_{10}S$
octanol	0144thi	-6.47	-7.207	C_7H_8S
octanol	0145pro	-5.27	-4.629	C_3H_6O
octanol	0146met	-5.83	-6.268	$C_3H_8O_2$
octanol	0149mor	-5.99	-5.774	C_4H_9NO
octanol	0150mhy	-11.39	-9.588	$C_7H_6O_2$
octanol	0151phy	-12.36	-10.076	$C_7H_6O_2$
octanol	0152eth	-6.85	-7.808	$C_7H_{10}N_2O$
octanol	0154dif	-1.13	-2.502	$C_2H_4F_2$
octanol	0157flu	-3.87	-3.357	C_6H_5F
octanol	0161dic	-3.07	-1.971	CH_2Cl_2
octanol	0162tri	-3.81	-3.678	$CHCl_3$
octanol	0163chl	-2.58	-1.212	C_2H_5Cl
octanol	0165tri	-3.69	-3.466	$C_2H_3Cl_3$
octanol	0166tri	-4.53	-3.431	$C_2H_3Cl_3$
octanol	0167chla	-3.06	-1.431	C_3H_7Cl
octanol	0168chl	-2.84	-1.806	C_3H_7Cl
octanol	0171Zdi	-3.71	-2.773	$C_2H_2Cl_2$
octanol	0172Edi	-3.61	-2.015	$C_2H_2Cl_2$
octanol	0173tri	-3.75	-4.054	C_2HCl_3
octanol	0174chl	-5.00	-4.593	C_6H_5Cl
octanol	0175odi	-6.01	-5.536	$C_6H_4Cl_2$
octanol	0176pdi	-5.67	-4.714	$C_6H_4Cl_2$
octanol	0177bro	-2.43	-1.345	CH_3Br
octanol	0178dib	-4.18	-4.341	CH_2Br_2
octanol	0179tri	-5.62	-8.096	$CHBr_3$
octanol	0180bro	-2.90	-1.650	C_2H_5Br
octanol	0182bro	-3.42	-2.073	C_3H_7Br
octanol	0183bro	-3.40	-2.017	C_3H_7Br
octanol	0184bro	-4.16	-2.686	C_4H_9Br
octanol	0185bro	-4.68	-3.194	$C_5H_{11}Br$
octanol	0186bro	-5.46	-6.016	C_6H_5Br
octanol	0187dib	-7.47	-7.945	$C_6H_4Br_2$
octanol	0197bro	-0.75	-3.669	CF_3Br
octanol	0199chl	-1.97	-2.782	CHF_2Cl
octanol	0200tet	1.50	2.000	CF_4
octanol	0201bro	-3.27	-5.290	C_2HF_3ClBr

octanol	0204tet	-4.24	-5.982	C_2Cl_4
octanol	0206tri	-2.54	-4.479	$C_2F_3Cl_3$
octanol	0207tri	-4.81	-5.868	$C_2H_3OF_3$
octanol	0210dic	-4.02	-5.118	$C_3H_4OF_2Cl_2$
octanol	0211tri	-5.12	-5.551	$C_3H_5OF_3$
octanol	0212hex	-5.76	-5.908	$C_3H_2OF_6$
octanol	0215pbr	-10.59	-9.219	C_6H_5OBr
octanol	0217wat	-4.43	-6.612	H_2O
octanol	0220tri	-7.81	-6.414	$C_3H_9O_4P$
octanol	0221tri	-8.88	-8.249	$C_6H_{15}O_4P$
octanol	0222tri	-8.65	-8.784	$C_9H_{21}O_4P$
octanol	0225pipa	-6.27	-4.502	$C_5H_{11}N$
octanol	0228met	-3.78	-2.744	CH_5N
octanol	0229hyd	-3.44	-5.436	H_4N_2
octanol	0230eth	-6.40	-7.609	$C_6H_8N_2$
octanol	0236oct	-8.13	-7.794	$C_8H_{18}O$
octanol	0239oct	-6.38	-6.392	$C_8H_{16}O$
octanol	0240met	-7.26	-5.947	$C_8H_8O_2$
octanol	0244tet	-4.21	-4.451	$C_5H_{10}O$
octanol	0245thi	-3.89	-3.095	C_4H_4S
octanol	0246eth	-5.65	-6.827	$C_8H_{10}O$
octanol	0400hyd	1.76	0.164	H_2
octanol	0401amia	-13.12	-12.013	$C_9H_{12}N_2O$
octanol	0402adn	-13.56	-17.866	$C_6H_7N_5$
octanol	0414dcl	-9.41	-9.338	$C_{12}H_8Cl_2$
octanol	0415dcl	-9.23	-9.397	$C_{12}H_8Cl_2$
octanol	0416dcl	-9.12	-9.970	$C_{12}H_7Cl_3$
octanol	0417brp	-3.30	-2.672	C_3H_5Br
octanol	0419brt	-6.36	-6.744	C_7H_7Br
octanol	0421dfl	-1.25	-2.423	CF_2Cl_2
octanol	0422ftc	-2.63	-3.981	$CFCl_3$
octanol	0426dcl	-9.18	-6.986	$C_7H_3NCl_2$
octanol	0428ami	-12.37	-18.016	$C_6H_3N_2O_2Cl_3$
octanol	0433pho	-8.59	-7.502	$C_4H_7O_4PCl_2$
octanol	0437pho	-12.55	-14.163	$C_9H_{13}O_3PS_2$
octanol	0438pho	-10.87	-10.098	$C_{10}H_{13}O_3PSCl_2$
octanol	0441pho	-11.70	-10.168	$C_8H_{10}NO_5PS$
octanol	0442pho	-10.49	-11.491	$C_{11}H_{15}O_3PSClBr$
octanol	0444pho	-11.69	-8.914	$C_8H_8O_3PSCl_3$
octanol	0445pho	-12.30	-10.481	$C_8H_8O_3PSCl_2Br$
octanol	0447pho	-11.31	-12.437	$C_{10}H_{14}NO_5PS$
octanol	0449pho	-11.06	-13.623	$C_{15}H_{14}NO_2PS$
octanol	0506nit	-3.51	-6.953	CH_3NO_2
octanol	0515dim	-6.14	-7.100	C_3H_7NO
octanol	0517met	-6.97	-7.577	C_2H_5NO
octanol	0519dim	-7.48	-6.911	C_4H_9NO
octanol	0574eth	-7.80	-6.993	C_7H_9N
octanol	0579pyy	-5.28	-6.783	C_4H_5N
octanol	0582qui	-8.43	-8.634	C_9H_7N
octanol	0648gbu	-6.83	-6.071	$C_4H_6O_2$
octanol	0939tet	-1.79	-4.008	$C_4H_{12}Si$
octanol	n005	-3.88	-4.770	CH_6N_2
octanol	n007	-10.93	-11.887	CH_4N_2O
octanol	n008	-11.77	-11.083	C_7H_7NO
octanol	n009	-7.36	-9.461	C_7H_9N
octanol	n010	-7.57	-9.737	C_7H_9N
octanol	n011	-7.44	-9.540	C_7H_9N
octanol	n127	-7.80	-8.666	CH_3NO

octanol	n185	-8.85	-8.211	C_4H_7NO
octanol	n186	-8.63	-7.420	C_5H_9NO
octanol	n191	-15.13	-12.847	$C_4H_4N_2O_2$
octanol	n200	-15.71	-11.784	$C_4H_3N_2O_2F$
octanol	n201	-15.51	-13.521	$C_5H_3N_2O_2F_3$
octanol	n202	-17.26	-13.736	$C_4H_3N_2O_2Cl$
octanol	n203	-17.88	-15.606	$C_4H_3N_2O_2Br$
octanol	test1035	-10.71	-8.710	$C_6H_6Cl_6$
octanol	test2001	-11.56	-11.206	$C_9H_8O_4$
octanol	test2003	-13.59	-10.892	$C_{11}H_{14}O_3$
octanol	test2004	-12.54	-13.462	$C_8H_{10}N_4O_2$
octanol	test2007	-18.89	-15.107	$C_3H_3N_3O_3$
octanol	test2010	-15.46	-14.290	$C_{13}H_8O_3F_2$
octanol	test2011	-12.57	-9.805	$C_9H_{10}O_3$
octanol	test2013	-14.10	-13.560	$C_{15}H_{13}O_2F$
octanol	test2015	-10.15	-10.328	C_6Cl_6
octanol	test2017	-12.42	-12.916	$C_{13}H_{18}O_2$
octanol	test2018	-18.77	-16.915	$C_4H_3N_2O_2I$
octanol	test2019	-15.04	-14.990	$C_{16}H_{14}O_3$
octanol	test2020	-12.18	-9.444	$C_8H_8O_3$
octanol	test2021	-14.55	-15.070	$C_{14}H_{14}O_3$
octanol	test2022	-11.35	-12.724	$C_6H_6N_2O_2$
octanol	test2023	0.31	2.408	C_4F_8
octanol	test2024	-11.55	-12.486	$C_6NO_2Cl_5$
octanol	test2025	-11.18	-7.902	$C_8H_5NO_2$
octanol	test2026	-13.52	-10.093	$C_{10}H_{12}O_3$
octanol	test2027	-7.56	-10.431	$C_4H_8O_2S$
octanol	test3001	-15.46	-13.531	$C_8H_9NO_2$
octanol	test3002	-14.93	-13.677	$C_8H_9NO_2$
octanol	test3003	-17.12	-19.172	$C_{16}H_{14}O_3$
octanol	test3004	-12.59	-12.511	$C_8H_9NO_2$
octanol	test3005	-13.07	-12.572	$C_{10}H_{13}NO_2$
octanol	test3007	-12.49	-10.661	$C_8H_8O_3$
octanol	test3014	-11.82	-9.443	$C_8H_8O_3$
octanol	test3015	-11.69	-9.093	$C_8H_8O_3$
octanol	test3019	-13.76	-14.138	$C_{14}H_{12}NO_2Cl$
octanol	test3020	-12.45	-18.460	$C_{14}H_{11}NO_2Cl_2$
octanol	test3021	-12.84	-15.201	$C_{14}H_{10}NO_2F_3$
octanol	test4001	-6.18	-7.702	C_6H_5I
octanol	test4002	-5.63	-6.629	CH_2I_2
octanol	test4003	-3.07	-2.672	CH_3I
octanol	test4004	-3.45	-3.099	C_2H_5I
octanol	test4009	-4.40	-3.536	C_3H_7I
heptanol	0035ben	-3.73	-4.800	C_6H_6
heptanol	0036tol	-4.33	-5.616	C_7H_8
heptanol	0037eth	-4.58	-6.168	C_8H_{10}
heptanol	0053phe	-8.69	-7.858	C_6H_6O
heptanol	0055ocr	-8.78	-8.532	C_7H_8O
heptanol	0057pcr	-9.16	-8.507	C_7H_8O
heptanol	0058hep	-7.84	-7.277	$C_7H_{16}O$
heptanol	0086eth	-6.70	-6.693	$C_2H_4O_2$
heptanol	0103eth	-4.15	-3.194	C_2H_7N
heptanol	0106pro	-4.80	-3.769	C_3H_9N
heptanol	0110but	-5.40	-4.529	$C_4H_{11}N$
heptanol	0215pbr	-10.49	-9.348	C_6H_5OBr
m-cresol	0008noc	-4.02	-4.393	C_8H_{18}
m-cresol	0036tol	-4.58	-5.672	C_7H_8
m-cresol	0045eth	-5.58	-4.872	C_2H_6O

m-cresol	0056mcr	-8.40	-8.773	C_7H_8O
m-cresol	0062dio	-6.82	-5.441	$C_4H_8O_2$
m-cresol	0076but	-5.98	-4.418	C_4H_8O
m-cresol	0506nit	-4.73	-7.173	CH_3NO_2
benzyl alcohol	0008noc	-3.77	-4.393	C_8H_{18}
benzyl alcohol	0036tol	-4.46	-5.672	C_7H_8
benzyl alcohol	0045eth	-4.84	-4.873	C_2H_6O
benzyl alcohol	0062dio	-5.39	-5.442	$C_4H_8O_2$
benzyl alcohol	0069met	-3.48	-2.727	CH_2O
benzyl alcohol	0076but	-4.57	-4.419	C_4H_8O
benzyl alcohol	0086eth	-6.96	-6.784	$C_2H_4O_2$
benzyl alcohol	0110but	-5.15	-4.564	$C_4H_{11}N$
benzyl alcohol	0506nit	-4.53	-7.174	CH_3NO_2
benzyl alcohol	0533ben	-8.61	-9.118	C_7H_8O
hexanol	0035ben	-3.68	-4.855	C_6H_6
hexanol	0036tol	-4.27	-5.675	C_7H_8
hexanol	0037eth	-4.54	-6.232	C_8H_{10}
hexanol	0053phe	-8.76	-7.941	C_6H_6O
hexanol	0054hex	-7.05	-6.748	$C_6H_{14}O$
hexanol	0055ocr	-8.76	-8.619	C_7H_8O
hexanol	0056mcr	-8.42	-8.778	C_7H_8O
hexanol	0057pcr	-9.21	-8.594	C_7H_8O
hexanol	0069met	-3.42	-2.729	CH_2O
hexanol	0086eth	-6.51	-6.788	$C_2H_4O_2$
hexanol	0103eth	-4.20	-3.221	C_2H_7N
hexanol	0106pro	-4.83	-3.799	C_3H_9N
hexanol	0110but	-5.50	-4.565	$C_4H_{11}N$
hexanol	0215pbr	-10.51	-9.430	C_6H_5OBr
pentanol	0035ben	-3.53	-4.944	C_6H_6
pentanol	0036tol	-4.25	-5.772	C_7H_8
pentanol	0037eth	-4.48	-6.338	C_8H_{10}
pentanol	0052pen	-7.92	-6.211	$C_5H_{12}O$
pentanol	0053phe	-8.55	-8.077	C_6H_6O
pentanol	0055ocr	-8.57	-8.763	C_7H_8O
pentanol	0057pcr	-9.25	-8.739	C_7H_8O
pentanol	0069met	-3.44	-2.825	CH_2O
pentanol	0086eth	-6.65	-6.945	$C_2H_4O_2$
pentanol	0087pro	-7.09	-6.949	$C_3H_6O_2$
pentanol	0088but	-7.74	-7.210	$C_4H_8O_2$
pentanol	0089pen	-8.17	-7.888	$C_5H_{10}O_2$
pentanol	0090hex	-8.99	-8.538	$C_6H_{12}O_2$
pentanol	0103eth	-4.27	-3.264	C_2H_7N
pentanol	0106pro	-4.88	-3.849	C_3H_9N
pentanol	0110but	-5.55	-4.624	$C_4H_{11}N$
pentanol	0111die	-5.30	-3.811	$C_4H_{11}N$
pentanol	0118ani	-6.44	-9.129	C_6H_7N
pentanol	0215pbr	-10.62	-9.567	C_6H_5OBr
pentanol	0216amm	-3.13	-3.507	H_3N
pentanol	0228met	-3.95	-2.844	CH_5N
pentanol	n017	-7.46	-5.787	H_2O_2
sec-butanol	0069met	-2.86	-2.849	CH_2O
sec-butanol	0086eth	-6.81	-6.983	$C_2H_4O_2$
sec-butanol	0087pro	-7.00	-6.987	$C_3H_6O_2$
sec-butanol	0088but	-7.34	-7.248	$C_4H_8O_2$
sec-butanol	0089pen	-7.61	-7.928	$C_5H_{10}O_2$
sec-butanol	0090hex	-8.11	-8.580	$C_6H_{12}O_2$
sec-butanol	0110but	-5.52	-4.638	$C_4H_{11}N$
sec-butanol	0217wat	-5.71	-6.920	H_2O

sec-butanol	0509sec	-5.48	-5.784	$C_4H_{10}O$
isobutanol	0072but	-4.82	-4.525	C_4H_8O
isobutanol	0086eth	-6.80	-7.019	$C_2H_4O_2$
isobutanol	0087pro	-6.98	-7.022	$C_3H_6O_2$
isobutanol	0088but	-7.62	-7.284	$C_4H_8O_2$
isobutanol	0089pen	-8.06	-7.965	$C_5H_{10}O_2$
isobutanol	0090hex	-8.77	-8.618	$C_6H_{12}O_2$
isobutanol	0095eth	-4.27	-5.194	$C_4H_8O_2$
isobutanol	0104dim	-4.43	-2.618	C_2H_7N
isobutanol	0107tri	-3.90	-2.679	C_3H_9N
isobutanol	0109pip	-6.58	-6.274	$C_4H_{10}N_2$
isobutanol	0111die	-4.86	-3.840	$C_4H_{11}N$
isobutanol	0115dip	-5.87	-5.078	$C_6H_{15}N$
isobutanol	0116pyr	-5.87	-5.986	C_5H_5N
isobutanol	0225pipa	-6.17	-4.714	$C_5H_{11}N$
isobutanol	0228met	-4.56	-2.863	CH_5N
isobutanol	0507iso	-5.79	-5.661	$C_4H_{10}O$
isobutanol	n017	-7.93	-5.825	H_2O_2
methoxyethanol	0008noc	-3.71	-4.499	C_8H_{18}
methoxyethanol	0036tol	-4.49	-5.828	C_7H_8
methoxyethanol	0045eth	-4.71	-5.049	C_2H_6O
methoxyethanol	0062dio	-4.91	-5.681	$C_4H_8O_2$
methoxyethanol	0076but	-4.28	-4.607	C_4H_8O
methoxyethanol	0506nit	-5.06	-7.407	CH_3NO_2
butanol	0008noc	-4.45	-4.501	C_8H_{18}
butanol	0035ben	-3.39	-4.998	C_6H_6
butanol	0036tol	-4.50	-5.832	C_7H_8
butanol	0037eth	-4.46	-6.403	C_8H_{10}
butanol	0044met	-4.73	-4.019	CH_4O
butanol	0045eth	-5.02	-5.053	C_2H_6O
butanol	0046eth	-8.69	-8.386	$C_2H_6O_2$
butanol	0049but	-6.03	-5.934	$C_4H_{10}O$
butanol	0069met	-3.49	-2.884	CH_2O
butanol	0076but	-4.12	-4.610	C_4H_8O
butanol	0086eth	-6.81	-7.040	$C_2H_4O_2$
butanol	0087pro	-7.17	-7.044	$C_3H_6O_2$
butanol	0088but	-7.66	-7.306	$C_4H_8O_2$
butanol	0089pen	-8.02	-7.988	$C_5H_{10}O_2$
butanol	0090hex	-8.75	-8.642	$C_6H_{12}O_2$
butanol	0103eth	-4.50	-3.290	C_2H_7N
butanol	0106pro	-5.04	-3.880	C_3H_9N
butanol	0111die	-5.15	-3.848	$C_4H_{11}N$
butanol	0506nit	-3.93	-7.412	CH_3NO_2
butanol	n017	-7.94	-5.836	H_2O_2
butanol	n191	-16.05	-13.520	$C_4H_4N_2O_2$
isopropanol	0008noc	-4.50	-4.528	C_8H_{18}
isopropanol	0036tol	-4.38	-5.872	C_7H_8
isopropanol	0045eth	-4.84	-5.099	C_2H_6O
isopropanol	0048pro	-4.82	-4.936	C_3H_8O
isopropanol	0062dio	-4.49	-5.748	$C_4H_8O_2$
isopropanol	0076but	-4.07	-4.660	C_4H_8O
isopropanol	0506nit	-4.00	-7.472	CH_3NO_2
propanol	0008noc	-4.39	-4.544	C_8H_{18}
propanol	0036tol	-4.47	-5.895	C_7H_8
propanol	0045eth	-5.01	-5.124	C_2H_6O
propanol	0047pro	-5.29	-5.224	C_3H_8O
propanol	0062dio	-4.61	-5.782	$C_4H_8O_2$
propanol	0076but	-4.15	-4.687	C_4H_8O

propanol	0506nit	-4.04	-7.506	CH_3NO_2
ethanol	0008noc	-4.23	-4.584	C_8H_{18}
ethanol	0036tol	-4.57	-5.955	C_7H_8
ethanol	0045eth	-5.04	-5.192	C_2H_6O
ethanol	0062dio	-4.68	-5.874	$C_4H_8O_2$
ethanol	0076but	-4.32	-4.759	C_4H_8O
ethanol	0174chl	-3.30	-4.986	C_6H_5Cl
ethanol	0506nit	-4.34	-7.595	CH_3NO_2
ethanol	0648gbu	-4.58	-6.764	$C_4H_6O_2$
methanol	i059	-72.90	-72.861	$C_2H_3O_2^-$
methanol	i060	-72.00	-72.640	$C_3H_5O_2^-$
methanol	i062	-68.80	-70.144	$C_3H_3O_2^-$
methanol	i064	-67.50	-67.624	$C_7H_5O_2^-$
methanol	i074	-69.30	-67.503	$C_6H_5O^-$
methanol	i079	-70.00	-66.055	$C_7H_7O_2^-$
methanol	i101	-58.90	-55.605	$C_6H_4NO_3^-$
methanol	i102	-54.10	-54.869	$C_6H_4NO_3^-$
methanol	i115	-71.50	-77.058	Cl^-
methanol	i116	-65.80	-67.167	Br^-
methanol	i119	-64.70	-64.658	$C_2H_2O_2Cl^-$
methanol	i120	-59.30	-57.539	$C_2HO_2Cl_2^-$
methanol	i123	-64.20	-62.007	$C_6H_4OCl^-$
methanol	i191	-43.90	-49.915	$C_6H_3N_2O_5^-$
methanol	i194	-64.10	-65.799	$C_7H_4O_2Cl^-$
methanol	i197	-67.50	-65.735	$C_7H_4O_2F^-$
methanol	i198	-66.00	-63.488	$C_6H_4OF^-$
methanol	i199	-55.40	-58.857	$C_7H_5O_3^-$
methanol	i205	-62.20	-62.511	$C_7H_4NO_4^-$
methanol	i207	-61.60	-66.282	$C_{10}H_{13}O^-$
methanol	i209	-72.10	-69.439	$C_9H_9O_2^-$
methanol	i213	-63.90	-61.100	$C_7H_4O_2Cl^-$
methanol	i215	-64.00	-58.347	$C_6H_4OCl^-$
methanol	i217	-59.00	-59.927	$C_8H_4NO_2^-$
methanol	i218	-68.40	-67.801	$C_7H_5O_3^-$
methanol	i219	-68.20	-68.307	$C_8H_7O_2^-$
methanol	i220	-59.30	-59.064	$C_7H_4NO_4^-$
methanol	i221	-60.20	-57.896	$C_8H_4O_2F_3^-$
methanol	i224	-73.10	-70.739	$C_7H_6NO_2^-$
methanol	i226	-63.90	-61.110	$C_7H_4O_2Cl^-$
methanol	i228	-64.40	-62.636	$C_7H_4O_2F^-$
methanol	i229	-59.20	-60.433	$C_7H_5O_2^-$
methanol	i231	-65.70	-68.534	$C_7H_5O_3^-$
methanol	i232	-69.00	-68.717	$C_8H_7O_3^-$
methanol	i233	-68.60	-68.545	$C_8H_7O_2^-$
methanol	i234	-58.20	-58.651	$C_7H_4NO_4^-$
methanol	i235	-67.40	-70.346	$C_{11}H_{13}O_2^-$
methanol	i236	-68.00	-67.684	$C_{10}H_{13}O^-$
methanol	i242	-63.00	-63.933	$C_2H_2O_2Br^-$
methanol	i243	-71.40	-71.408	$C_4H_7O_2^-$
methanol	i245	-61.70	-62.335	$C_3H_2NO_2^-$
methanol	i246	-71.30	-72.022	$C_5H_7O_2^-$
methanol	i247	-70.80	-71.680	$C_7H_{11}O_2^-$
methanol	i250	-71.70	-71.217	$C_4H_5O_2^-$
methanol	i253	-67.20	-68.452	$C_2H_2O_2F^-$
methanol	i255	-66.60	-68.119	$C_2H_3O_3^-$
methanol	i259	-69.80	-67.853	$C_7H_7O^-$
methanol	i266	-68.10	-68.219	$C_7H_7O^-$
methanol	i267	-70.20	-67.671	$C_7H_7O^-$

methanol	i269	-69.50	-68.308	$C_8H_7O_2^-$
methanol	i281	-67.70	-68.011	$C_8H_7O_3^-$
methanol	i003	-74.30	-78.790	CH_6N^+
methanol	i013	-59.50	-61.847	$C_3H_{10}N^+$
methanol	i014	-53.00	-54.206	$C_6H_{16}N^+$
methanol	i018	-74.10	-70.448	$C_6H_8N^+$
methanol	i019	-72.10	-69.100	$C_7H_{10}N^+$
methanol	i020	-71.30	-68.917	$C_7H_{10}N^+$
methanol	i021	-71.70	-68.822	$C_7H_{10}N^+$
methanol	i024	-63.50	-62.819	$C_7H_{10}N^+$
methanol	i029	-69.60	-69.877	$C_{10}H_{10}N^+$
methanol	i033	-62.90	-62.809	$C_5H_{12}N^+$
methanol	i036	-60.80	-61.958	$C_5H_6N^+$
methanol	i037	-57.50	-56.688	$C_9H_8N^+$
methanol	i047	-85.60	-92.017	H_4N^+
methanol	i093	-72.10	-68.536	$C_7H_{10}NO^+$
methanol	i094	-75.30	-80.620	$C_6H_7N_2O_2^+$
methanol	i125	-76.00	-73.383	$C_6H_7NCl^+$
methanol	i126	-75.50	-73.367	$C_6H_7NCl^+$
methanol	i130	-56.30	-56.535	$C_6H_{14}N^+$
methanol	i133	-55.60	-54.669	$C_7H_{10}N^+$
methanol	i140	-57.70	-58.018	$C_6H_8N^+$
methanol	i141	-75.20	-75.309	$C_6H_7NBr^+$
methanol	i144	-68.00	-69.178	$C_7H_{10}NO^+$
methanol	i145	-58.50	-59.050	$C_6H_8N^+$
methanol	i148	-72.10	-65.474	$C_6H_5N_2^+$
methanol	i149	-57.60	-58.076	$C_7H_{10}N^+$
methanol	i151	-58.10	-58.902	$C_6H_8N^+$
methanol	i153	-62.30	-57.967	$C_{11}H_{10}N^+$
methanol	i163	-71.00	-74.182	$C_2H_8N^+$
methanol	i176	-70.80	-72.368	$C_4H_{12}N^+$

Table S14. ESE-PM7 solvation free energies in polar aprotic solvents in kcal/mol.

Solvent	Solute	ΔG^o_{solv}(exp)[1,2]	ΔG^o_{solv}(calc)	Formula
bromoethane	0008noc	-5.54	-5.258	C_8H_{18}
bromoethane	0036tol	-5.55	-5.642	C_7H_8
bromoethane	0062dio	-5.39	-5.388	$C_4H_8O_2$
bromoethane	0076but	-5.13	-4.244	C_4H_8O
bromoethane	0087pro	-5.52	-5.881	$C_3H_6O_2$
bromoethane	0180bro	-3.67	-2.634	C_2H_5Br
bromoethane	0506nit	-4.54	-6.926	CH_3NO_2
2-methylpyridine	0008noc	-4.73	-4.774	C_8H_{18}
2-methylpyridine	0036tol	-5.06	-5.337	C_7H_8
2-methylpyridine	0045eth	-5.01	-3.586	C_2H_6O
2-methylpyridine	0062dio	-5.01	-5.177	$C_4H_8O_2$
2-methylpyridine	0076but	-4.52	-4.007	C_4H_8O
2-methylpyridine	0119met	-5.71	-6.505	C_6H_7N
o-dichlorobenzene	0044met	-1.73	-2.317	CH_4O
o-dichlorobenzene	0045eth	-2.34	-3.335	C_2H_6O
o-dichlorobenzene	0047pro	-3.47	-3.408	C_3H_8O
o-dichlorobenzene	0049but	-3.90	-4.269	$C_4H_{10}O$
o-dichlorobenzene	0052pen	-4.93	-4.740	$C_5H_{12}O$
o-dichlorobenzene	0054hex	-5.70	-5.472	$C_6H_{14}O$
o-dichlorobenzene	0058hep	-6.50	-6.172	$C_7H_{16}O$
o-dichlorobenzene	0103eth	-2.59	-1.477	C_2H_7N
o-dichlorobenzene	0106pro	-3.44	-2.101	C_3H_9N
o-dichlorobenzene	0110but	-4.13	-2.961	$C_4H_{11}N$
o-dichlorobenzene	0175odi	-6.89	-9.331	$C_6H_4Cl_2$

dichloroethane	0044met	-2.53	-2.484	CH_4O
dichloroethane	0045eth	-2.83	-3.557	C_2H_6O
dichloroethane	0047pro	-3.85	-3.684	C_3H_8O
dichloroethane	0049but	-4.92	-4.596	$C_4H_{10}O$
dichloroethane	0052pen	-5.45	-5.122	$C_5H_{12}O$
dichloroethane	0053phe	-7.48	-7.103	C_6H_6O
dichloroethane	0054hex	-6.02	-5.908	$C_6H_{14}O$
dichloroethane	0055ocr	-7.73	-7.774	C_7H_8O
dichloroethane	0056mcr	-6.91	-7.968	C_7H_8O
dichloroethane	0057pcr	-7.75	-7.737	C_7H_8O
dichloroethane	0058hep	-6.79	-6.660	$C_7H_{16}O$
dichloroethane	0074ben	-7.23	-7.394	C_7H_6O
dichloroethane	0084met	-7.83	-7.650	C_8H_8O
dichloroethane	0086eth	-4.89	-5.657	$C_2H_4O_2$
dichloroethane	0087pro	-5.12	-5.665	$C_3H_6O_2$
dichloroethane	0088but	-5.83	-6.059	$C_4H_8O_2$
dichloroethane	0089pen	-6.47	-6.965	$C_5H_{10}O_2$
dichloroethane	0090hex	-7.33	-7.808	$C_6H_{12}O_2$
dichloroethane	0093met	-4.55	-4.019	$C_3H_6O_2$
dichloroethane	0094met	-4.87	-4.292	$C_4H_8O_2$
dichloroethane	0095eth	-4.93	-4.422	$C_4H_8O_2$
dichloroethane	0097pro	-5.40	-4.725	$C_5H_{10}O_2$
dichloroethane	0098met	-5.97	-5.502	$C_6H_{12}O_2$
dichloroethane	0099but	-5.93	-5.612	$C_6H_{12}O_2$
dichloroethane	0100met	-6.57	-6.320	$C_7H_{14}O_2$
dichloroethane	0101pen	-6.64	-6.403	$C_7H_{14}O_2$
dichloroethane	0103eth	-3.19	-1.724	C_2H_7N
dichloroethane	0106pro	-4.04	-2.404	C_3H_9N
dichloroethane	0110but	-4.34	-3.316	$C_4H_{11}N$
dichloroethane	0111die	-4.00	-3.421	$C_4H_{11}N$
dichloroethane	0116pyr	-5.53	-5.699	C_5H_5N
dichloroethane	0118ani	-7.39	-7.013	C_6H_7N
dichloroethane	0150mhy	-10.11	-10.270	$C_7H_6O_2$
dichloroethane	0151phy	-10.70	-10.644	$C_7H_6O_2$
dichloroethane	0215pbr	-9.10	-10.210	C_6H_5OBr
dichloroethane	0220tri	-8.55	-10.411	$C_3H_9O_4P$
dichloroethane	0221tri	-9.59	-11.852	$C_6H_{15}O_4P$
dichloroethane	0521dic	-4.69	-3.935	$C_2H_4Cl_2$
dichloroethane	n008	-10.90	-9.792	C_7H_7NO
4-methyl-2-pentanone	0041nap	-7.45	-9.416	$C_{10}H_8$
4-methyl-2-pentanone	0053phe	-9.38	-7.726	C_6H_6O
4-methyl-2-pentanone	0056mcr	-8.79	-8.673	C_7H_8O
4-methyl-2-pentanone	0086eth	-6.33	-6.184	$C_2H_4O_2$
4-methyl-2-pentanone	0087pro	-6.85	-6.252	$C_3H_6O_2$
4-methyl-2-pentanone	0088but	-7.44	-6.715	$C_4H_8O_2$
4-methyl-2-pentanone	0107tri	-2.86	-3.633	C_3H_9N
4-methyl-2-pentanone	0111die	-3.63	-3.936	$C_4H_{11}N$
4-methyl-2-pentanone	0116pyr	-5.33	-6.255	C_5H_5N
4-methyl-2-pentanone	0118ani	-7.54	-7.643	C_6H_7N
4-methyl-2-pentanone	0216amm	-2.52	-0.804	H_3N
4-methyl-2-pentanone	0228met	-4.14	-1.566	CH_5N
4-methyl-2-pentanone	0508met	-5.23	-5.892	$C_6H_{12}O$
pyridine	0008noc	-4.50	-4.486	C_8H_{18}
pyridine	0036tol	-5.10	-5.217	C_7H_8
pyridine	0045eth	-5.08	-3.603	C_2H_6O
pyridine	0062dio	-5.14	-5.187	$C_4H_8O_2$
pyridine	0076but	-4.61	-3.960	C_4H_8O
pyridine	0116pyr	-5.47	-5.685	C_5H_5N

pyridine	0506nit	-5.11	-6.861	CH_3NO_2
cyclohexanone	0008noc	-4.57	-4.901	C_8H_{18}
cyclohexanone	0036tol	-5.05	-5.560	C_7H_8
cyclohexanone	0045eth	-4.41	-3.857	C_2H_6O
cyclohexanone	0062dio	-4.95	-5.542	$C_4H_8O_2$
cyclohexanone	0076but	-4.42	-4.287	C_4H_8O
cyclohexanone	0086eth	-6.43	-6.075	$C_2H_4O_2$
cyclohexanone	0087pro	-7.18	-6.086	$C_3H_6O_2$
cyclohexanone	0506nit	-5.09	-7.157	CH_3NO_2
cyclohexanone	0523cyc	-6.25	-6.191	$C_6H_{10}O$
cyclohexanone	n017	-9.11	-5.994	H_2O_2
acetophenone	0008noc	-4.24	-4.219	C_8H_{18}
acetophenone	0036tol	-4.90	-5.099	C_7H_8
acetophenone	0045eth	-4.12	-3.608	C_2H_6O
acetophenone	0062dio	-5.03	-5.180	$C_4H_8O_2$
acetophenone	0076but	-4.39	-3.907	C_4H_8O
acetophenone	0084met	-7.59	-7.565	C_8H_8O
acetophenone	0086eth	-6.20	-5.823	$C_2H_4O_2$
acetophenone	0506nit	-4.92	-6.905	CH_3NO_2
acetophenone	n017	-7.30	-5.838	H_2O_2
butanone	0008noc	-4.64	-4.396	C_8H_{18}
butanone	0036tol	-5.06	-5.235	C_7H_8
butanone	0045eth	-4.46	-3.698	C_2H_6O
butanone	0062dio	-5.02	-5.308	$C_4H_8O_2$
butanone	0069met	-1.77	-3.170	CH_2O
butanone	0076but	-4.50	-4.030	C_4H_8O
butanone	0086eth	-6.88	-5.928	$C_2H_4O_2$
butanone	0087pro	-7.05	-5.883	$C_3H_6O_2$
butanone	0088but	-7.34	-6.230	$C_4H_8O_2$
butanone	0089pen	-7.54	-7.106	$C_5H_{10}O_2$
butanone	0090hex	-8.07	-7.919	$C_6H_{12}O_2$
butanone	0506nit	-5.24	-7.007	CH_3NO_2
butanone	0648gbu	-4.47	-7.243	$C_4H_6O_2$
benzonitrile	0008noc	-4.34	-4.595	C_8H_{18}
benzonitrile	0036tol	-4.95	-5.433	C_7H_8
benzonitrile	0045eth	-4.05	-3.876	C_2H_6O
benzonitrile	0062dio	-5.14	-5.552	$C_4H_8O_2$
benzonitrile	0076but	-4.58	-4.239	C_4H_8O
benzonitrile	0129ben	-7.28	-5.378	C_7H_5N
benzonitrile	0506nit	-5.05	-7.227	CH_3NO_2
o-nitrotoluene	0053phe	-7.79	-6.750	C_6H_6O
o-nitrotoluene	0086eth	-4.68	-5.684	$C_2H_4O_2$
o-nitrotoluene	0087pro	-5.30	-5.548	$C_3H_6O_2$
o-nitrotoluene	0088but	-5.76	-5.799	$C_4H_8O_2$
o-nitrotoluene	0135met	-8.04	-8.031	$C_7H_7NO_2$
o-nitrotoluene	0215pbr	-9.57	-9.658	C_6H_5OBr
nitroethane	0008noc	-3.89	-3.662	C_8H_{18}
nitroethane	0036tol	-4.88	-4.783	C_7H_8
nitroethane	0045eth	-3.98	-3.503	C_2H_6O
nitroethane	0062dio	-5.28	-5.015	$C_4H_8O_2$
nitroethane	0076but	-4.73	-3.688	C_4H_8O
nitroethane	0130nit	-5.53	-6.660	$C_2H_5NO_2$
nitroethane	0506nit	-5.35	-6.838	CH_3NO_2
nitrobenzene	0044met	-2.93	-2.522	CH_4O
nitrobenzene	0053phe	-7.86	-6.859	C_6H_6O
nitrobenzene	0055ocr	-8.16	-7.431	C_7H_8O
nitrobenzene	0056mcr	-7.29	-7.618	C_7H_8O
nitrobenzene	0057pcr	-8.13	-7.386	C_7H_8O

nitrobenzene	0086eth	-4.78	-5.805	$C_2H_4O_2$
nitrobenzene	0087pro	-5.38	-5.670	$C_3H_6O_2$
nitrobenzene	0088but	-5.84	-5.923	$C_4H_8O_2$
nitrobenzene	0089pen	-6.47	-6.715	$C_5H_{10}O_2$
nitrobenzene	0090hex	-7.26	-7.445	$C_6H_{12}O_2$
nitrobenzene	0118ani	-7.15	-6.552	C_6H_7N
nitrobenzene	0134nit	-7.94	-8.030	$C_6H_5NO_2$
nitrobenzene	0215pbr	-9.76	-9.768	C_6H_5OBr
nitrobenzene	0506nit	-4.90	-6.871	CH_3NO_2
nitrobenzene	n017	-5.45	-5.803	H_2O_2
acetonitrile	0008noc	-3.57	-4.067	C_8H_{18}
acetonitrile	0036tol	-4.68	-5.098	C_7H_8
acetonitrile	0045eth	-4.43	-3.718	C_2H_6O
acetonitrile	0062dio	-5.33	-5.319	$C_4H_8O_2$
acetonitrile	0076but	-4.73	-3.977	C_4H_8O
acetonitrile	0126eth	-4.85	-2.296	C_2H_3N
acetonitrile	0506nit	-5.62	-7.082	CH_3NO_2
acetonitrile	i003	-80.20	-82.438	CH_6N^+
acetonitrile	i004	-75.70	-76.752	$C_3H_{10}N^+$
acetonitrile	i006	-70.90	-71.396	$C_4H_{12}N^+$
acetonitrile	i010	-67.50	-67.812	$C_4H_{12}N^+$
acetonitrile	i013	-66.10	-68.810	$C_3H_{10}N^+$
acetonitrile	i014	-59.80	-61.477	$C_6H_{16}N^+$
acetonitrile	i015	-57.90	-59.558	$C_9H_{22}N^+$
acetonitrile	i018	-76.80	-75.382	$C_6H_8N^+$
acetonitrile	i021	-74.40	-73.525	$C_7H_{10}N^+$
acetonitrile	i032	-71.00	-70.394	$C_4H_{10}N^+$
acetonitrile	i033	-69.20	-69.222	$C_5H_{12}N^+$
acetonitrile	i036	-66.70	-69.732	$C_5H_6N^+$
acetonitrile	i047	-89.30	-93.392	H_4N^+
acetonitrile	i048	-87.70	-88.233	$H_5N_2^+$
acetonitrile	i050	-92.10	-96.972	CH_5O^+
acetonitrile	i053	-71.00	-68.538	$C_4H_{11}O^+$
acetonitrile	i054	-77.50	-77.831	$C_3H_7O^+$
acetonitrile	i056	-69.20	-70.439	$C_8H_9O^+$
acetonitrile	i095	-74.10	-74.385	$C_4H_{10}NO^+$
acetonitrile	i099	-69.70	-73.415	$C_7H_8NO^+$
acetonitrile	i112	-73.50	-80.376	$C_2H_7OS^+$
acetonitrile	i132	-61.90	-62.805	$C_7H_{10}N^+$
acetonitrile	i150	-75.40	-75.559	$C_8H_9O_2^+$
acetonitrile	i154	-85.90	-92.929	$C_2H_5O_2^+$
acetonitrile	i157	-78.30	-78.655	$C_7H_7O_2^+$
acetonitrile	i158	-78.20	-76.927	$C_7H_{10}N^+$
acetonitrile	i159	-65.40	-64.150	$C_8H_{20}N^+$
acetonitrile	i161	-64.80	-65.552	$C_8H_{20}N^+$
acetonitrile	i163	-76.80	-78.021	$C_2H_8N^+$
acetonitrile	i164	-100.20	-89.314	H_3S^+
acetonitrile	i165	-73.50	-75.182	$C_4H_{12}N^+$
acetonitrile	i167	-70.40	-70.787	$C_7H_9O^+$
acetonitrile	i175	-85.20	-85.674	$C_4H_{11}O^+$
acetonitrile	i176	-75.70	-76.347	$C_4H_{12}N^+$
acetonitrile	i178	-78.10	-84.411	$C_6H_7O^+$
acetonitrile	i179	-82.40	-66.241	$C_4H_{11}O^+$
acetonitrile	i180	-74.70	-73.054	$C_4H_9O^+$
acetonitrile	i181	-73.60	-73.512	$C_2H_6NS^+$
acetonitrile	i184	-57.40	-59.783	$C_{12}H_{28}N^+$
acetonitrile	i059	-58.80	-58.662	$C_2H_3O_2^-$
acetonitrile	i064	-55.40	-54.220	$C_7H_5O_2^-$

acetonitrile	i074	-55.10	-54.561	$C_6H_5O^-$
acetonitrile	i101	-46.70	-43.854	$C_6H_4NO_3^-$
acetonitrile	i102	-45.00	-42.860	$C_6H_4NO_3^-$
acetonitrile	i115	-62.40	-63.793	Cl^-
acetonitrile	i116	-59.30	-58.399	Br^-
acetonitrile	i119	-54.60	-52.273	$C_2H_2O_2Cl^-$
acetonitrile	i120	-51.20	-47.628	$C_2H_1O_2Cl_2^-$
acetonitrile	i189	-54.40	-56.633	$C_{10}H_{11}O_2^-$
acetonitrile	i191	-36.00	-39.678	$C_6H_3N_2O_5^-$
acetonitrile	i194	-53.50	-54.921	$C_7H_4O_2Cl^-$
acetonitrile	i199	-46.50	-46.874	$C_7H_5O_3^-$
acetonitrile	i205	-51.90	-51.522	$C_7H_4NO_4^-$
acetonitrile	i208	-43.80	-42.987	$C_6H_2OCl_3^-$
acetonitrile	i209	-59.70	-56.200	$C_9H_9O_2^-$
acetonitrile	i215	-50.60	-46.902	$C_6H_4OCl^-$
acetonitrile	i220	-48.30	-46.625	$C_7H_4NO_4^-$
acetonitrile	i222	-46.90	-46.293	$C_7H_4OF_3^-$
acetonitrile	i227	-46.50	-44.385	$C_7H_4NO^-$
acetonitrile	i231	-53.80	-54.366	$C_7H_5O_3^-$
acetonitrile	i234	-48.00	-46.316	$C_7H_4NO_4^-$
acetonitrile	i240	-55.40	-53.270	$C_6H_6NO_2S^-$
acetonitrile	i243	-56.70	-57.423	$C_4H_7O_2^-$
acetonitrile	i245	-50.80	-49.164	$C_3H_2NO_2^-$
acetonitrile	i247	-55.60	-59.056	$C_7H_{11}O_2^-$
acetonitrile	i255	-54.70	-53.485	$C_2H_3O_3^-$
acetonitrile	i261	-54.00	-54.490	$CH_3O_3S^-$
acetonitrile	i263	-51.00	-51.640	NO_3^-
acetonitrile	i277	-45.60	-45.654	$C_2O_2F_3^-$
nitromethane	0008noc	-3.15	-3.240	C_8H_{18}
nitromethane	0036tol	-4.52	-4.513	C_7H_8
nitromethane	0045eth	-4.16	-3.372	C_2H_6O
nitromethane	0062dio	-5.46	-4.822	$C_4H_8O_2$
nitromethane	0076but	-4.72	-3.475	C_4H_8O
nitromethane	0506nit	-5.38	-6.714	CH_3NO_2
nitromethane	0648gbu	-5.45	-6.791	$C_4H_6O_2$
dimethyl formamide	0008noc	-3.77	-3.763	C_8H_{18}
dimethyl formamide	0036tol	-4.88	-4.886	C_7H_8
dimethyl formamide	0045eth	-5.23	-3.596	C_2H_6O
dimethyl formamide	0062dio	-5.03	-5.144	$C_4H_8O_2$
dimethyl formamide	0076but	-4.56	-3.797	C_4H_8O
dimethyl formamide	0506nit	-5.66	-6.954	CH_3NO_2
dimethyl formamide	0515dim	-6.47	-7.333	C_3H_7NO
dimethyl acetamide	0008noc	-3.94	-3.936	C_8H_{18}
dimethyl acetamide	0036tol	-4.94	-5.011	C_7H_8
dimethyl acetamide	0045eth	-5.40	-3.672	C_2H_6O
dimethyl acetamide	0062dio	-5.01	-5.252	$C_4H_8O_2$
dimethyl acetamide	0076but	-4.52	-3.905	C_4H_8O
dimethyl acetamide	0506nit	-5.62	-7.035	CH_3NO_2
dimethyl acetamide	0519dim	-6.77	-7.173	C_4H_9NO
tetrahydrothiophene dioxide	0008noc	-2.44	-3.366	C_8H_{18}
tetrahydrothiophene dioxide	0036tol	-4.23	-4.619	C_7H_8
tetrahydrothiophene dioxide	0045eth	-4.30	-3.453	C_2H_6O
tetrahydrothiophene dioxide	0062dio	-4.90	-4.935	$C_4H_8O_2$
tetrahydrothiophene dioxide	0076but	-4.09	-3.577	C_4H_8O
tetrahydrothiophene dioxide	0110but	-4.25	-2.467	$C_4H_{11}N$
tetrahydrothiophene dioxide	0506nit	-5.28	-6.809	CH_3NO_2
dimethyl sulfoxide	0008noc	-2.84	-3.693	C_8H_{18}
dimethyl sulfoxide	0036tol	-4.42	-4.856	C_7H_8

dimethyl sulfoxide	0045eth	-5.25	-3.599	C_2H_6O
dimethyl sulfoxide	0062dio	-4.90	-5.144	$C_4H_8O_2$
dimethyl sulfoxide	0076but	-4.23	-3.785	C_4H_8O
dimethyl sulfoxide	0503dim	-7.63	-14.062	C_2H_6OS
dimethyl sulfoxide	0506nit	-5.66	-6.968	CH_3NO_2
dimethyl sulfoxide	i041	-54.90	-57.498	$C_2H_2N^-$
dimethyl sulfoxide	i043	-59.50	-55.913	CHN_2^-
dimethyl sulfoxide	i044	-52.90	-54.737	$C_6H_6N^-$
dimethyl sulfoxide	i045	-47.70	-51.239	$C_{12}H_{10}N^-$
dimethyl sulfoxide	i046	-54.00	-65.036	CN^-
dimethyl sulfoxide	i059	-59.20	-59.038	$C_2H_3O_2^-$
dimethyl sulfoxide	i064	-55.60	-54.411	$C_7H_5O_2^-$
dimethyl sulfoxide	i065	-67.60	-77.649	CH_3O^-
dimethyl sulfoxide	i066	-64.60	-71.412	$C_2H_5O^-$
dimethyl sulfoxide	i068	-60.00	-66.989	$C_3H_7O^-$
dimethyl sulfoxide	i070	-56.10	-64.119	$C_4H_9O^-$
dimethyl sulfoxide	i074	-54.20	-54.774	$C_6H_5O^-$
dimethyl sulfoxide	i085	-60.40	-61.016	$C_3H_5O^-$
dimethyl sulfoxide	i086	-59.00	-60.912	$C_5H_9O^-$
dimethyl sulfoxide	i089	-77.00	-82.549	HO^-
dimethyl sulfoxide	i102	-45.40	-42.943	$C_6H_4NO_3^-$
dimethyl sulfoxide	i103	-61.30	-55.255	$CH_2NO_2^-$
dimethyl sulfoxide	i104	-47.40	-43.877	$C_6H_5N_2O_2^-$
dimethyl sulfoxide	i105	-58.00	-59.690	$C_2H_4NO^-$
dimethyl sulfoxide	i110	-53.80	-49.200	$C_6H_5S^-$
dimethyl sulfoxide	i113	-55.40	-65.768	$C_2H_5OS^-$
dimethyl sulfoxide	i115	-62.70	-64.330	Cl^-
dimethyl sulfoxide	i116	-57.80	-58.891	Br^-
dimethyl sulfoxide	i120	-49.20	-47.818	$C_2HO_2Cl_2^-$
dimethyl sulfoxide	i121	-56.10	-55.391	$C_2H_2OF_3^-$
dimethyl sulfoxide	i186	-56.70	-55.934	$C_2H_2N_3^-$
dimethyl sulfoxide	i187	-57.20	-56.083	$C_2H_2N_3^-$
dimethyl sulfoxide	i191	-38.00	-39.663	$C_6H_3N_2O_5^-$
dimethyl sulfoxide	i194	-53.60	-55.057	$C_7H_4O_2Cl^-$
dimethyl sulfoxide	i200	-58.70	-50.754	$C_9H_7O^-$
dimethyl sulfoxide	i201	-59.50	-58.117	$C_3H_6NO_2^-$
dimethyl sulfoxide	i204	-51.80	-51.967	$C_{10}H_7O^-$
dimethyl sulfoxide	i205	-52.60	-51.616	$C_7H_4NO_4^-$
dimethyl sulfoxide	i206	-59.90	-57.256	$C_3H_6NO_2^-$
dimethyl sulfoxide	i209	-59.80	-56.322	$C_9H_9O_2^-$
dimethyl sulfoxide	i211	-59.50	-55.445	$C_7H_6NO_2^-$
dimethyl sulfoxide	i212	-51.50	-53.217	$C_5H_5N_2^-$
dimethyl sulfoxide	i218	-56.50	-53.967	$C_7H_5O_3^-$
dimethyl sulfoxide	i219	-56.10	-55.001	$C_8H_7O_2^-$
dimethyl sulfoxide	i225	-51.30	-53.811	$C_5H_5N_2^-$
dimethyl sulfoxide	i226	-52.60	-49.377	$C_7H_4O_2Cl^-$
dimethyl sulfoxide	i234	-48.20	-46.391	$C_7H_4NO_4^-$
dimethyl sulfoxide	i237	-56.40	-57.123	$C_5H_4N_5^-$
dimethyl sulfoxide	i238	-55.60	-55.989	$C_7H_6NO^-$
dimethyl sulfoxide	i239	-54.00	-57.006	$C_8H_8NO^-$
dimethyl sulfoxide	i240	-54.60	-53.378	$C_6H_6NO_2S^-$
dimethyl sulfoxide	i241	-55.40	-50.993	$C_9H_9O^-$
dimethyl sulfoxide	i244	-49.90	-54.241	$C_{12}H_8N^-$
dimethyl sulfoxide	i248	-56.10	-61.260	$C_5H_5^-$
dimethyl sulfoxide	i251	-56.90	-54.832	$C_2H_5O_2S^-$
dimethyl sulfoxide	i252	-54.90	-50.384	$C_2H_4NS^-$
dimethyl sulfoxide	i254	-57.60	-60.260	CH_2NO^-
dimethyl sulfoxide	i257	-56.70	-57.995	$C_3H_3N_2^-$

dimethyl sulfoxide	i258	-46.00	-45.574	$C_3HN_2^-$
dimethyl sulfoxide	i260	-57.50	-54.553	$CH_4NO_2S^-$
dimethyl sulfoxide	i262	-55.70	-52.460	$C_4H_9S^-$
dimethyl sulfoxide	i265	-54.50	-59.667	NO_2^-
dimethyl sulfoxide	i267	-53.90	-55.051	$C_7H_7O^-$
dimethyl sulfoxide	i270	-56.40	-57.823	$C_8H_5^-$
dimethyl sulfoxide	i271	-41.00	-42.771	$C_9H_5N_2^-$
dimethyl sulfoxide	i272	-54.20	-57.838	$C_3H_3N_2^-$
dimethyl sulfoxide	i274	-58.60	-50.344	$C_4H_4NO_2^-$
dimethyl sulfoxide	i275	-54.50	-53.649	CHN_4^-
dimethyl sulfoxide	i276	-49.20	-48.379	$C_2HNOF_3^-$
dimethyl sulfoxide	i277	-45.00	-45.848	$C_2O_2F_3^-$
dimethyl sulfoxide	i279	-58.90	-58.222	$CH_3N_2O^-$
dimethyl sulfoxide	i280	-52.90	-49.499	$C_9H_6NO^-$
dimethyl sulfoxide	i003	-82.40	-82.796	CH_6N^+
dimethyl sulfoxide	i018	-79.80	-75.547	$C_6H_8N^+$
dimethyl sulfoxide	i036	-67.20	-69.916	$C_5H_6N^+$
dimethyl sulfoxide	i047	-93.90	-93.848	H_4N^+
methyl formamide	0008noc	-3.34	-3.617	C_8H_{18}
methyl formamide	0036tol	-4.34	-4.861	C_7H_8
methyl formamide	0045eth	-5.12	-3.662	C_2H_6O
methyl formamide	0062dio	-4.86	-5.223	$C_4H_8O_2$
methyl formamide	0076but	-4.34	-3.827	C_4H_8O
methyl formamide	0506nit	-5.11	-7.066	CH_3NO_2
methyl formamide	0517met	-8.27	-6.649	C_2H_5NO

Table S15. ESE-PM7 solvation free energies in nonpolar solvents in kcal/mol.

**Solvent**	**Solute**	**ΔG^o_{solv}(exp)**[1,2]	**ΔG^o_{solv}(calc)**	**Formula**
pentane	0005npe	-3.35	-3.365	C_5H_{12}
pentane	0044met	-1.29	-1.278	CH_4O
pentane	0045eth	-2.15	-2.197	C_2H_6O
pentane	0047pro	-2.76	-2.627	C_3H_8O
pentane	0049but	-3.77	-3.503	$C_4H_{10}O$
pentane	0052pen	-3.92	-4.211	$C_5H_{12}O$
pentane	0053phe	-5.67	-5.071	C_6H_6O
pentane	0054hex	-4.97	-5.016	$C_6H_{14}O$
pentane	0058hep	-5.62	-5.790	$C_7H_{16}O$
pentane	0078pen	-4.16	-3.665	$C_5H_{10}O$
pentane	0080hex	-4.79	-4.656	$C_6H_{12}O$
pentane	0081dim	-4.43	-4.641	$C_6H_{12}O$
pentane	0082hep	-5.40	-5.422	$C_7H_{14}O$
pentane	0093met	-3.13	-2.737	$C_3H_6O_2$
pentane	0094met	-3.69	-3.249	$C_4H_8O_2$
pentane	0095eth	-3.69	-3.210	$C_4H_8O_2$
pentane	0097pro	-4.21	-3.779	$C_5H_{10}O_2$
pentane	0098met	-4.96	-4.692	$C_6H_{12}O_2$
pentane	0099but	-4.88	-4.660	$C_6H_{12}O_2$
pentane	0100met	-5.67	-5.493	$C_7H_{14}O_2$
pentane	0101pen	-5.62	-5.478	$C_7H_{14}O_2$
pentane	0103eth	-2.18	-2.230	C_2H_7N
pentane	0106pro	-3.13	-2.977	C_3H_9N
pentane	0118ani	-5.15	-6.573	C_6H_7N
pentane	0162tri	-3.26	-4.202	$CHCl_3$
pentane	0179tri	-4.83	-6.132	$CHBr_3$
hexane	0006nhe	-4.00	-3.978	C_6H_{14}
hexane	0008noc	-5.46	-5.357	C_8H_{18}
hexane	0035ben	-3.96	-3.968	C_6H_6

hexane	0036tol	-4.84	-4.540	C_7H_8
hexane	0037eth	-4.99	-5.376	C_8H_{10}
hexane	0038oxy	-5.22	-5.447	C_8H_{10}
hexane	0039mxy	-4.99	-5.319	C_8H_{10}
hexane	0040pxy	-5.01	-5.392	C_8H_{10}
hexane	0044met	-1.49	-1.223	CH_4O
hexane	0045eth	-2.73	-2.107	C_2H_6O
hexane	0047pro	-2.81	-2.487	C_3H_8O
hexane	0049but	-3.77	-3.325	$C_4H_{10}O$
hexane	0052pen	-4.38	-3.985	$C_5H_{12}O$
hexane	0053phe	-5.49	-4.895	C_6H_6O
hexane	0054hex	-5.14	-4.749	$C_6H_{14}O$
hexane	0055ocr	-6.25	-5.544	C_7H_8O
hexane	0057pcr	-5.86	-5.521	C_7H_8O
hexane	0058hep	-5.75	-5.483	$C_7H_{16}O$
hexane	0062dio	-4.08	-3.442	$C_4H_8O_2$
hexane	0074ben	-5.53	-5.499	C_7H_6O
hexane	0075pro	-2.60	-2.356	C_3H_6O
hexane	0076but	-3.48	-3.001	C_4H_8O
hexane	0080hex	-4.68	-4.402	$C_6H_{12}O$
hexane	0081dim	-4.34	-4.396	$C_6H_{12}O$
hexane	0082hep	-5.36	-5.127	$C_7H_{14}O$
hexane	0084met	-6.05	-5.854	C_8H_8O
hexane	0086eth	-2.83	-2.931	$C_2H_4O_2$
hexane	0087pro	-2.98	-3.216	$C_3H_6O_2$
hexane	0093met	-3.12	-2.611	$C_3H_6O_2$
hexane	0094met	-3.65	-3.078	$C_4H_8O_2$
hexane	0095eth	-3.62	-3.043	$C_4H_8O_2$
hexane	0097pro	-4.10	-3.566	$C_5H_{10}O_2$
hexane	0098met	-4.94	-4.433	$C_6H_{12}O_2$
hexane	0099but	-4.86	-4.406	$C_6H_{12}O_2$
hexane	0100met	-5.64	-5.195	$C_7H_{14}O_2$
hexane	0101pen	-5.52	-5.183	$C_7H_{14}O_2$
hexane	0103eth	-2.09	-2.050	C_2H_7N
hexane	0106pro	-3.13	-2.752	C_3H_9N
hexane	0110but	-3.62	-3.605	$C_4H_{11}N$
hexane	0116pyr	-3.81	-3.871	C_5H_5N
hexane	0118ani	-5.43	-6.344	C_6H_7N
hexane	0130nit	-3.19	-4.555	$C_2H_5NO_2$
hexane	0133nit	-4.64	-5.773	$C_4H_9NO_2$
hexane	0134nit	-6.09	-6.514	$C_6H_5NO_2$
hexane	0151phy	-9.18	-6.942	$C_7H_6O_2$
hexane	0157flu	-4.15	-3.144	C_6H_5F
hexane	0162tri	-3.17	-3.973	$CHCl_3$
hexane	0174chl	-5.14	-4.850	C_6H_5Cl
hexane	0176pdi	-5.69	-5.638	$C_6H_4Cl_2$
hexane	0179tri	-4.38	-5.846	$CHBr_3$
hexane	0186bro	-5.66	-5.617	C_6H_5Br
hexane	0215pbr	-6.96	-6.881	C_6H_5OBr
hexane	0220tri	-5.82	-6.140	$C_3H_9O_4P$
hexane	0221tri	-6.78	-7.480	$C_6H_{15}O_4P$
hexane	0222tri	-7.24	-8.877	$C_9H_{21}O_4P$
hexane	0425dbr	-9.67	-9.892	$C_7H_3NOBr_2$
hexane	0506nit	-2.90	-4.345	CH_3NO_2
hexane	n008	-7.77	-8.038	C_7H_7NO
hexane	n011	-6.18	-6.955	C_7H_9N
heptane	0007nhe	-4.65	-4.677	C_7H_{16}
heptane	0035ben	-4.00	-4.052	C_6H_6

heptane	0036tol	-4.78	-4.634	C_7H_8
heptane	0038oxy	-5.52	-5.553	C_8H_{10}
heptane	0039mxy	-5.67	-5.426	C_8H_{10}
heptane	0040pxy	-5.52	-5.497	C_8H_{10}
heptane	0041nap	-7.02	-7.136	$C_{10}H_8$
heptane	0042ant	-10.00	-10.334	$C_{14}H_{10}$
heptane	0044met	-1.29	-1.295	CH_4O
heptane	0045eth	-2.15	-2.193	C_2H_6O
heptane	0047pro	-3.01	-2.579	C_3H_8O
heptane	0049but	-3.66	-3.427	$C_4H_{10}O$
heptane	0052pen	-4.09	-4.093	$C_5H_{12}O$
heptane	0053phe	-5.32	-5.009	C_6H_6O
heptane	0054hex	-4.89	-4.867	$C_6H_{14}O$
heptane	0055ocr	-6.01	-5.667	C_7H_8O
heptane	0056mcr	-5.01	-5.814	C_7H_8O
heptane	0057pcr	-5.77	-5.645	C_7H_8O
heptane	0058hep	-5.60	-5.611	$C_7H_{16}O$
heptane	0068ani	-5.35	-5.268	C_7H_8O
heptane	0074ben	-5.50	-5.631	C_7H_6O
heptane	0075pro	-2.61	-2.447	C_3H_6O
heptane	0076but	-3.36	-3.102	C_4H_8O
heptane	0078pen	-4.07	-3.558	$C_5H_{10}O$
heptane	0080hex	-4.55	-4.519	$C_6H_{12}O$
heptane	0081dim	-4.30	-4.509	$C_6H_{12}O$
heptane	0082hep	-5.22	-5.254	$C_7H_{14}O$
heptane	0084met	-6.14	-5.991	C_8H_8O
heptane	0087pro	-4.06	-3.336	$C_3H_6O_2$
heptane	0088but	-5.05	-3.903	$C_4H_8O_2$
heptane	0089pen	-5.23	-4.781	$C_5H_{10}O_2$
heptane	0090hex	-6.54	-5.613	$C_6H_{12}O_2$
heptane	0093met	-2.97	-2.726	$C_3H_6O_2$
heptane	0094met	-3.63	-3.199	$C_4H_8O_2$
heptane	0095eth	-3.50	-3.169	$C_4H_8O_2$
heptane	0097pro	-4.09	-3.697	$C_5H_{10}O_2$
heptane	0098met	-4.92	-4.570	$C_6H_{12}O_2$
heptane	0099but	-4.83	-4.547	$C_6H_{12}O_2$
heptane	0100met	-5.63	-5.341	$C_7H_{14}O_2$
heptane	0101pen	-5.42	-5.333	$C_7H_{14}O_2$
heptane	0103eth	-2.09	-2.100	C_2H_7N
heptane	0106pro	-3.03	-2.812	C_3H_9N
heptane	0110but	-3.55	-3.675	$C_4H_{11}N$
heptane	0116pyr	-4.28	-3.968	C_5H_5N
heptane	0118ani	-5.38	-6.447	C_6H_7N
heptane	0126eth	-2.06	-1.668	C_2H_3N
heptane	0129ben	-5.33	-4.658	C_7H_5N
heptane	0134nit	-6.14	-6.644	$C_6H_5NO_2$
heptane	0157flu	-4.13	-3.229	C_6H_5F
heptane	0174chl	-5.15	-4.939	C_6H_5Cl
heptane	0175odi	-6.01	-6.010	$C_6H_4Cl_2$
heptane	0176pdi	-5.81	-5.728	$C_6H_4Cl_2$
heptane	0186bro	-5.72	-5.707	C_6H_5Br
heptane	0187dib	-7.55	-7.264	$C_6H_4Br_2$
heptane	0220tri	-5.59	-6.292	$C_3H_9O_4P$
heptane	0221tri	-6.67	-7.665	$C_6H_{15}O_4P$
heptane	0222tri	-7.50	-9.076	$C_9H_{21}O_4P$
heptane	0239oct	-5.68	-5.969	$C_8H_{16}O$
heptane	0245thi	-4.09	-3.303	C_4H_4S
heptane	0414dcl	-9.22	-10.649	$C_{12}H_8Cl_2$

heptane	0519dim	-4.80	-4.449	C_4H_9NO
heptane	n008	-7.26	-8.192	C_7H_7NO
heptane	n009	-6.28	-7.200	C_7H_9N
heptane	n010	-6.35	-7.264	C_7H_9N
heptane	n011	-6.15	-7.068	C_7H_9N
heptane	n186	-5.80	-5.157	C_5H_9NO
heptane	n200	-11.28	-9.434	$C_4H_3N_2O_2F$
heptane	n203	-12.73	-11.294	$C_4H_3N_2O_2Br$
heptane	test4001	-6.27	-6.410	C_6H_5I
isooctane	0005npe	-3.21	-3.065	C_5H_{12}
isooctane	0006nhe	-3.08	-3.939	C_6H_{14}
isooctane	0008noc	-5.44	-5.308	C_8H_{18}
isooctane	0022pro	-1.61	-1.360	C_3H_6
isooctane	0025buta	-2.26	-2.309	C_4H_8
isooctane	0027pen	-2.36	-2.908	C_5H_{10}
isooctane	0035ben	-4.01	-3.990	C_6H_6
isooctane	0036tol	-4.68	-4.559	C_7H_8
isooctane	0039mxy	-5.12	-5.336	C_8H_{10}
isooctane	0045eth	-2.44	-2.174	C_2H_6O
isooctane	0047pro	-3.00	-2.541	C_3H_8O
isooctane	0049but	-3.56	-3.376	$C_4H_{10}O$
isooctane	0052pen	-4.17	-4.024	$C_5H_{12}O$
isooctane	0053phe	-5.30	-4.961	C_6H_6O
isooctane	0054hex	-5.10	-4.784	$C_6H_{14}O$
isooctane	0055ocr	-5.68	-5.606	C_7H_8O
isooctane	0057pcr	-5.59	-5.583	C_7H_8O
isooctane	0062dio	-4.02	-3.529	$C_4H_8O_2$
isooctane	0072but	-3.45	-3.373	C_4H_8O
isooctane	0073pen	-4.24	-4.306	$C_5H_{10}O$
isooctane	0075pro	-2.44	-2.414	C_3H_6O
isooctane	0076but	-3.40	-3.054	C_4H_8O
isooctane	0078pen	-4.14	-3.494	$C_5H_{10}O$
isooctane	0080hex	-4.72	-4.440	$C_6H_{12}O$
isooctane	0110but	-3.57	-3.584	$C_4H_{11}N$
isooctane	0118ani	-5.20	-6.376	C_6H_7N
isooctane	0131nit	-3.94	-5.004	$C_3H_7NO_2$
isooctane	0137ethb	-3.13	-2.803	C_2H_6S
isooctane	0138pro	-3.78	-3.481	C_3H_8S
isooctane	0162tri	-3.06	-3.943	$CHCl_3$
isooctane	0240met	-6.71	-5.768	$C_8H_8O_2$
isooctane	0506nit	-2.82	-4.447	CH_3NO_2
octane	0008noc	-5.28	-5.302	C_8H_{18}
octane	0036tol	-4.82	-4.560	C_7H_8
octane	0044met	-1.29	-1.292	CH_4O
octane	0045eth	-2.15	-2.179	C_2H_6O
octane	0047pro	-2.76	-2.545	C_3H_8O
octane	0049but	-3.69	-3.380	$C_4H_{10}O$
octane	0052pen	-4.10	-4.027	$C_5H_{12}O$
octane	0053phe	-5.47	-4.966	C_6H_6O
octane	0054hex	-4.86	-4.786	$C_6H_{14}O$
octane	0055ocr	-6.16	-5.610	C_7H_8O
octane	0056mcr	-5.19	-5.756	C_7H_8O
octane	0057pcr	-6.19	-5.587	C_7H_8O
octane	0058hep	-5.56	-5.515	$C_7H_{16}O$
octane	0075pro	-2.46	-2.418	C_3H_6O
octane	0076but	-3.24	-3.057	C_4H_8O
octane	0078pen	-3.97	-3.497	$C_5H_{10}O$
octane	0080hex	-4.60	-4.443	$C_6H_{12}O$

octane	0081dim	-4.21	-4.435	$C_6H_{12}O$
octane	0082hep	-5.25	-5.163	$C_7H_{14}O$
octane	0093met	-3.06	-2.704	$C_3H_6O_2$
octane	0094met	-3.57	-3.159	$C_4H_8O_2$
octane	0095eth	-3.48	-3.132	$C_4H_8O_2$
octane	0097pro	-4.09	-3.642	$C_5H_{10}O_2$
octane	0098met	-4.86	-4.497	$C_6H_{12}O_2$
octane	0099but	-4.80	-4.477	$C_6H_{12}O_2$
octane	0100met	-5.53	-5.254	$C_7H_{14}O_2$
octane	0101pen	-5.36	-5.249	$C_7H_{14}O_2$
octane	0103eth	-2.04	-2.036	C_2H_7N
octane	0106pro	-3.00	-2.732	C_3H_9N
octane	0110but	-3.44	-3.582	$C_4H_{11}N$
octane	0111die	-3.42	-3.260	$C_4H_{11}N$
octane	0117met	-4.70	-4.646	$C_5H_6N_2$
octane	0118ani	-4.84	-6.378	C_6H_7N
octane	0131nit	-3.95	-5.009	$C_3H_7NO_2$
octane	0230eth	-5.51	-5.291	$C_6H_8N_2$
octane	n009	-6.06	-7.121	C_7H_9N
octane	n010	-6.15	-7.183	C_7H_9N
octane	n011	-6.00	-6.986	C_7H_9N
nonane	0044met	-1.29	-1.383	CH_4O
nonane	0045eth	-2.15	-2.295	C_2H_6O
nonane	0047pro	-2.76	-2.680	C_3H_8O
nonane	0049but	-3.77	-3.535	$C_4H_{10}O$
nonane	0052pen	-3.92	-4.202	$C_5H_{12}O$
nonane	0053phe	-5.60	-5.134	C_6H_6O
nonane	0054hex	-4.97	-4.983	$C_6H_{14}O$
nonane	0055ocr	-6.20	-5.799	C_7H_8O
nonane	0058hep	-5.62	-5.734	$C_7H_{16}O$
nonane	0076but	-3.20	-3.209	C_4H_8O
nonane	0078pen	-3.97	-3.667	$C_5H_{10}O$
nonane	0080hex	-4.59	-4.635	$C_6H_{12}O$
nonane	0081dim	-4.19	-4.621	$C_6H_{12}O$
nonane	0082hep	-5.24	-5.376	$C_7H_{14}O$
nonane	0093met	-3.02	-2.859	$C_3H_6O_2$
nonane	0094met	-3.50	-3.333	$C_4H_8O_2$
nonane	0095eth	-3.45	-3.310	$C_4H_8O_2$
nonane	0097pro	-4.07	-3.838	$C_5H_{10}O_2$
nonane	0098met	-4.85	-4.711	$C_6H_{12}O_2$
nonane	0099but	-4.69	-4.695	$C_6H_{12}O_2$
nonane	0100met	-5.51	-5.488	$C_7H_{14}O_2$
nonane	0101pen	-5.33	-5.487	$C_7H_{14}O_2$
nonane	0103eth	-1.98	-2.138	C_2H_7N
nonane	0106pro	-2.96	-2.856	C_3H_9N
nonane	0110but	-3.55	-3.726	$C_4H_{11}N$
nonane	0511non	-5.91	-6.285	C_9H_{20}
decane	0008noc	-5.18	-5.262	C_8H_{18}
decane	0035ben	-3.80	-4.006	C_6H_6
decane	0036tol	-4.65	-4.572	C_7H_8
decane	0037eth	-5.25	-5.399	C_8H_{10}
decane	0044met	-1.29	-1.340	CH_4O
decane	0045eth	-2.44	-2.230	C_2H_6O
decane	0047pro	-2.76	-2.585	C_3H_8O
decane	0049but	-3.77	-3.417	$C_4H_{10}O$
decane	0052pen	-3.92	-4.055	$C_5H_{12}O$
decane	0053phe	-5.50	-5.015	C_6H_6O
decane	0054hex	-4.97	-4.811	$C_6H_{14}O$

decane	0057pcr	-6.00	-5.633	C_7H_8O
decane	0058hep	-5.62	-5.537	$C_7H_{16}O$
decane	0062dio	-3.97	-3.601	$C_4H_8O_2$
decane	0075pro	-2.47	-2.461	C_3H_6O
decane	0076but	-3.30	-3.096	C_4H_8O
decane	0078pen	-3.93	-3.528	$C_5H_{10}O$
decane	0080hex	-4.61	-4.470	$C_6H_{12}O$
decane	0081dim	-4.15	-4.462	$C_6H_{12}O$
decane	0082hep	-5.18	-5.187	$C_7H_{14}O$
decane	0093met	-2.98	-2.768	$C_3H_6O_2$
decane	0094met	-3.49	-3.216	$C_4H_8O_2$
decane	0095eth	-3.43	-3.195	$C_4H_8O_2$
decane	0097pro	-4.02	-3.694	$C_5H_{10}O_2$
decane	0098met	-4.77	-4.540	$C_6H_{12}O_2$
decane	0099but	-4.66	-4.526	$C_6H_{12}O_2$
decane	0100met	-5.48	-5.294	$C_7H_{14}O_2$
decane	0101pen	-5.31	-5.294	$C_7H_{14}O_2$
decane	0103eth	-1.92	-2.025	C_2H_7N
decane	0106pro	-2.96	-2.717	C_3H_9N
decane	0110but	-3.55	-3.564	$C_4H_{11}N$
decane	0157flu	-3.48	-3.167	C_6H_5F
decane	0173tri	-3.84	-3.819	C_2HCl_3
decane	0174chl	-4.93	-4.863	C_6H_5Cl
decane	0186bro	-5.43	-5.622	C_6H_5Br
decane	0233ethb	-2.85	-4.595	C_2H_5NO
decane	0506nit	-2.81	-4.532	CH_3NO_2
decane	0512dec	-6.53	-6.795	$C_{10}H_{22}$
decane	n011	-6.05	-7.006	C_7H_9N
undecane	0035ben	-4.05	-3.871	C_6H_6
undecane	0036tol	-4.81	-4.414	C_7H_8
undecane	0037eth	-5.44	-5.213	C_8H_{10}
undecane	0110but	-3.55	-3.405	$C_4H_{11}N$
undecane	0162tri	-3.42	-3.782	$CHCl_3$
undecane	0165tri	-3.82	-4.288	$C_2H_3Cl_3$
undecane	0172Edi	-3.60	-2.742	$C_2H_2Cl_2$
undecane	0173tri	-3.87	-3.665	C_2HCl_3
undecane	0174chl	-5.12	-4.702	C_6H_5Cl
undecane	0175odi	-6.11	-5.715	$C_6H_4Cl_2$
undecane	0179tri	-4.84	-5.583	$CHBr_3$
undecane	0204tet	-4.63	-4.533	C_2Cl_4
undecane	0520und	-7.22	-7.903	$C_{12}H_{26}$
dodecane	0045eth	-2.06	-2.180	C_2H_6O
dodecane	0047pro	-2.74	-2.511	C_3H_8O
dodecane	0049but	-3.47	-3.324	$C_4H_{10}O$
dodecane	0052pen	-4.09	-3.939	$C_5H_{12}O$
dodecane	0054hex	-4.28	-4.674	$C_6H_{14}O$
dodecane	0058hep	-5.41	-5.380	$C_7H_{16}O$
dodecane	0084met	-6.11	-5.853	C_8H_8O
dodecane	0513dod	-7.83	-7.962	$C_{12}H_{26}$
cyclohexane	0003pro	-2.09	-1.530	C_3H_8
cyclohexane	0004nbu	-2.86	-2.239	C_4H_{10}
cyclohexane	0005npe	-3.50	-2.988	C_5H_{12}
cyclohexane	0008noc	-5.63	-5.194	C_8H_{18}
cyclohexane	0018cyc	-4.43	-4.177	C_6H_{12}
cyclohexane	0035ben	-4.19	-3.994	C_6H_6
cyclohexane	0036tol	-4.90	-4.554	C_7H_8
cyclohexane	0037eth	-4.97	-5.372	C_8H_{10}
cyclohexane	0038oxy	-5.54	-5.455	C_8H_{10}

cyclohexane	0039mxy	-5.52	-5.324	C_8H_{10}
cyclohexane	0041nap	-7.17	-7.071	$C_{10}H_8$
cyclohexane	0044met	-1.29	-1.362	CH_4O
cyclohexane	0045eth	-2.42	-2.249	C_2H_6O
cyclohexane	0047pro	-2.73	-2.593	C_3H_8O
cyclohexane	0048pro	-2.37	-2.234	C_3H_8O
cyclohexane	0049but	-3.52	-3.418	$C_4H_{10}O$
cyclohexane	0050met	-2.93	-3.014	$C_4H_{10}O$
cyclohexane	0052pen	-3.61	-4.047	$C_5H_{12}O$
cyclohexane	0053phe	-5.57	-5.023	C_6H_6O
cyclohexane	0054hex	-5.31	-4.796	$C_6H_{14}O$
cyclohexane	0055ocr	-6.02	-5.659	C_7H_8O
cyclohexane	0056mcr	-5.20	-5.804	C_7H_8O
cyclohexane	0057pcr	-5.89	-5.635	C_7H_8O
cyclohexane	0058hep	-6.02	-5.515	$C_7H_{16}O$
cyclohexane	0062dio	-4.17	-3.623	$C_4H_8O_2$
cyclohexane	0063die	-3.03	-2.792	$C_4H_{10}O$
cyclohexane	0068ani	-5.38	-5.230	C_7H_8O
cyclohexane	0074ben	-5.71	-5.652	C_7H_6O
cyclohexane	0075pro	-2.67	-2.472	C_3H_6O
cyclohexane	0076but	-3.48	-3.100	C_4H_8O
cyclohexane	0078pen	-4.19	-3.522	$C_5H_{10}O$
cyclohexane	0079pen	-4.30	-3.904	$C_5H_{10}O$
cyclohexane	0080hex	-4.77	-4.458	$C_6H_{12}O$
cyclohexane	0081dim	-4.42	-4.450	$C_6H_{12}O$
cyclohexane	0082hep	-5.47	-5.168	$C_7H_{14}O$
cyclohexane	0084met	-6.29	-5.981	C_8H_8O
cyclohexane	0086eth	-1.73	-3.181	$C_2H_4O_2$
cyclohexane	0087pro	-3.78	-3.430	$C_3H_6O_2$
cyclohexane	0093met	-3.06	-2.791	$C_3H_6O_2$
cyclohexane	0094met	-3.71	-3.229	$C_4H_8O_2$
cyclohexane	0095eth	-3.56	-3.212	$C_4H_8O_2$
cyclohexane	0097pro	-4.36	-3.700	$C_5H_{10}O_2$
cyclohexane	0098met	-5.04	-4.537	$C_6H_{12}O_2$
cyclohexane	0099but	-4.94	-4.526	$C_6H_{12}O_2$
cyclohexane	0100met	-5.75	-5.284	$C_7H_{14}O_2$
cyclohexane	0101pen	-5.71	-5.286	$C_7H_{14}O_2$
cyclohexane	0103eth	-2.04	-1.999	C_2H_7N
cyclohexane	0107tri	-2.63	-2.528	C_3H_9N
cyclohexane	0111die	-3.61	-3.211	$C_4H_{11}N$
cyclohexane	0116pyr	-4.30	-3.947	C_5H_5N
cyclohexane	0118ani	-5.52	-6.388	C_6H_7N
cyclohexane	0119met	-5.05	-4.553	C_6H_7N
cyclohexane	0120met	-5.14	-4.599	C_6H_7N
cyclohexane	0121met	-5.23	-4.575	C_6H_7N
cyclohexane	0122Nme	-6.33	-6.457	C_7H_9N
cyclohexane	0125dim	-5.51	-5.234	C_7H_9N
cyclohexane	0126eth	-1.87	-1.632	C_2H_3N
cyclohexane	0129ben	-5.54	-4.544	C_7H_5N
cyclohexane	0131nit	-4.06	-5.080	$C_3H_7NO_2$
cyclohexane	0134nit	-6.62	-6.639	$C_6H_5NO_2$
cyclohexane	0135met	-6.71	-6.897	$C_7H_7NO_2$
cyclohexane	0138pro	-3.12	-3.392	C_3H_8S
cyclohexane	0144thi	-5.66	-6.642	C_7H_8S
cyclohexane	0150mhy	-6.88	-7.059	$C_7H_6O_2$
cyclohexane	0151phy	-7.19	-7.210	$C_7H_6O_2$
cyclohexane	0157flu	-3.59	-3.148	C_6H_5F
cyclohexane	0165tri	-4.08	-4.400	$C_2H_3Cl_3$

cyclohexane	0173tri	-4.29	-3.763	C_2HCl_3
cyclohexane	0174chl	-5.10	-4.839	C_6H_5Cl
cyclohexane	0176pdi	-5.89	-5.573	$C_6H_4Cl_2$
cyclohexane	0186bro	-5.29	-5.594	C_6H_5Br
cyclohexane	0207tri	-1.53	-3.515	$C_2H_3OF_3$
cyclohexane	0215pbr	-7.14	-6.963	C_6H_5OBr
cyclohexane	0217wat	-0.39	-2.370	H_2O
cyclohexane	0220tri	-5.67	-6.306	$C_3H_9O_4P$
cyclohexane	0221tri	-7.60	-7.610	$C_6H_{15}O_4P$
cyclohexane	0222tri	-7.71	-8.897	$C_9H_{21}O_4P$
cyclohexane	0240met	-7.01	-5.830	$C_8H_8O_2$
cyclohexane	0244tet	-4.41	-3.834	$C_5H_{10}O$
cyclohexane	0246eth	-6.00	-5.796	$C_8H_{10}O$
cyclohexane	0421dfl	-1.81	-2.503	CF_2Cl_2
cyclohexane	0422ftc	-2.63	-3.309	$CFCl_3$
cyclohexane	0425dbr	-6.83	-9.841	$C_7H_3NOBr_2$
cyclohexane	0506nit	-2.86	-4.568	CH_3NO_2
cyclohexane	0515dim	-3.82	-4.328	C_3H_7NO
cyclohexane	0579pyy	-3.77	-4.596	C_4H_5N
cyclohexane	0582qui	-7.38	-6.926	C_9H_7N
cyclohexane	n008	-8.72	-8.227	C_7H_7NO
cyclohexane	n009	-6.44	-7.127	C_7H_9N
cyclohexane	n010	-6.47	-7.188	C_7H_9N
cyclohexane	n011	-6.30	-6.989	C_7H_9N
cyclohexane	test4001	-6.26	-6.285	C_6H_5I
perfluorobenzene	0075pro	-3.82	-3.101	C_3H_6O
perfluorobenzene	0078pen	-5.10	-4.402	$C_5H_{10}O$
perfluorobenzene	0080hex	-5.55	-5.467	$C_6H_{12}O$
perfluorobenzene	0081dim	-5.26	-5.426	$C_6H_{12}O$
perfluorobenzene	0082hep	-6.15	-6.304	$C_7H_{14}O$
perfluorobenzene	0093met	-4.23	-3.508	$C_3H_6O_2$
perfluorobenzene	0095eth	-4.56	-4.060	$C_4H_8O_2$
perfluorobenzene	0097pro	-5.06	-4.675	$C_5H_{10}O_2$
perfluorobenzene	0098met	-5.59	-5.632	$C_6H_{12}O_2$
perfluorobenzene	0099but	-5.52	-5.628	$C_6H_{12}O_2$
perfluorobenzene	0100met	-6.21	-6.500	$C_7H_{14}O_2$
perfluorobenzene	0101pen	-6.16	-6.513	$C_7H_{14}O_2$
perfluorobenzene	0110but	-4.13	-4.373	$C_4H_{11}N$
perfluorobenzene	0505per	-4.42	-4.833	C_6F_6
perfluorobenzene	0506nit	-4.30	-5.141	CH_3NO_2
pentadecane	0093met	-2.82	-2.783	$C_3H_6O_2$
pentadecane	0094met	-3.35	-3.212	$C_4H_8O_2$
pentadecane	0095eth	-3.37	-3.197	$C_4H_8O_2$
pentadecane	0097pro	-3.91	-3.676	$C_5H_{10}O_2$
pentadecane	0098met	-4.59	-4.504	$C_6H_{12}O_2$
pentadecane	0099but	-4.49	-4.495	$C_6H_{12}O_2$
pentadecane	0100met	-5.35	-5.245	$C_7H_{14}O_2$
pentadecane	0101pen	-5.18	-5.248	$C_7H_{14}O_2$
pentadecane	0516pen	-9.91	-10.215	$C_{15}H_{32}$
hexadecane	0001met	0.45	0.110	CH_4
hexadecane	0002eth	-0.67	-0.655	C_2H_6
hexadecane	0003pro	-1.43	-1.396	C_3H_8
hexadecane	0004nbu	-2.20	-2.075	C_4H_{10}
hexadecane	0005npe	-2.95	-2.796	C_5H_{12}
hexadecane	0006nhe	-3.64	-3.625	C_6H_{14}
hexadecane	0007nhe	-4.33	-4.205	C_7H_{16}
hexadecane	0008noc	-5.02	-4.908	C_8H_{18}
hexadecane	0009nhe	-10.52	-10.629	$C_{16}H_{34}$

hexadecane	0010met	-1.92	-2.141	C_4H_{10}
hexadecane	0011dim	-2.48	-2.956	C_5H_{12}
hexadecane	0012met	-3.48	-3.603	C_6H_{14}
hexadecane	0013dim	-3.87	-4.331	C_7H_{16}
hexadecane	0014tri	-4.24	-5.222	C_8H_{18}
hexadecane	0016cyc	-1.78	-1.132	C_3H_6
hexadecane	0017cyc	-3.38	-3.181	C_5H_{10}
hexadecane	0018cyc	-4.04	-3.986	C_6H_{12}
hexadecane	0019met	-4.43	-4.468	C_7H_{14}
hexadecane	0021eth	-0.39	-0.406	C_2H_4
hexadecane	0022pro	-1.29	-1.201	C_3H_6
hexadecane	0023str	-2.10	-1.859	C_4H_6
hexadecane	0025buta	-2.03	-2.115	C_4H_8
hexadecane	0027pen	-2.79	-2.668	C_5H_{10}
hexadecane	0029hex	-3.51	-3.307	C_6H_{12}
hexadecane	0030eth	-0.20	-0.427	C_2H_2
hexadecane	0031pro	-1.40	-1.206	C_3H_4
hexadecane	0032but	-2.07	-1.767	C_4H_6
hexadecane	0033pen	-2.74	-2.588	C_5H_8
hexadecane	0034hex	-3.42	-3.142	C_6H_{10}
hexadecane	0035ben	-3.80	-3.839	C_6H_6
hexadecane	0036tol	-4.54	-4.371	C_7H_8
hexadecane	0037eth	-5.15	-5.156	C_8H_{10}
hexadecane	0038oxy	-5.37	-5.243	C_8H_{10}
hexadecane	0039mxy	-5.24	-5.110	C_8H_{10}
hexadecane	0040pxy	-5.24	-5.187	C_8H_{10}
hexadecane	0041nap	-7.29	-6.851	$C_{10}H_8$
hexadecane	0042ant	-10.32	-9.972	$C_{14}H_{10}$
hexadecane	0043chr	-14.10	-12.797	$C_{18}H_{12}$
hexadecane	0044met	-1.32	-1.302	CH_4O
hexadecane	0045eth	-2.03	-2.162	C_2H_6O
hexadecane	0046eth	-2.81	-2.810	$C_2H_6O_2$
hexadecane	0047pro	-2.77	-2.472	C_3H_8O
hexadecane	0048pro	-2.47	-2.111	C_3H_8O
hexadecane	0049but	-3.55	-3.270	$C_4H_{10}O$
hexadecane	0050met	-2.74	-2.866	$C_4H_{10}O$
hexadecane	0051cyc	-4.42	-3.565	$C_5H_{10}O$
hexadecane	0052pen	-4.24	-3.864	$C_5H_{12}O$
hexadecane	0053phe	-5.14	-4.872	C_6H_6O
hexadecane	0054hex	-4.92	-4.583	$C_6H_{14}O$
hexadecane	0055ocr	-5.78	-5.480	C_7H_8O
hexadecane	0056mcr	-5.91	-5.622	C_7H_8O
hexadecane	0057pcr	-5.88	-5.454	C_7H_8O
hexadecane	0058hep	-5.62	-5.273	$C_7H_{16}O$
hexadecane	0059dec	-7.68	-7.409	$C_{10}H_{22}O$
hexadecane	0060dim	-1.49	-1.550	C_2H_6O
hexadecane	0061tet	-3.60	-3.096	C_4H_8O
hexadecane	0062dio	-3.82	-3.495	$C_4H_8O_2$
hexadecane	0063die	-2.81	-2.632	$C_4H_{10}O$
hexadecane	0066dim	-3.63	-3.107	$C_4H_{10}O_2$
hexadecane	0068ani	-5.35	-5.042	C_7H_8O
hexadecane	0069met	-0.99	-1.947	CH_2O
hexadecane	0070eth	-1.68	-2.104	C_2H_4O
hexadecane	0071proa	-2.48	-2.830	C_3H_6O
hexadecane	0072but	-3.10	-3.276	C_4H_8O
hexadecane	0073pen	-3.89	-4.171	$C_5H_{10}O$
hexadecane	0074ben	-5.44	-5.481	C_7H_6O
hexadecane	0075pro	-2.31	-2.360	C_3H_6O

hexadecane	0076but	-3.12	-2.956	C_4H_8O
hexadecane	0077cyc	-4.39	-3.754	C_5H_8O
hexadecane	0078pen	-3.76	-3.348	$C_5H_{10}O$
hexadecane	0079pen	-3.83	-3.735	$C_5H_{10}O$
hexadecane	0080hex	-4.45	-4.255	$C_6H_{12}O$
hexadecane	0081dim	-3.94	-4.254	$C_6H_{12}O$
hexadecane	0082hep	-5.13	-4.934	$C_7H_{14}O$
hexadecane	0083hep	-5.20	-4.708	$C_7H_{14}O$
hexadecane	0084met	-6.14	-5.781	C_8H_8O
hexadecane	0085non	-6.46	-6.235	$C_9H_{18}O$
hexadecane	0086eth	-2.39	-3.102	$C_2H_4O_2$
hexadecane	0087pro	-3.12	-3.320	$C_3H_6O_2$
hexadecane	0088but	-3.86	-3.816	$C_4H_8O_2$
hexadecane	0089pen	-4.61	-4.637	$C_5H_{10}O_2$
hexadecane	0090hex	-5.35	-5.412	$C_6H_{12}O_2$
hexadecane	0091met	-1.99	-2.680	$C_2H_4O_2$
hexadecane	0092ethb	-2.59	-3.104	$C_3H_6O_2$
hexadecane	0093met	-2.67	-2.672	$C_3H_6O_2$
hexadecane	0094met	-2.68	-3.078	$C_4H_8O_2$
hexadecane	0095eth	-3.25	-3.062	$C_4H_8O_2$
hexadecane	0096met	-4.01	-3.627	$C_5H_{10}O_2$
hexadecane	0097pro	-3.93	-3.518	$C_5H_{10}O_2$
hexadecane	0098met	-4.69	-4.323	$C_6H_{12}O_2$
hexadecane	0099but	-4.61	-4.314	$C_6H_{12}O_2$
hexadecane	0100met	-5.43	-5.043	$C_7H_{14}O_2$
hexadecane	0101pen	-5.20	-5.046	$C_7H_{14}O_2$
hexadecane	0102eth	-13.69	-13.923	$C_{20}H_{40}O_2$
hexadecane	0103eth	-2.29	-1.865	C_2H_7N
hexadecane	0104dim	-2.18	-1.823	C_2H_7N
hexadecane	0106pro	-2.92	-2.517	C_3H_9N
hexadecane	0107tri	-2.21	-2.379	C_3H_9N
hexadecane	0111die	-3.27	-3.026	$C_4H_{11}N$
hexadecane	0113pen	-4.28	-3.958	$C_5H_{13}N$
hexadecane	0115dip	-4.57	-4.423	$C_6H_{15}N$
hexadecane	0116pyr	-4.10	-3.799	C_5H_5N
hexadecane	0118ani	-5.44	-6.205	C_6H_7N
hexadecane	0119met	-4.68	-4.376	C_6H_7N
hexadecane	0120met	-4.91	-4.422	C_6H_7N
hexadecane	0121met	-4.89	-4.399	C_6H_7N
hexadecane	0122Nme	-6.19	-6.257	C_7H_9N
hexadecane	0123dim	-5.52	-5.005	C_7H_9N
hexadecane	0124dim	-5.52	-5.052	C_7H_9N
hexadecane	0125dim	-5.27	-5.029	C_7H_9N
hexadecane	0126eth	-2.37	-1.518	C_2H_3N
hexadecane	0127pro	-2.84	-1.960	C_3H_5N
hexadecane	0128butb	-3.48	-2.658	C_4H_7N
hexadecane	0129ben	-5.51	-4.331	C_7H_5N
hexadecane	0130nit	-3.29	-4.631	$C_2H_5NO_2$
hexadecane	0131nit	-3.95	-4.935	$C_3H_7NO_2$
hexadecane	0132nit	-3.47	-4.850	$C_3H_7NO_2$
hexadecane	0133nit	-4.66	-5.731	$C_4H_9NO_2$
hexadecane	0134nit	-6.22	-6.454	$C_6H_5NO_2$
hexadecane	0135met	-6.52	-6.686	$C_7H_7NO_2$
hexadecane	0137ethb	-2.96	-2.555	C_2H_6S
hexadecane	0138pro	-3.66	-3.190	C_3H_8S
hexadecane	0139thi	-5.61	-5.200	C_6H_6S
hexadecane	0140dim	-3.05	-2.721	C_2H_6S
hexadecane	0141dim	-4.84	-4.119	$C_2H_6S_2$

hexadecane	0142die	-4.23	-4.420	$C_4H_{10}S$
hexadecane	0143dip	-5.61	-5.618	$C_6H_{14}S$
hexadecane	0145pro	-2.73	-2.026	C_3H_6O
hexadecane	0155flu	-0.76	-0.491	C_2H_5F
hexadecane	0157flu	-4.03	-2.978	C_6H_5F
hexadecane	0158flu	-4.03	-3.157	$C_6H_{13}F$
hexadecane	0159flu	-5.25	-4.541	$C_8H_{17}F$
hexadecane	0161dic	-2.76	-2.875	CH_2Cl_2
hexadecane	0162tri	-3.38	-3.710	$CHCl_3$
hexadecane	0163chl	-2.29	-2.445	C_2H_5Cl
hexadecane	0165tri	-3.73	-4.208	$C_2H_3Cl_3$
hexadecane	0166tri	-4.49	-4.702	$C_2H_3Cl_3$
hexadecane	0167chla	-2.86	-2.856	C_3H_7Cl
hexadecane	0168chl	-2.69	-3.256	C_3H_7Cl
hexadecane	0170chl	-2.88	-2.934	C_3H_5Cl
hexadecane	0171Zdi	-3.33	-3.122	$C_2H_2Cl_2$
hexadecane	0172Edi	-3.11	-2.675	$C_2H_2Cl_2$
hexadecane	0173tri	-4.08	-3.567	C_2HCl_3
hexadecane	0174chl	-4.99	-4.650	C_6H_5Cl
hexadecane	0175odi	-6.16	-5.640	$C_6H_4Cl_2$
hexadecane	0176pdi	-6.02	-5.347	$C_6H_4Cl_2$
hexadecane	0178dib	-3.94	-3.897	CH_2Br_2
hexadecane	0179tri	-5.16	-5.482	$CHBr_3$
hexadecane	0180bro	-2.89	-2.870	C_2H_5Br
hexadecane	0182bro	-3.57	-3.411	C_3H_7Br
hexadecane	0183bro	-3.26	-3.719	C_3H_7Br
hexadecane	0184bro	-4.24	-4.139	C_4H_9Br
hexadecane	0185bro	-4.93	-4.808	$C_5H_{11}Br$
hexadecane	0186bro	-5.51	-5.395	C_6H_5Br
hexadecane	0201bro	-2.97	-4.206	C_2HF_3ClBr
hexadecane	0203bro	-1.87	-3.543	C_2HF_4Br
hexadecane	0204tet	-4.88	-4.398	C_2Cl_4
hexadecane	0206tri	-2.89	-4.363	$C_2F_3Cl_3$
hexadecane	0207tri	-1.67	-3.403	$C_2H_3OF_3$
hexadecane	0210dic	-3.90	-4.459	$C_3H_4OF_2Cl_2$
hexadecane	0214tri	-1.91	-3.168	$C_4H_5OF_3$
hexadecane	0216amm	-0.93	-1.214	H_3N
hexadecane	0217wat	-0.35	-2.357	H_2O
hexadecane	0219hyd	-0.72	-0.499	H_2S
hexadecane	0223die	-5.74	-6.041	$C_4H_{10}S_2$
hexadecane	0233ethb	-3.33	-4.522	C_2H_5NO
hexadecane	0236oct	-6.30	-6.000	$C_8H_{18}O$
hexadecane	0237oct	-5.98	-6.202	$C_8H_{16}O$
hexadecane	0239oct	-5.81	-5.590	$C_8H_{16}O$
hexadecane	0240met	-6.31	-5.613	$C_8H_8O_2$
hexadecane	0242dii	-4.02	-3.974	$C_6H_{14}O$
hexadecane	0244tet	-4.08	-3.678	$C_5H_{10}O$
hexadecane	0245thi	-4.01	-2.997	C_4H_4S
hexadecane	0246eth	-5.64	-5.577	$C_8H_{10}O$
hexadecane	0400hyd	1.64	-2.562	H_2
hexadecane	0417brp	-3.42	-3.373	C_3H_5Br
hexadecane	0418bri	-4.04	-4.058	C_4H_9Br
hexadecane	0419brt	-6.36	-6.674	C_7H_7Br
hexadecane	0420pbr	-6.19	-6.030	C_7H_7Br
hexadecane	0423brt	-4.46	-4.806	CCl_3Br
hexadecane	0431pho	-5.43	-5.260	$C_3H_9O_3P$
hexadecane	0471dim	-2.58	-5.355	C_7H_9N
hexadecane	0506nit	-2.58	-4.487	CH_3NO_2

hexadecane	0571dim	-2.52	-5.024	C_7H_9N
hexadecane	0574eth	-2.45	-5.071	C_7H_9N
hexadecane	0939tet	-2.92	-1.767	$C_4H_{12}Si$
hexadecane	n007	-6.37	-6.718	CH_4N_2O
hexadecane	n009	-6.08	-6.922	C_7H_9N
hexadecane	n011	-6.04	-6.780	C_7H_9N
hexadecane	n127	-2.91	-4.663	CH_3NO
hexadecane	test4001	-6.25	-6.075	C_6H_5I
hexadecane	test4002	-5.26	-4.818	CH_2I_2
hexadecane	test4003	-2.88	-2.738	CH_3I
hexadecane	test4004	-3.51	-3.788	C_2H_5I
hexadecane	test4005	-4.10	-4.128	C_3H_5I
hexadecane	test4006	-4.27	-4.368	C_3H_7I
hexadecane	test4007	-4.95	-5.079	C_4H_9I
hexadecane	test4008	-5.63	-5.887	$C_5H_{11}I$
decalin	0036tol	-4.37	-4.582	C_7H_8
decalin	0053phe	-5.38	-5.182	C_6H_6O
decalin	0056mcr	-5.11	-5.950	C_7H_8O
decalin	0057pcr	-5.68	-5.779	C_7H_8O
decalin	0068ani	-5.00	-5.332	C_7H_8O
decalin	0084met	-6.23	-6.141	C_8H_8O
decalin	0086eth	-4.49	-3.474	$C_2H_4O_2$
decalin	0087pro	-4.42	-3.685	$C_3H_6O_2$
decalin	0093met	-2.90	-3.006	$C_3H_6O_2$
decalin	0094met	-3.50	-3.413	$C_4H_8O_2$
decalin	0095eth	-3.47	-3.415	$C_4H_8O_2$
decalin	0097pro	-4.05	-3.867	$C_5H_{10}O_2$
decalin	0098met	-4.83	-4.670	$C_6H_{12}O_2$
decalin	0099but	-4.71	-4.678	$C_6H_{12}O_2$
decalin	0100met	-5.51	-5.403	$C_7H_{14}O_2$
decalin	0101pen	-5.44	-5.421	$C_7H_{14}O_2$
decalin	0110but	-3.72	-3.444	$C_4H_{11}N$
decalin	0118ani	-5.78	-6.452	C_6H_7N
decalin	0122Nme	-6.41	-6.546	C_7H_9N
decalin	0129ben	-5.86	-4.544	C_7H_5N
decalin	0134nit	-6.36	-6.796	$C_6H_5NO_2$
decalin	0144thi	-5.54	-6.627	C_7H_8S
decalin	0157flu	-3.44	-3.163	C_6H_5F
decalin	0174chl	-4.61	-4.839	C_6H_5Cl
decalin	0186bro	-5.25	-5.580	C_6H_5Br
decalin	0240met	-6.76	-5.999	$C_8H_8O_2$
decalin	test4001	-5.96	-6.261	C_6H_5I
carbon tetrachloride	0008noc	-5.39	-5.685	C_8H_{18}
carbon tetrachloride	0024met	-2.63	-2.440	C_4H_8
carbon tetrachloride	0025buta	-2.48	-2.580	C_4H_8
carbon tetrachloride	0028Epe	-3.46	-3.167	C_5H_{10}
carbon tetrachloride	0035ben	-4.50	-4.461	C_6H_6
carbon tetrachloride	0036tol	-5.12	-5.075	C_7H_8
carbon tetrachloride	0037eth	-5.67	-5.954	C_8H_{10}
carbon tetrachloride	0038oxy	-6.07	-6.046	C_8H_{10}
carbon tetrachloride	0039mxy	-5.71	-5.917	C_8H_{10}
carbon tetrachloride	0041nap	-7.55	-7.778	$C_{10}H_8$
carbon tetrachloride	0044met	-2.25	-1.770	CH_4O
carbon tetrachloride	0045eth	-2.96	-2.737	C_2H_6O
carbon tetrachloride	0047pro	-3.64	-3.108	C_3H_8O
carbon tetrachloride	0048pro	-3.15	-2.729	C_3H_8O
carbon tetrachloride	0049but	-4.20	-3.987	$C_4H_{10}O$
carbon tetrachloride	0050met	-3.40	-3.576	$C_4H_{10}O$

carbon tetrachloride	0052pen	-4.73	-4.652	$C_5H_{12}O$
carbon tetrachloride	0053phe	-6.14	-5.664	C_6H_6O
carbon tetrachloride	0054hex	-5.04	-5.457	$C_6H_{14}O$
carbon tetrachloride	0055ocr	-6.51	-6.351	C_7H_8O
carbon tetrachloride	0057pcr	-6.32	-6.330	C_7H_8O
carbon tetrachloride	0058hep	-6.49	-6.227	$C_7H_{16}O$
carbon tetrachloride	0062dio	-4.97	-4.292	$C_4H_8O_2$
carbon tetrachloride	0068ani	-5.49	-5.880	C_7H_8O
carbon tetrachloride	0074ben	-6.11	-6.389	C_7H_6O
carbon tetrachloride	0075pro	-3.35	-2.983	C_3H_6O
carbon tetrachloride	0076but	-4.09	-3.662	C_4H_8O
carbon tetrachloride	0077cyc	-5.26	-4.465	C_5H_8O
carbon tetrachloride	0078pen	-4.81	-4.118	$C_5H_{10}O$
carbon tetrachloride	0080hex	-5.47	-5.111	$C_6H_{12}O$
carbon tetrachloride	0082hep	-6.12	-5.873	$C_7H_{14}O$
carbon tetrachloride	0084met	-7.10	-6.750	C_8H_8O
carbon tetrachloride	0086eth	-3.64	-3.837	$C_2H_4O_2$
carbon tetrachloride	0087pro	-4.09	-4.110	$C_3H_6O_2$
carbon tetrachloride	0088but	-4.81	-4.673	$C_4H_8O_2$
carbon tetrachloride	0090hex	-6.99	-6.438	$C_6H_{12}O_2$
carbon tetrachloride	0093met	-3.82	-3.434	$C_3H_6O_2$
carbon tetrachloride	0094met	-4.43	-3.908	$C_4H_8O_2$
carbon tetrachloride	0095eth	-4.40	-3.915	$C_4H_8O_2$
carbon tetrachloride	0097pro	-5.03	-4.433	$C_5H_{10}O_2$
carbon tetrachloride	0098met	-5.71	-5.300	$C_6H_{12}O_2$
carbon tetrachloride	0099but	-5.59	-5.314	$C_6H_{12}O_2$
carbon tetrachloride	0100met	-6.39	-6.098	$C_7H_{14}O_2$
carbon tetrachloride	0101pen	-6.35	-6.124	$C_7H_{14}O_2$
carbon tetrachloride	0103eth	-2.77	-2.275	C_2H_7N
carbon tetrachloride	0104dim	-2.75	-2.248	C_2H_7N
carbon tetrachloride	0106pro	-3.59	-3.012	C_3H_9N
carbon tetrachloride	0107tri	-3.09	-2.887	C_3H_9N
carbon tetrachloride	0110but	-5.35	-3.909	$C_4H_{11}N$
carbon tetrachloride	0111die	-4.12	-3.600	$C_4H_{11}N$
carbon tetrachloride	0116pyr	-5.01	-4.487	C_5H_5N
carbon tetrachloride	0118ani	-6.10	-6.966	C_6H_7N
carbon tetrachloride	0122Nme	-6.58	-7.116	C_7H_9N
carbon tetrachloride	0129ben	-6.28	-5.103	C_7H_5N
carbon tetrachloride	0131nit	-4.49	-5.743	$C_3H_7NO_2$
carbon tetrachloride	0134nit	-6.92	-7.366	$C_6H_5NO_2$
carbon tetrachloride	0135met	-7.49	-7.656	$C_7H_7NO_2$
carbon tetrachloride	0144thi	-5.66	-7.240	C_7H_8S
carbon tetrachloride	0151phy	-8.16	-8.136	$C_7H_6O_2$
carbon tetrachloride	0157flu	-3.64	-3.617	C_6H_5F
carbon tetrachloride	0174chl	-5.21	-5.333	C_6H_5Cl
carbon tetrachloride	0176pdi	-6.28	-6.071	$C_6H_4Cl_2$
carbon tetrachloride	0186bro	-5.85	-6.094	C_6H_5Br
carbon tetrachloride	0215pbr	-7.86	-7.641	C_6H_5OBr
carbon tetrachloride	0216amm	-1.06	-1.470	H_3N
carbon tetrachloride	0217wat	-0.85	-2.795	H_2O
carbon tetrachloride	0220tri	-7.24	-7.156	$C_3H_9O_4P$
carbon tetrachloride	0221tri	-7.51	-8.643	$C_6H_{15}O_4P$
carbon tetrachloride	0222tri	-8.60	-10.006	$C_9H_{21}O_4P$
carbon tetrachloride	0228met	-2.53	-1.699	CH_5N
carbon tetrachloride	0240met	-7.19	-6.657	$C_8H_8O_2$
carbon tetrachloride	0506nit	-3.52	-5.182	CH_3NO_2
carbon tetrachloride	0525car	-4.35	-4.455	CCl_4
carbon tetrachloride	n008	-9.13	-9.090	C_7H_7NO

carbon tetrachloride	n009	-7.16	-7.757	C_7H_9N
carbon tetrachloride	n010	-7.23	-7.825	C_7H_9N
carbon tetrachloride	n011	-7.24	-7.616	C_7H_9N
carbon tetrachloride	n017	-3.14	-3.701	H_2O_2
carbon tetrachloride	test4001	-6.50	-6.799	C_6H_5I
isopropyltoluene	0093met	-3.32	-3.464	$C_3H_6O_2$
isopropyltoluene	0094met	-4.14	-3.942	$C_4H_8O_2$
isopropyltoluene	0098met	-5.33	-5.342	$C_6H_{12}O_2$
isopropyltoluene	0100met	-6.06	-6.145	$C_7H_{14}O_2$
isopropyltoluene	0101pen	-6.02	-6.172	$C_7H_{14}O_2$
isopropyltoluene	0110but	-4.22	-3.939	$C_4H_{11}N$
mesitylene	0053phe	-6.80	-5.863	C_6H_6O
mesitylene	0076but	-3.95	-3.841	C_4H_8O
mesitylene	0078pen	-4.80	-4.317	$C_5H_{10}O$
mesitylene	0080hex	-5.34	-5.335	$C_6H_{12}O$
mesitylene	0081dim	-4.77	-5.298	$C_6H_{12}O$
mesitylene	0082hep	-5.99	-6.121	$C_7H_{14}O$
mesitylene	0531mes	-6.40	-6.945	C_9H_{12}
tetrachloroethene	0053phe	-6.10	-5.962	C_6H_6O
tetrachloroethene	0075pro	-3.09	-3.213	C_3H_6O
tetrachloroethene	0093met	-3.63	-3.706	$C_3H_6O_2$
tetrachloroethene	0094met	-4.39	-4.216	$C_4H_8O_2$
tetrachloroethene	0095eth	-4.22	-4.228	$C_4H_8O_2$
tetrachloroethene	0097pro	-4.80	-4.781	$C_5H_{10}O_2$
tetrachloroethene	0098met	-5.41	-5.682	$C_6H_{12}O_2$
tetrachloroethene	0099but	-5.35	-5.702	$C_6H_{12}O_2$
tetrachloroethene	0110but	-4.49	-4.174	$C_4H_{11}N$
tetrachloroethene	0204tet	-5.39	-5.100	C_2Cl_4
benzene	0005npe	-2.99	-3.369	C_5H_{12}
benzene	0006nhe	-3.62	-4.297	C_6H_{14}
benzene	0008noc	-5.35	-5.768	C_8H_{18}
benzene	0018cyc	-4.05	-4.579	C_6H_{12}
benzene	0035ben	-4.55	-4.542	C_6H_6
benzene	0036tol	-5.32	-5.165	C_7H_8
benzene	0044met	-2.58	-1.841	CH_4O
benzene	0045eth	-3.42	-2.822	C_2H_6O
benzene	0047pro	-3.87	-3.198	C_3H_8O
benzene	0048pro	-3.48	-2.814	C_3H_8O
benzene	0049but	-4.45	-4.086	$C_4H_{10}O$
benzene	0050met	-3.70	-3.673	$C_4H_{10}O$
benzene	0052pen	-5.10	-4.756	$C_5H_{12}O$
benzene	0053phe	-7.12	-5.775	C_6H_6O
benzene	0054hex	-6.13	-5.571	$C_6H_{14}O$
benzene	0055ocr	-7.44	-6.471	C_7H_8O
benzene	0056mcr	-6.66	-6.627	C_7H_8O
benzene	0057pcr	-7.35	-6.451	C_7H_8O
benzene	0058hep	-6.85	-6.350	$C_7H_{16}O$
benzene	0062dio	-5.21	-4.409	$C_4H_8O_2$
benzene	0075pro	-3.79	-3.072	C_3H_6O
benzene	0076but	-4.46	-3.760	C_4H_8O
benzene	0078pen	-5.14	-4.221	$C_5H_{10}O$
benzene	0080hex	-5.76	-5.224	$C_6H_{12}O$
benzene	0082hep	-6.36	-5.995	$C_7H_{14}O$
benzene	0086eth	-4.02	-3.952	$C_2H_4O_2$
benzene	0087pro	-4.75	-4.229	$C_3H_6O_2$
benzene	0088but	-5.30	-4.797	$C_4H_8O_2$
benzene	0089pen	-6.01	-5.712	$C_5H_{10}O_2$
benzene	0090hex	-6.94	-6.580	$C_6H_{12}O_2$

benzene	0093met	-4.04	-3.546	$C_3H_6O_2$
benzene	0094met	-4.58	-4.026	$C_4H_8O_2$
benzene	0095eth	-4.53	-4.037	$C_4H_8O_2$
benzene	0097pro	-5.21	-4.560	$C_5H_{10}O_2$
benzene	0098met	-5.83	-5.432	$C_6H_{12}O_2$
benzene	0099but	-5.78	-5.450	$C_6H_{12}O_2$
benzene	0100met	-6.47	-6.239	$C_7H_{14}O_2$
benzene	0101pen	-6.53	-6.269	$C_7H_{14}O_2$
benzene	0103eth	-2.73	-2.322	C_2H_7N
benzene	0104dim	-3.01	-2.299	C_2H_7N
benzene	0106pro	-3.68	-3.068	C_3H_9N
benzene	0107tri	-2.80	-2.948	C_3H_9N
benzene	0111die	-4.02	-3.666	$C_4H_{11}N$
benzene	0115dip	-5.09	-5.242	$C_6H_{15}N$
benzene	0116pyr	-5.28	-4.581	C_5H_5N
benzene	0118ani	-6.88	-7.066	C_6H_7N
benzene	0119met	-5.86	-5.255	C_6H_7N
benzene	0121met	-6.17	-5.280	C_6H_7N
benzene	0122Nme	-6.64	-7.230	C_7H_9N
benzene	0125dim	-6.39	-6.009	C_7H_9N
benzene	0134nit	-7.60	-7.492	$C_6H_5NO_2$
benzene	0150mhy	-9.29	-8.121	$C_7H_6O_2$
benzene	0151phy	-9.73	-8.298	$C_7H_6O_2$
benzene	0215pbr	-8.81	-7.758	C_6H_5OBr
benzene	0216amm	-1.12	-1.500	H_3N
benzene	0217wat	-1.71	-2.870	H_2O
benzene	0220tri	-8.02	-7.303	$C_3H_9O_4P$
benzene	0221tri	-8.58	-8.822	$C_6H_{15}O_4P$
benzene	0222tri	-9.34	-10.197	$C_9H_{21}O_4P$
benzene	0225pipa	-5.03	-5.110	$C_5H_{11}N$
benzene	0228met	-2.66	-1.739	CH_5N
benzene	0229hyd	-4.02	-2.927	H_4N_2
benzene	0236oct	-8.06	-7.173	$C_8H_{18}O$
benzene	0240met	-6.27	-6.801	$C_8H_8O_2$
benzene	0401amia	-12.18	-10.778	$C_9H_{12}N_2O$
benzene	0433pho	-9.09	-10.908	$C_4H_7O_4PCl_2$
benzene	0441pho	-9.21	-12.697	$C_8H_{10}NO_5PS$
benzene	0447pho	-8.58	-14.332	$C_{10}H_{14}NO_5PS$
benzene	0506nit	-4.50	-5.290	CH_3NO_2
benzene	0571dim	-7.64	-5.996	C_7H_9N
benzene	n008	-9.93	-9.240	C_7H_7NO
benzene	n009	-7.37	-7.866	C_7H_9N
benzene	n010	-7.61	-7.934	C_7H_9N
benzene	n011	-7.59	-7.724	C_7H_9N
benzene	n017	-4.77	-3.763	H_2O_2
sec-butylbenzene	0093met	-3.91	-3.545	$C_3H_6O_2$
sec-butylbenzene	0095eth	-4.11	-4.018	$C_4H_8O_2$
sec-butylbenzene	0097pro	-4.62	-4.515	$C_5H_{10}O_2$
sec-butylbenzene	0099but	-5.22	-5.386	$C_6H_{12}O_2$
sec-butylbenzene	0101pen	-5.98	-6.186	$C_7H_{14}O_2$
tert-butylbenzene	0076but	-3.94	-3.655	C_4H_8O
tert-butylbenzene	0078pen	-4.72	-4.082	$C_5H_{10}O$
tert-butylbenzene	0080hex	-5.27	-5.054	$C_6H_{12}O$
tert-butylbenzene	0081dim	-4.79	-5.027	$C_6H_{12}O$
tert-butylbenzene	0082hep	-5.88	-5.794	$C_7H_{14}O$
tert-butylbenzene	0093met	-3.57	-3.482	$C_3H_6O_2$
tert-butylbenzene	0094met	-4.17	-3.927	$C_4H_8O_2$
tert-butylbenzene	0095eth	-4.22	-3.944	$C_4H_8O_2$

tert-butylbenzene	0097pro	-4.72	-4.429	$C_5H_{10}O_2$
tert-butylbenzene	0098met	-5.39	-5.265	$C_6H_{12}O_2$
tert-butylbenzene	0099but	-5.25	-5.289	$C_6H_{12}O_2$
tert-butylbenzene	0100met	-6.13	-6.043	$C_7H_{14}O_2$
tert-butylbenzene	0101pen	-5.92	-6.077	$C_7H_{14}O_2$
tert-butylbenzene	0530tbu	-6.43	-7.777	$C_{10}H_{14}$
butylbenzene	0053phe	-6.76	-5.775	C_6H_6O
butylbenzene	0078pen	-4.74	-4.189	$C_5H_{10}O$
butylbenzene	0080hex	-5.31	-5.175	$C_6H_{12}O$
butylbenzene	0081dim	-4.77	-5.144	$C_6H_{12}O$
butylbenzene	0082hep	-5.93	-5.929	$C_7H_{14}O$
butylbenzene	0094met	-4.19	-4.035	$C_4H_8O_2$
butylbenzene	0098met	-5.37	-5.399	$C_6H_{12}O_2$
butylbenzene	0099but	-5.28	-5.425	$C_6H_{12}O_2$
butylbenzene	0100met	-6.09	-6.190	$C_7H_{14}O_2$
butylbenzene	0529but	-6.86	-7.566	$C_{10}H_{14}$
trimethylbenzene	0076but	-3.97	-3.759	C_4H_8O
trimethylbenzene	0078pen	-4.83	-4.198	$C_5H_{10}O$
trimethylbenzene	0080hex	-5.39	-5.185	$C_6H_{12}O$
trimethylbenzene	0081dim	-4.80	-5.153	$C_6H_{12}O$
trimethylbenzene	0082hep	-6.01	-5.940	$C_7H_{14}O$
trimethylbenzene	0093met	-3.58	-3.589	$C_3H_6O_2$
trimethylbenzene	0094met	-4.14	-4.046	$C_4H_8O_2$
trimethylbenzene	0098met	-5.41	-5.411	$C_6H_{12}O_2$
trimethylbenzene	0100met	-6.16	-6.203	$C_7H_{14}O_2$
trimethylbenzene	0101pen	-6.09	-6.239	$C_7H_{14}O_2$
trimethylbenzene	0532tri	-6.47	-6.913	C_9H_{12}
isopropylbenzene	0045eth	-2.90	-2.862	C_2H_6O
isopropylbenzene	0075pro	-3.32	-3.091	C_3H_6O
isopropylbenzene	0076but	-4.02	-3.761	C_4H_8O
isopropylbenzene	0078pen	-4.84	-4.199	$C_5H_{10}O$
isopropylbenzene	0080hex	-5.39	-5.186	$C_6H_{12}O$
isopropylbenzene	0081dim	-4.81	-5.154	$C_6H_{12}O$
isopropylbenzene	0082hep	-5.99	-5.939	$C_7H_{14}O$
isopropylbenzene	0087pro	-4.23	-4.295	$C_3H_6O_2$
isopropylbenzene	0088but	-4.93	-4.839	$C_4H_8O_2$
isopropylbenzene	0094met	-4.19	-4.049	$C_4H_8O_2$
isopropylbenzene	0095eth	-4.22	-4.069	$C_4H_8O_2$
isopropylbenzene	0097pro	-4.78	-4.566	$C_5H_{10}O_2$
isopropylbenzene	0098met	-5.45	-5.413	$C_6H_{12}O_2$
isopropylbenzene	0099but	-5.36	-5.439	$C_6H_{12}O_2$
isopropylbenzene	0100met	-6.19	-6.204	$C_7H_{14}O_2$
isopropylbenzene	0101pen	-6.13	-6.241	$C_7H_{14}O_2$
isopropylbenzene	0110but	-4.06	-3.874	$C_4H_{11}N$
isopropylbenzene	0217wat	-1.41	-2.971	H_2O
isopropylbenzene	0502pro	-6.04	-6.858	C_9H_{12}
toluene	0008noc	-5.38	-6.007	C_8H_{18}
toluene	0036tol	-5.12	-5.405	C_7H_8
toluene	0044met	-2.18	-2.020	CH_4O
toluene	0045eth	-3.33	-3.038	C_2H_6O
toluene	0047pro	-3.71	-3.429	C_3H_8O
toluene	0049but	-4.31	-4.343	$C_4H_{10}O$
toluene	0052pen	-5.17	-5.032	$C_5H_{12}O$
toluene	0053phe	-6.93	-6.063	C_6H_6O
toluene	0054hex	-6.12	-5.874	$C_6H_{14}O$
toluene	0055ocr	-7.43	-6.784	C_7H_8O
toluene	0057pcr	-7.56	-6.765	C_7H_8O
toluene	0058hep	-6.75	-6.678	$C_7H_{16}O$

toluene	0062dio	-4.91	-4.706	$C_4H_8O_2$
toluene	0075pro	-3.59	-3.300	C_3H_6O
toluene	0076but	-4.27	-4.014	C_4H_8O
toluene	0078pen	-5.02	-4.492	$C_5H_{10}O$
toluene	0080hex	-5.60	-5.522	$C_6H_{12}O$
toluene	0081dim	-5.00	-5.479	$C_6H_{12}O$
toluene	0082hep	-6.30	-6.319	$C_7H_{14}O$
toluene	0086eth	-4.00	-4.238	$C_2H_4O_2$
toluene	0087pro	-4.57	-4.528	$C_3H_6O_2$
toluene	0088but	-5.24	-5.114	$C_4H_8O_2$
toluene	0089pen	-5.89	-6.056	$C_5H_{10}O_2$
toluene	0090hex	-6.97	-6.950	$C_6H_{12}O_2$
toluene	0093met	-3.81	-3.830	$C_3H_6O_2$
toluene	0094met	-4.62	-4.329	$C_4H_8O_2$
toluene	0095eth	-4.41	-4.351	$C_4H_8O_2$
toluene	0097pro	-5.00	-4.890	$C_5H_{10}O_2$
toluene	0098met	-5.65	-5.778	$C_6H_{12}O_2$
toluene	0099but	-5.57	-5.807	$C_6H_{12}O_2$
toluene	0100met	-6.38	-6.609	$C_7H_{14}O_2$
toluene	0101pen	-6.41	-6.649	$C_7H_{14}O_2$
toluene	0103eth	-2.67	-2.454	C_2H_7N
toluene	0104dim	-2.68	-2.439	C_2H_7N
toluene	0106pro	-3.51	-3.225	C_3H_9N
toluene	0107tri	-2.71	-3.116	C_3H_9N
toluene	0110but	-4.33	-4.159	$C_4H_{11}N$
toluene	0111die	-3.75	-3.851	$C_4H_{11}N$
toluene	0115dip	-5.24	-5.476	$C_6H_{15}N$
toluene	0116pyr	-5.13	-4.825	C_5H_5N
toluene	0118ani	-6.69	-7.330	C_6H_7N
toluene	0131nit	-5.25	-6.155	$C_3H_7NO_2$
toluene	0215pbr	-8.70	-8.066	C_6H_5OBr
toluene	0216amm	-2.38	-1.583	H_3N
toluene	0217wat	-1.69	-3.051	H_2O
toluene	0228met	-2.65	-1.850	CH_5N
toluene	0240met	-7.96	-7.175	$C_8H_8O_2$
toluene	0506nit	-4.31	-5.558	CH_3NO_2
toluene	0648gbu	-4.70	-6.000	$C_4H_6O_2$
toluene	n011	-7.39	-8.013	C_7H_9N
toluene	n017	-3.14	-3.916	H_2O_2
triethylamine	0008noc	-5.62	-5.294	C_8H_{18}
triethylamine	0036tol	-4.98	-4.904	C_7H_8
triethylamine	0045eth	-4.02	-2.746	C_2H_6O
triethylamine	0062dio	-4.41	-4.286	$C_4H_8O_2$
triethylamine	0076but	-3.86	-3.586	C_4H_8O
triethylamine	0506nit	-3.63	-5.250	CH_3NO_2
triethylamine	0510tri	-4.44	-4.954	$C_6H_{15}N$
xylene	0008noc	-5.29	-5.849	C_8H_{18}
xylene	0036tol	-5.06	-5.302	C_7H_8
xylene	0044met	-1.73	-1.983	CH_4O
xylene	0045eth	-3.42	-2.986	C_2H_6O
xylene	0047pro	-3.57	-3.358	C_3H_8O
xylene	0049but	-4.17	-4.257	$C_4H_{10}O$
xylene	0052pen	-4.72	-4.928	$C_5H_{12}O$
xylene	0053phe	-6.83	-5.974	C_6H_6O
xylene	0054hex	-5.85	-5.753	$C_6H_{14}O$
xylene	0055ocr	-7.25	-6.680	C_7H_8O
xylene	0056mcr	-6.32	-6.838	C_7H_8O
xylene	0057pcr	-7.18	-6.660	C_7H_8O

xylene	0058hep	-6.74	-6.541	$C_7H_{16}O$
xylene	0062dio	-4.86	-4.630	$C_4H_8O_2$
xylene	0075pro	-3.26	-3.234	C_3H_6O
xylene	0076but	-4.23	-3.931	C_4H_8O
xylene	0078pen	-4.87	-4.393	$C_5H_{10}O$
xylene	0080hex	-5.49	-5.406	$C_6H_{12}O$
xylene	0081dim	-4.91	-5.367	$C_6H_{12}O$
xylene	0082hep	-6.15	-6.186	$C_7H_{14}O$
xylene	0086eth	-4.08	-4.188	$C_2H_4O_2$
xylene	0087pro	-4.72	-4.461	$C_3H_6O_2$
xylene	0088but	-5.30	-5.030	$C_4H_8O_2$
xylene	0089pen	-5.71	-5.954	$C_5H_{10}O_2$
xylene	0090hex	-6.67	-6.831	$C_6H_{12}O_2$
xylene	0093met	-3.70	-3.759	$C_3H_6O_2$
xylene	0094met	-4.20	-4.240	$C_4H_8O_2$
xylene	0095eth	-4.26	-4.263	$C_4H_8O_2$
xylene	0097pro	-4.87	-4.784	$C_5H_{10}O_2$
xylene	0098met	-5.61	-5.655	$C_6H_{12}O_2$
xylene	0099but	-5.40	-5.684	$C_6H_{12}O_2$
xylene	0100met	-6.26	-6.471	$C_7H_{14}O_2$
xylene	0101pen	-6.19	-6.511	$C_7H_{14}O_2$
xylene	0103eth	-3.01	-2.380	C_2H_7N
xylene	0104dim	-3.36	-2.368	C_2H_7N
xylene	0106pro	-3.69	-3.133	C_3H_9N
xylene	0107tri	-2.63	-3.033	C_3H_9N
xylene	0111die	-3.93	-3.748	$C_4H_{11}N$
xylene	0113pen	-4.70	-4.780	$C_5H_{13}N$
xylene	0115dip	-5.35	-5.341	$C_6H_{15}N$
xylene	0116pyr	-5.12	-4.739	C_5H_5N
xylene	0118ani	-6.10	-7.226	C_6H_7N
xylene	0215pbr	-8.69	-7.954	C_6H_5OBr
xylene	0217wat	-1.56	-3.039	H_2O
xylene	0225pipa	-5.15	-5.206	$C_5H_{11}N$
xylene	0228met	-3.20	-1.793	CH_5N
xylene	0506nit	-4.20	-5.508	CH_3NO_2
xylene	n011	-7.17	-7.894	C_7H_9N
ethylbenzene	0037eth	-5.67	-6.197	C_8H_{10}
ethylbenzene	0044met	-1.43	-2.013	CH_4O
ethylbenzene	0045eth	-2.49	-3.016	C_2H_6O
ethylbenzene	0047pro	-3.71	-3.379	C_3H_8O
ethylbenzene	0049but	-3.77	-4.275	$C_4H_{10}O$
ethylbenzene	0052pen	-4.72	-4.938	$C_5H_{12}O$
ethylbenzene	0053phe	-6.82	-6.000	C_6H_6O
ethylbenzene	0054hex	-5.68	-5.759	$C_6H_{14}O$
ethylbenzene	0055ocr	-7.25	-6.702	C_7H_8O
ethylbenzene	0058hep	-6.70	-6.544	$C_7H_{16}O$
ethylbenzene	0075pro	-3.41	-3.258	C_3H_6O
ethylbenzene	0076but	-4.12	-3.950	C_4H_8O
ethylbenzene	0078pen	-4.85	-4.405	$C_5H_{10}O$
ethylbenzene	0080hex	-5.49	-5.415	$C_6H_{12}O$
ethylbenzene	0081dim	-4.92	-5.376	$C_6H_{12}O$
ethylbenzene	0082hep	-6.10	-6.191	$C_7H_{14}O$
ethylbenzene	0093met	-3.74	-3.796	$C_3H_6O_2$
ethylbenzene	0094met	-4.29	-4.271	$C_4H_8O_2$
ethylbenzene	0095eth	-4.31	-4.297	$C_4H_8O_2$
ethylbenzene	0097pro	-4.95	-4.811	$C_5H_{10}O_2$
ethylbenzene	0098met	-5.56	-5.674	$C_6H_{12}O_2$
ethylbenzene	0099but	-5.48	-5.707	$C_6H_{12}O_2$

ethylbenzene	0101pen	-6.20	-6.529	$C_7H_{14}O_2$
ethylbenzene	0103eth	-2.59	-2.366	C_2H_7N
ethylbenzene	0106pro	-3.44	-3.115	C_3H_9N
ethylbenzene	0107tri	-2.64	-3.024	C_3H_9N
ethylbenzene	0110but	-4.43	-4.030	$C_4H_{11}N$
ethylbenzene	0215pbr	-8.54	-7.968	C_6H_5OBr
ethylbenzene	0217wat	-1.51	-3.087	H_2O
carbon disulfide	0008noc	-5.68	-5.336	C_8H_{18}
carbon disulfide	0036tol	-5.39	-5.067	C_7H_8
carbon disulfide	0045eth	-2.72	-2.981	C_2H_6O
carbon disulfide	0053phe	-6.27	-5.871	C_6H_6O
carbon disulfide	0062dio	-4.67	-4.600	$C_4H_8O_2$
carbon disulfide	0075pro	-3.14	-3.175	C_3H_6O
carbon disulfide	0076but	-3.85	-3.815	C_4H_8O
carbon disulfide	0086eth	-2.98	-4.278	$C_2H_4O_2$
carbon disulfide	0093met	-3.67	-3.740	$C_3H_6O_2$
carbon disulfide	0095eth	-4.08	-4.194	$C_4H_8O_2$
carbon disulfide	0097pro	-4.63	-4.646	$C_5H_{10}O_2$
carbon disulfide	0131nit	-4.50	-5.994	$C_3H_7NO_2$
carbon disulfide	0506nit	-3.30	-5.574	CH_3NO_2
carbon disulfide	0537car	-3.95	-0.812	C_1S_2
carbon disulfide	n017	-3.14	-3.907	H_2O_2
tetralin	0045eth	-1.54	-2.632	C_2H_6O
tetralin	0075pro	-2.54	-2.726	C_3H_6O
tetralin	0076but	-3.12	-3.243	C_4H_8O
tetralin	0078pen	-3.99	-3.521	$C_5H_{10}O$
tetralin	0080hex	-4.64	-4.365	$C_6H_{12}O$
tetralin	0081dim	-4.19	-4.361	$C_6H_{12}O$
tetralin	0082hep	-5.33	-4.974	$C_7H_{14}O$
tetralin	0217wat	0.07	-3.138	H_2O
tetralin	0534tet	-7.55	-7.023	$C_{10}H_{12}$
dibutyl ether	0008noc	-5.24	-4.977	C_8H_{18}
dibutyl ether	0036tol	-4.87	-5.012	C_7H_8
dibutyl ether	0045eth	-3.51	-3.156	C_2H_6O
dibutyl ether	0062dio	-4.37	-4.814	$C_4H_8O_2$
dibutyl ether	0076but	-3.78	-3.901	C_4H_8O
dibutyl ether	0086eth	-5.21	-4.615	$C_2H_4O_2$
dibutyl ether	0087pro	-6.11	-4.753	$C_3H_6O_2$
dibutyl ether	0111die	-3.80	-3.272	$C_4H_{11}N$
dibutyl ether	0116pyr	-4.65	-4.650	C_5H_5N
dibutyl ether	0117met	-5.12	-5.603	$C_5H_6N_2$
dibutyl ether	0119met	-5.20	-5.264	C_6H_7N
dibutyl ether	0230eth	-5.87	-6.175	$C_6H_8N_2$
dibutyl ether	0501but	-5.76	-5.930	$C_8H_{18}O$
dibutyl ether	0506nit	-3.67	-5.870	CH_3NO_2
dibutyl ether	n017	-5.75	-4.059	H_2O_2
diisopropyl ether	0008noc	-5.38	-5.498	C_8H_{18}
diisopropyl ether	0036tol	-4.91	-5.501	C_7H_8
diisopropyl ether	0041nap	-7.24	-8.498	$C_{10}H_8$
diisopropyl ether	0045eth	-3.90	-3.569	C_2H_6O
diisopropyl ether	0053phe	-8.35	-6.561	C_6H_6O
diisopropyl ether	0062dio	-4.42	-5.386	$C_4H_8O_2$
diisopropyl ether	0069met	-1.04	-3.203	CH_2O
diisopropyl ether	0076but	-3.96	-4.400	C_4H_8O
diisopropyl ether	0086eth	-5.73	-5.150	$C_2H_4O_2$
diisopropyl ether	0087pro	-6.37	-5.323	$C_3H_6O_2$
diisopropyl ether	0088but	-6.85	-5.806	$C_4H_8O_2$
diisopropyl ether	0089pen	-7.59	-6.696	$C_5H_{10}O_2$

diisopropyl ether	0090hex	-8.23	-7.538	$C_6H_{12}O_2$
diisopropyl ether	0107tri	-2.74	-3.059	C_3H_9N
diisopropyl ether	0111die	-3.78	-3.663	$C_4H_{11}N$
diisopropyl ether	0116pyr	-4.88	-5.135	C_5H_5N
diisopropyl ether	0118ani	-6.67	-7.536	C_6H_7N
diisopropyl ether	0151phy	-11.63	-9.709	$C_7H_6O_2$
diisopropyl ether	0217wat	-3.58	-3.841	H_2O
diisopropyl ether	0242dii	-3.97	-5.354	$C_6H_{14}O$
diisopropyl ether	0506nit	-3.90	-6.375	CH_3NO_2
diisopropyl ether	n017	-6.72	-4.349	H_2O_2
hexadecyl iodide	0005npe	-2.59	-2.366	C_5H_{12}
hexadecyl iodide	0006nhe	-3.26	-3.135	C_6H_{14}
hexadecyl iodide	0007nhe	-3.90	-3.663	C_7H_{16}
hexadecyl iodide	0018cyc	-3.66	-3.629	C_6H_{12}
hexadecyl iodide	0019met	-4.07	-4.006	C_7H_{14}
hexadecyl iodide	0035ben	-3.71	-4.194	C_6H_6
hexadecyl iodide	0036tol	-4.41	-4.690	C_7H_8
hexadecyl iodide	0161dic	-2.76	-2.860	CH_2Cl_2
hexadecyl iodide	0162tri	-3.36	-3.337	$CHCl_3$
phenyl ether	0008noc	-4.38	-4.081	C_8H_{18}
phenyl ether	0036tol	-4.86	-4.595	C_7H_8
phenyl ether	0045eth	-3.22	-3.134	C_2H_6O
phenyl ether	0062dio	-4.83	-4.744	$C_4H_8O_2$
phenyl ether	0076but	-4.08	-3.687	C_4H_8O
phenyl ether	0506nit	-4.19	-5.967	CH_3NO_2
fluoroctane	0093met	-3.59	-3.928	$C_3H_6O_2$
fluoroctane	0094met	-4.09	-4.159	$C_4H_8O_2$
fluoroctane	0095eth	-4.16	-4.254	$C_4H_8O_2$
fluoroctane	0097pro	-4.65	-4.505	$C_5H_{10}O_2$
fluoroctane	0098met	-5.33	-5.118	$C_6H_{12}O_2$
fluoroctane	0099but	-5.22	-5.217	$C_6H_{12}O_2$
ethoxybenzene	0008noc	-4.75	-4.284	C_8H_{18}
ethoxybenzene	0036tol	-4.99	-4.845	C_7H_8
ethoxybenzene	0045eth	-3.45	-3.392	C_2H_6O
ethoxybenzene	0062dio	-4.87	-5.095	$C_4H_8O_2$
ethoxybenzene	0076but	-4.28	-3.973	C_4H_8O
ethoxybenzene	0246eth	-6.75	-6.544	$C_8H_{10}O$
ethoxybenzene	0506nit	-4.45	-6.300	CH_3NO_2
anisole	0008noc	-4.62	-4.590	C_8H_{18}
anisole	0036tol	-4.95	-5.072	C_7H_8
anisole	0045eth	-3.59	-3.537	C_2H_6O
anisole	0062dio	-5.06	-5.301	$C_4H_8O_2$
anisole	0068ani	-6.33	-6.254	C_7H_8O
anisole	0076but	-4.43	-4.174	C_4H_8O
anisole	0110but	-4.44	-3.340	$C_4H_{11}N$
anisole	0506nit	-4.69	-6.459	CH_3NO_2
diethyl ether	0008noc	-5.62	-5.450	C_8H_{18}
diethyl ether	0035ben	-4.21	-5.071	C_6H_6
diethyl ether	0036tol	-5.23	-5.686	C_7H_8
diethyl ether	0037eth	-5.45	-6.547	C_8H_{10}
diethyl ether	0038oxy	-5.58	-6.704	C_8H_{10}
diethyl ether	0039mxy	-5.56	-6.562	C_8H_{10}
diethyl ether	0041nap	-7.25	-8.802	$C_{10}H_8$
diethyl ether	0044met	-3.61	-2.839	CH_4O
diethyl ether	0045eth	-4.41	-3.906	C_2H_6O
diethyl ether	0046eth	-6.20	-5.695	$C_2H_6O_2$
diethyl ether	0047pro	-4.90	-4.135	C_3H_8O
diethyl ether	0048pro	-4.44	-3.666	C_3H_8O

diethyl ether	0049but	-5.69	-5.013	$C_4H_{10}O$
diethyl ether	0050met	-4.80	-4.580	$C_4H_{10}O$
diethyl ether	0051cyc	-6.50	-5.266	$C_5H_{10}O$
diethyl ether	0052pen	-6.11	-5.571	$C_5H_{12}O$
diethyl ether	0053phe	-8.75	-6.929	C_6H_6O
diethyl ether	0054hex	-6.82	-6.367	$C_6H_{14}O$
diethyl ether	0056mcr	-7.95	-7.776	C_7H_8O
diethyl ether	0058hep	-7.51	-7.120	$C_7H_{16}O$
diethyl ether	0062dio	-4.67	-5.830	$C_4H_8O_2$
diethyl ether	0063die	-3.39	-3.992	$C_4H_{10}O$
diethyl ether	0068ani	-5.71	-6.924	C_7H_8O
diethyl ether	0070eth	-2.85	-3.808	C_2H_4O
diethyl ether	0071proa	-3.85	-4.615	C_3H_6O
diethyl ether	0074ben	-6.08	-7.867	C_7H_6O
diethyl ether	0076but	-4.09	-4.705	C_4H_8O
diethyl ether	0084met	-6.79	-8.143	C_8H_8O
diethyl ether	0086eth	-6.26	-5.677	$C_2H_4O_2$
diethyl ether	0087pro	-6.75	-5.813	$C_3H_6O_2$
diethyl ether	0088but	-7.32	-6.267	$C_4H_8O_2$
diethyl ether	0089pen	-7.87	-7.154	$C_5H_{10}O_2$
diethyl ether	0090hex	-8.85	-7.992	$C_6H_{12}O_2$
diethyl ether	0103eth	-2.89	-2.333	C_2H_7N
diethyl ether	0104dim	-2.63	-2.435	C_2H_7N
diethyl ether	0106pro	-3.65	-3.038	C_3H_9N
diethyl ether	0107tri	-2.78	-3.141	C_3H_9N
diethyl ether	0110but	-4.24	-3.940	$C_4H_{11}N$
diethyl ether	0111die	-3.83	-3.705	$C_4H_{11}N$
diethyl ether	0115dip	-4.96	-5.214	$C_6H_{15}N$
diethyl ether	0116pyr	-4.81	-5.405	C_5H_5N
diethyl ether	0118ani	-6.51	-7.778	C_6H_7N
diethyl ether	0126eth	-3.59	-2.519	C_2H_3N
diethyl ether	0129ben	-6.36	-5.631	C_7H_5N
diethyl ether	0134nit	-6.85	-8.707	$C_6H_5NO_2$
diethyl ether	0135met	-7.21	-8.937	$C_7H_7NO_2$
diethyl ether	0145pro	-4.87	-3.662	C_3H_6O
diethyl ether	0146met	-5.12	-5.593	$C_3H_8O_2$
diethyl ether	0150mhy	-11.36	-10.048	$C_7H_6O_2$
diethyl ether	0151phy	-12.07	-10.346	$C_7H_6O_2$
diethyl ether	0174chl	-5.42	-5.801	C_6H_5Cl
diethyl ether	0176pdi	-6.18	-6.294	$C_6H_4Cl_2$
diethyl ether	0186bro	-5.99	-6.511	C_6H_5Br
diethyl ether	0216amm	-1.41	-1.543	H_3N
diethyl ether	0217wat	-3.85	-4.256	H_2O
diethyl ether	0219hyd	-0.60	-0.614	H_2S
diethyl ether	0225pipa	-4.82	-5.280	$C_5H_{11}N$
diethyl ether	0228met	-2.32	-1.825	CH_5N
diethyl ether	0229hyd	-3.08	-3.057	H_4N_2
diethyl ether	0233ethb	-6.16	-6.827	C_2H_5NO
diethyl ether	0236oct	-7.25	-7.917	$C_8H_{18}O$
diethyl ether	0506nit	-4.19	-6.854	CH_3NO_2
diethyl ether	0515dim	-5.31	-7.186	C_3H_7NO
diethyl ether	n007	-9.11	-9.126	CH_4N_2O
diethyl ether	n008	-10.60	-10.869	C_7H_7NO
diethyl ether	n017	-7.03	-4.610	H_2O_2
diethyl ether	n127	-5.97	-7.192	CH_3NO
diethyl ether	n191	-15.03	-12.484	$C_4H_4N_2O_2$
diethyl ether	n200	-15.56	-12.421	$C_4H_3N_2O_2F$
diethyl ether	n203	-17.01	-14.178	$C_4H_3N_2O_2Br$

diethyl ether	test4001	-6.64	-7.188	C_6H_5I
diethyl ether	test4003	-3.51	-2.886	CH_3I
bromoform	0044met	-2.79	-2.769	CH_4O
bromoform	0045eth	-3.24	-3.812	C_2H_6O
bromoform	0047pro	-4.03	-4.016	C_3H_8O
bromoform	0049but	-4.72	-4.871	$C_4H_{10}O$
bromoform	0052pen	-5.34	-5.404	$C_5H_{12}O$
bromoform	0053phe	-6.88	-6.780	C_6H_6O
bromoform	0054hex	-6.20	-6.175	$C_6H_{14}O$
bromoform	0055ocr	-7.45	-7.440	C_7H_8O
bromoform	0058hep	-7.10	-6.904	$C_7H_{16}O$
bromoform	0086eth	-4.54	-5.576	$C_2H_4O_2$
bromoform	0179tri	-6.21	-5.300	$CHBr_3$
bromoform	0215pbr	-8.49	-8.548	C_6H_5OBr
iodobenzene	0008noc	-4.72	-4.491	C_8H_{18}
iodobenzene	0036tol	-4.99	-5.063	C_7H_8
iodobenzene	0044met	-2.18	-2.622	CH_4O
iodobenzene	0045eth	-3.18	-3.595	C_2H_6O
iodobenzene	0047pro	-3.52	-3.712	C_3H_8O
iodobenzene	0049but	-4.05	-4.496	$C_4H_{10}O$
iodobenzene	0052pen	-5.02	-4.943	$C_5H_{12}O$
iodobenzene	0053phe	-6.76	-6.398	C_6H_6O
iodobenzene	0054hex	-5.71	-5.638	$C_6H_{14}O$
iodobenzene	0055ocr	-7.14	-6.988	C_7H_8O
iodobenzene	0056mcr	-6.04	-7.143	C_7H_8O
iodobenzene	0057pcr	-7.01	-6.957	C_7H_8O
iodobenzene	0058hep	-6.53	-6.292	$C_7H_{16}O$
iodobenzene	0062dio	-4.94	-5.374	$C_4H_8O_2$
iodobenzene	0076but	-4.22	-4.207	C_4H_8O
iodobenzene	0103eth	-2.73	-1.881	C_2H_7N
iodobenzene	0106pro	-3.54	-2.480	C_3H_9N
iodobenzene	0110but	-4.19	-3.287	$C_4H_{11}N$
iodobenzene	0215pbr	-8.45	-8.053	C_6H_5OBr
iodobenzene	0506nit	-4.10	-6.555	CH_3NO_2
chloroform	0008noc	-5.25	-5.258	C_8H_{18}
chloroform	0018cyc	-4.45	-4.301	C_6H_{12}
chloroform	0035ben	-4.64	-5.038	C_6H_6
chloroform	0036tol	-5.48	-5.637	C_7H_8
chloroform	0037eth	-5.84	-6.475	C_8H_{10}
chloroform	0038oxy	-6.23	-6.641	C_8H_{10}
chloroform	0039mxy	-5.86	-6.497	C_8H_{10}
chloroform	0041nap	-7.89	-8.770	$C_{10}H_8$
chloroform	0044met	-3.32	-2.906	CH_4O
chloroform	0045eth	-3.94	-3.965	C_2H_6O
chloroform	0046eth	-5.98	-5.845	$C_2H_6O_2$
chloroform	0047pro	-4.41	-4.161	C_3H_8O
chloroform	0048pro	-4.28	-3.682	C_3H_8O
chloroform	0049but	-5.28	-5.023	$C_4H_{10}O$
chloroform	0050met	-4.48	-4.588	$C_4H_{10}O$
chloroform	0052pen	-5.90	-5.551	$C_5H_{12}O$
chloroform	0053phe	-7.14	-6.960	C_6H_6O
chloroform	0054hex	-6.67	-6.328	$C_6H_{14}O$
chloroform	0055ocr	-7.55	-7.625	C_7H_8O
chloroform	0056mcr	-6.70	-7.789	C_7H_8O
chloroform	0057pcr	-7.59	-7.600	C_7H_8O
chloroform	0058hep	-7.53	-7.061	$C_7H_{16}O$
chloroform	0062dio	-6.21	-5.900	$C_4H_8O_2$
chloroform	0063die	-4.32	-3.963	$C_4H_{10}O$

chloroform	0068ani	-6.24	-6.912	C_7H_8O
chloroform	0069met	0.12	-3.597	CH_2O
chloroform	0070eth	-3.65	-3.868	C_2H_4O
chloroform	0074ben	-7.09	-7.906	C_7H_6O
chloroform	0075pro	-4.42	-4.091	C_3H_6O
chloroform	0076but	-5.43	-4.720	C_4H_8O
chloroform	0084met	-7.81	-8.157	C_8H_8O
chloroform	0086eth	-4.74	-5.804	$C_2H_4O_2$
chloroform	0087pro	-5.37	-5.909	$C_3H_6O_2$
chloroform	0088but	-5.99	-6.334	$C_4H_8O_2$
chloroform	0089pen	-6.61	-7.200	$C_5H_{10}O_2$
chloroform	0090hex	-7.51	-8.018	$C_6H_{12}O_2$
chloroform	0093met	-4.90	-5.009	$C_3H_6O_2$
chloroform	0094met	-5.48	-5.362	$C_4H_8O_2$
chloroform	0095eth	-5.58	-5.483	$C_4H_8O_2$
chloroform	0097pro	-6.35	-5.847	$C_5H_{10}O_2$
chloroform	0098met	-6.68	-6.573	$C_6H_{12}O_2$
chloroform	0099but	-6.71	-6.699	$C_6H_{12}O_2$
chloroform	0100met	-7.24	-7.340	$C_7H_{14}O_2$
chloroform	0101pen	-7.36	-7.464	$C_7H_{14}O_2$
chloroform	0103eth	-4.02	-2.260	C_2H_7N
chloroform	0104dim	-3.69	-2.377	C_2H_7N
chloroform	0106pro	-4.73	-2.943	C_3H_9N
chloroform	0107tri	-3.90	-3.077	C_3H_9N
chloroform	0111die	-5.23	-3.605	$C_4H_{11}N$
chloroform	0116pyr	-6.45	-5.404	C_5H_5N
chloroform	0117met	-6.99	-6.548	$C_5H_6N_2$
chloroform	0118ani	-7.34	-7.747	C_6H_7N
chloroform	0119met	-6.98	-6.069	C_6H_7N
chloroform	0120met	-7.35	-6.106	C_6H_7N
chloroform	0121met	-7.50	-6.101	C_6H_7N
chloroform	0125dim	-7.74	-6.826	C_7H_9N
chloroform	0126eth	-4.44	-2.501	C_2H_3N
chloroform	0129ben	-7.22	-5.554	C_7H_5N
chloroform	0134nit	-7.78	-8.725	$C_6H_5NO_2$
chloroform	0135met	-8.30	-8.934	$C_7H_7NO_2$
chloroform	0139thi	-7.61	-6.377	C_6H_6S
chloroform	0142die	-6.40	-4.677	$C_4H_{10}S$
chloroform	0144thi	-5.98	-7.654	C_7H_8S
chloroform	0145pro	-4.34	-3.691	C_3H_6O
chloroform	0149mor	-6.72	-5.959	C_4H_9NO
chloroform	0151phy	-10.30	-10.458	$C_7H_6O_2$
chloroform	0157flu	-4.25	-4.073	C_6H_5F
chloroform	0162tri	-4.13	-3.892	$CHCl_3$
chloroform	0174chl	-5.45	-5.734	C_6H_5Cl
chloroform	0176pdi	-6.32	-6.182	$C_6H_4Cl_2$
chloroform	0186bro	-6.07	-6.433	C_6H_5Br
chloroform	0207tri	-3.03	-5.853	$C_2H_3OF_3$
chloroform	0215pbr	-8.59	-8.717	C_6H_5OBr
chloroform	0216amm	-2.41	-1.502	H_3N
chloroform	0217wat	-2.05	-4.387	H_2O
chloroform	0219hyd	-0.51	-0.524	H_2S
chloroform	0220tri	-9.74	-8.844	$C_3H_9O_4P$
chloroform	0221tri	-10.90	-10.314	$C_6H_{15}O_4P$
chloroform	0222tri	-11.11	-11.150	$C_9H_{21}O_4P$
chloroform	0225pipa	-6.37	-5.196	$C_5H_{11}N$
chloroform	0228met	-3.17	-1.776	CH_5N
chloroform	0229hyd	-4.42	-3.010	H_4N_2

chloroform	0230eth	-7.72	-7.123	$C_6H_8N_2$
chloroform	0233ethb	-7.05	-6.915	C_2H_5NO
chloroform	0240met	-7.81	-8.161	$C_8H_8O_2$
chloroform	0242dii	-3.78	-5.532	$C_6H_{14}O$
chloroform	0244tet	-5.84	-5.130	$C_5H_{10}O$
chloroform	0245thi	-5.83	-3.562	C_4H_4S
chloroform	0246eth	-7.16	-7.571	$C_8H_{10}O$
chloroform	0401amia	-13.64	-12.637	$C_9H_{12}N_2O$
chloroform	0402adn	-12.51	-15.490	$C_6H_7N_5$
chloroform	0403thi	-9.71	-11.992	$C_6H_8N_2O_2$
chloroform	0421dfl	-1.55	-1.968	CF_2Cl_2
chloroform	0422ftc	-2.62	-2.617	$CFCl_3$
chloroform	0441pho	-9.51	-14.263	$C_8H_{10}NO_5PS$
chloroform	0506nit	-4.68	-6.965	CH_3NO_2
chloroform	0519dim	-8.38	-7.225	C_4H_9NO
chloroform	0579pyy	-5.50	-5.850	C_4H_5N
chloroform	0582qui	-10.23	-8.929	C_9H_7N
chloroform	n007	-8.56	-9.220	CH_4N_2O
chloroform	n008	-11.06	-10.923	C_7H_7NO
chloroform	n009	-8.23	-8.564	C_7H_9N
chloroform	n011	-8.01	-8.383	C_7H_9N
chloroform	n017	-4.70	-4.666	H_2O_2
chloroform	n186	-9.82	-7.925	C_5H_9NO
chloroform	n191	-14.28	-12.628	$C_4H_4N_2O_2$
chloroform	n200	-14.31	-12.519	$C_4H_3N_2O_2F$
chloroform	n203	-15.03	-14.252	$C_4H_3N_2O_2Br$
chloroform	test4001	-6.60	-7.101	C_6H_5I
dibromoethane	0044met	-2.38	-2.836	CH_4O
dibromoethane	0045eth	-2.69	-3.861	C_2H_6O
dibromoethane	0047pro	-3.82	-4.011	C_3H_8O
dibromoethane	0049but	-4.65	-4.835	$C_4H_{10}O$
dibromoethane	0052pen	-5.44	-5.318	$C_5H_{12}O$
dibromoethane	0053phe	-7.22	-6.770	C_6H_6O
dibromoethane	0054hex	-6.08	-6.055	$C_6H_{14}O$
dibromoethane	0057pcr	-7.52	-7.371	C_7H_8O
dibromoethane	0058hep	-6.64	-6.750	$C_7H_{16}O$
dibromoethane	0215pbr	-9.01	-8.468	C_6H_5OBr
butyl acetate	0041nap	-7.59	-9.137	$C_{10}H_8$
butyl acetate	0044met	-3.04	-3.077	CH_4O
butyl acetate	0045eth	-3.97	-4.179	C_2H_6O
butyl acetate	0046eth	-6.27	-6.132	$C_2H_6O_2$
butyl acetate	0047pro	-4.52	-4.405	C_3H_8O
butyl acetate	0049but	-5.23	-5.301	$C_4H_{10}O$
butyl acetate	0052pen	-5.78	-5.862	$C_5H_{12}O$
butyl acetate	0053phe	-8.96	-7.264	C_6H_6O
butyl acetate	0054hex	-6.62	-6.676	$C_6H_{14}O$
butyl acetate	0055ocr	-8.90	-7.963	C_7H_8O
butyl acetate	0056mcr	-8.44	-8.131	C_7H_8O
butyl acetate	0057pcr	-9.28	-7.940	C_7H_8O
butyl acetate	0058hep	-7.14	-7.444	$C_7H_{16}O$
butyl acetate	0086eth	-6.11	-6.069	$C_2H_4O_2$
butyl acetate	0099but	-5.52	-7.088	$C_6H_{12}O_2$
butyl acetate	0116pyr	-5.31	-5.673	C_5H_5N
butyl acetate	0118ani	-7.30	-8.050	C_6H_7N
butyl acetate	0215pbr	-10.57	-9.061	C_6H_5OBr
butyl acetate	0217wat	-4.13	-4.535	H_2O
butylacetate	0236oct	-8.17	-8.259	$C_8H_{18}O$
butyl acetate	n011	-7.81	-8.717	C_7H_9N

butyl acetate	n017	-6.76	-4.813	H_2O_2
bromooctane	0093met	-3.35	-3.941	$C_3H_6O_2$
bromooctane	0095eth	-3.97	-4.195	$C_4H_8O_2$
bromooctane	0097pro	-4.48	-4.340	$C_5H_{10}O_2$
bromooctane	0099but	-5.11	-4.976	$C_6H_{12}O_2$
bromooctane	0101pen	-5.81	-5.532	$C_7H_{14}O_2$
bromobenzene	0008noc	-5.02	-4.295	C_8H_{18}
bromobenzene	0036tol	-5.13	-5.050	C_7H_8
bromobenzene	0044met	-2.31	-2.745	CH_4O
bromobenzene	0045eth	-3.26	-3.718	C_2H_6O
bromobenzene	0047pro	-3.74	-3.795	C_3H_8O
bromobenzene	0049but	-4.08	-4.563	$C_4H_{10}O$
bromobenzene	0052pen	-5.06	-4.975	$C_5H_{12}O$
bromobenzene	0053phe	-6.87	-6.499	C_6H_6O
bromobenzene	0054hex	-5.92	-5.652	$C_6H_{14}O$
bromobenzene	0055ocr	-7.26	-7.072	C_7H_8O
bromobenzene	0057pcr	-7.12	-7.040	C_7H_8O
bromobenzene	0058hep	-6.68	-6.287	$C_7H_{16}O$
bromobenzene	0062dio	-5.02	-5.527	$C_4H_8O_2$
bromobenzene	0076but	-4.37	-4.280	C_4H_8O
bromobenzene	0093met	-3.87	-4.665	$C_3H_6O_2$
bromobenzene	0095eth	-4.57	-5.039	$C_4H_8O_2$
bromobenzene	0097pro	-4.93	-5.290	$C_5H_{10}O_2$
bromobenzene	0099but	-5.58	-6.042	$C_6H_{12}O_2$
bromobenzene	0101pen	-6.35	-6.709	$C_7H_{14}O_2$
bromobenzene	0103eth	-2.73	-1.814	C_2H_7N
bromobenzene	0106pro	-3.57	-2.390	C_3H_9N
bromobenzene	0110but	-4.22	-3.182	$C_4H_{11}N$
bromobenzene	0118ani	-6.66	-7.165	C_6H_7N
bromobenzene	0186bro	-6.25	-5.785	C_6H_5Br
bromobenzene	0215pbr	-8.49	-8.102	C_6H_5OBr
bromobenzene	0506nit	-4.25	-6.755	CH_3NO_2
bromobenzene	n011	-7.59	-7.714	C_7H_9N
fluorobenzene	0008noc	-4.99	-4.447	C_8H_{18}
fluorobenzene	0036tol	-5.27	-5.161	C_7H_8
fluorobenzene	0045eth	-3.45	-3.787	C_2H_6O
fluorobenzene	0062dio	-5.18	-5.626	$C_4H_8O_2$
fluorobenzene	0076but	-4.60	-4.377	C_4H_8O
fluorobenzene	0157flu	-4.60	-3.627	C_6H_5F
fluorobenzene	0506nit	-4.62	-6.830	CH_3NO_2
chlorobenzene	0008noc	-5.16	-4.342	C_8H_{18}
chlorobenzene	0036tol	-5.18	-5.119	C_7H_8
chlorobenzene	0044met	-2.44	-2.813	CH_4O
chlorobenzene	0045eth	-3.30	-3.796	C_2H_6O
chlorobenzene	0047pro	-3.82	-3.873	C_3H_8O
chlorobenzene	0049but	-4.31	-4.647	$C_4H_{10}O$
chlorobenzene	0052pen	-5.25	-5.060	$C_5H_{12}O$
chlorobenzene	0053phe	-6.96	-6.595	C_6H_6O
chlorobenzene	0054hex	-5.98	-5.742	$C_6H_{14}O$
chlorobenzene	0055ocr	-7.33	-7.174	C_7H_8O
chlorobenzene	0057pcr	-7.23	-7.142	C_7H_8O
chlorobenzene	0058hep	-6.78	-6.382	$C_7H_{16}O$
chlorobenzene	0062dio	-5.08	-5.633	$C_4H_8O_2$
chlorobenzene	0075pro	-3.86	-3.834	C_3H_6O
chlorobenzene	0076but	-4.47	-4.363	C_4H_8O
chlorobenzene	0078pen	-5.29	-4.594	$C_5H_{10}O$
chlorobenzene	0080hex	-5.84	-5.471	$C_6H_{12}O$
chlorobenzene	0081dim	-5.25	-5.431	$C_6H_{12}O$

chlorobenzene	0082hep	-6.46	-6.100	$C_7H_{14}O$
chlorobenzene	0087pro	-4.38	-5.713	$C_3H_6O_2$
chlorobenzene	0093met	-4.00	-4.767	$C_3H_6O_2$
chlorobenzene	0094met	-4.55	-5.012	$C_4H_8O_2$
chlorobenzene	0095eth	-4.63	-5.148	$C_4H_8O_2$
chlorobenzene	0097pro	-5.15	-5.400	$C_5H_{10}O_2$
chlorobenzene	0098met	-5.83	-6.017	$C_6H_{12}O_2$
chlorobenzene	0099but	-5.74	-6.157	$C_6H_{12}O_2$
chlorobenzene	0101pen	-6.49	-6.828	$C_7H_{14}O_2$
chlorobenzene	0103eth	-2.73	-1.845	C_2H_7N
chlorobenzene	0104dim	-2.75	-1.995	C_2H_7N
chlorobenzene	0106pro	-3.59	-2.426	C_3H_9N
chlorobenzene	0107tri	-2.82	-2.632	C_3H_9N
chlorobenzene	0118ani	-6.72	-7.244	C_6H_7N
chlorobenzene	0174chl	-5.66	-5.184	C_6H_5Cl
chlorobenzene	0215pbr	-8.54	-8.199	C_6H_5OBr
chlorobenzene	0216amm	-1.22	-1.257	H_3N
chlorobenzene	0228met	-2.16	-1.462	CH_5N
chlorobenzene	0506nit	-4.32	-6.858	CH_3NO_2
chlorobenzene	n011	-7.54	-7.799	C_7H_9N
chlorohexane	0075pro	-3.45	-3.496	C_3H_6O
chlorohexane	0076but	-4.10	-3.944	C_4H_8O
chlorohexane	0078pen	-4.84	-4.096	$C_5H_{10}O$
chlorohexane	0080hex	-5.42	-4.895	$C_6H_{12}O$
chlorohexane	0081dim	-4.98	-4.873	$C_6H_{12}O$
chlorohexane	0093met	-3.66	-4.395	$C_3H_6O_2$
chlorohexane	0094met	-4.20	-4.558	$C_4H_8O_2$
chlorohexane	0095eth	-4.25	-4.695	$C_4H_8O_2$
chlorohexane	0097pro	-4.84	-4.864	$C_5H_{10}O_2$
chlorohexane	0098met	-5.41	-5.403	$C_6H_{12}O_2$
chlorohexane	0099but	-5.37	-5.543	$C_6H_{12}O_2$
ethyl acetate	0008noc	-4.72	-5.870	C_8H_{18}
ethyl acetate	0036tol	-5.05	-6.237	C_7H_8
ethyl acetate	0044met	-3.37	-3.342	CH_4O
ethyl acetate	0045eth	-4.24	-4.493	C_2H_6O
ethyl acetate	0046eth	-6.82	-6.608	$C_2H_6O_2$
ethyl acetate	0047pro	-4.90	-4.731	C_3H_8O
ethyl acetate	0049but	-5.77	-5.658	$C_4H_{10}O$
ethyl acetate	0052pen	-6.13	-6.236	$C_5H_{12}O$
ethyl acetate	0053phe	-8.70	-7.668	C_6H_6O
ethyl acetate	0054hex	-6.92	-7.082	$C_6H_{14}O$
ethyl acetate	0058hep	-7.56	-7.880	$C_7H_{16}O$
ethyl acetate	0062dio	-5.03	-6.628	$C_4H_8O_2$
ethyl acetate	0086eth	-6.46	-6.499	$C_2H_4O_2$
ethyl acetate	0087pro	-6.95	-6.640	$C_3H_6O_2$
ethyl acetate	0088but	-7.34	-7.113	$C_4H_8O_2$
ethyl acetate	0095eth	-4.46	-6.253	$C_4H_8O_2$
ethyl acetate	0217wat	-4.26	-4.822	H_2O
ethyl acetate	0236oct	-8.41	-8.726	$C_8H_{18}O$
ethyl acetate	0506nit	-5.06	-7.618	CH_3NO_2
ethyl acetate	n011	-7.63	-9.101	C_7H_9N
ethyl acetate	n017	-7.60	-5.040	H_2O_2
ethyl acetate	n191	-15.43	-13.611	$C_4H_4N_2O_2$
ethyl acetate	n200	-16.10	-13.459	$C_4H_3N_2O_2F$
ethyl acetate	n203	-17.93	-15.225	$C_4H_3N_2O_2Br$
acetic acid	0008noc	-3.93	-3.891	C_8H_{18}
acetic acid	0036tol	-4.53	-4.855	C_7H_8
acetic acid	0045eth	-5.25	-3.698	C_2H_6O

acetic acid	0062dio	-5.80	-5.482	$C_4H_8O_2$
acetic acid	0076but	-4.80	-4.174	C_4H_8O
acetic acid	0086eth	-5.30	-5.653	$C_2H_4O_2$
acetic acid	0506nit	-4.88	-6.784	CH_3NO_2
aniline	0008noc	-3.48	-4.159	C_8H_{18}
aniline	0036tol	-4.57	-5.099	C_7H_8
aniline	0045eth	-4.45	-3.899	C_2H_6O
aniline	0062dio	-5.65	-5.761	$C_4H_8O_2$
aniline	0076but	-4.87	-4.420	C_4H_8O
aniline	0086eth	-6.30	-5.911	$C_2H_4O_2$
aniline	0087pro	-6.20	-5.872	$C_3H_6O_2$
aniline	0118ani	-7.61	-7.250	C_6H_7N
aniline	0506nit	-5.11	-7.028	CH_3NO_2
aniline	n017	-7.80	-4.671	H_2O_2
dimethylpyridine	0008noc	-4.88	-4.219	C_8H_{18}
dimethylpyridine	0036tol	-5.03	-5.163	C_7H_8
dimethylpyridine	0045eth	-4.87	-3.959	C_2H_6O
dimethylpyridine	0062dio	-4.90	-5.843	$C_4H_8O_2$
dimethylpyridine	0076but	-4.34	-4.489	C_4H_8O
dimethylpyridine	0125dim	-6.04	-6.362	C_7H_9N
tetrahydrofuran	0008noc	-5.39	-4.274	C_8H_{18}
tetrahydrofuran	0036tol	-5.50	-5.220	C_7H_8
tetrahydrofuran	0045eth	-4.56	-4.011	C_2H_6O
tetrahydrofuran	0061tet	-4.25	-4.525	C_4H_8O
tetrahydrofuran	0062dio	-5.17	-5.914	$C_4H_8O_2$
tetrahydrofuran	0076but	-4.54	-4.549	C_4H_8O
tetrahydrofuran	0506nit	-5.09	-7.168	CH_3NO_2
decanol	0053phe	-8.58	-7.814	C_6H_6O
decanol	0055ocr	-8.58	-8.533	C_7H_8O
decanol	0056mcr	-8.01	-8.708	C_7H_8O
decanol	0057pcr	-8.91	-8.511	C_7H_8O
decanol	0059dec	-9.58	-10.509	$C_{10}H_{22}O$
decanol	0103eth	-3.91	-2.560	C_2H_7N
decanol	0106pro	-4.59	-3.295	C_3H_9N
decanol	0110but	-5.22	-4.240	$C_4H_{11}N$
decanol	0146met	-5.41	-6.678	$C_3H_8O_2$
decanol	0174chl	-4.83	-6.323	C_6H_5Cl
decanol	0215pbr	-10.32	-9.587	C_6H_5OBr
tributyl phosphate	0044met	-4.16	-3.669	CH_4O
tributyl phosphate	0045eth	-4.57	-4.875	C_2H_6O
tributyl phosphate	0047pro	-5.42	-5.118	C_3H_8O
tributyl phosphate	0049but	-6.28	-6.076	$C_4H_{10}O$
tributyl phosphate	0052pen	-6.69	-6.667	$C_5H_{12}O$
tributyl phosphate	0054hex	-7.68	-7.546	$C_6H_{14}O$
tributyl phosphate	0058hep	-7.98	-8.373	$C_7H_{16}O$
tributyl phosphate	0086eth	-7.11	-7.034	$C_2H_4O_2$
tributyl phosphate	0087pro	-7.73	-7.178	$C_3H_6O_2$
tributyl phosphate	0088but	-8.29	-7.662	$C_4H_8O_2$
tributyl phosphate	0089pen	-8.82	-8.627	$C_5H_{10}O_2$
tributyl phosphate	0103eth	-3.29	-2.761	C_2H_7N
tributyl phosphate	0106pro	-3.98	-3.539	C_3H_9N
tributyl phosphate	0118ani	-7.60	-8.812	C_6H_7N
tributyl phosphate	0146met	-6.14	-7.008	$C_3H_8O_2$
tributyl phosphate	0217wat	-4.69	-5.189	H_2O
nonanol	0035ben	-3.82	-5.330	C_6H_6
nonanol	0036tol	-4.34	-5.932	C_7H_8
nonanol	0037eth	-4.61	-6.766	C_8H_{10}
nonanol	0053phe	-8.61	-7.549	C_6H_6O

nonanol	0103eth	-4.02	-2.300	C_2H_7N
nonanol	0106pro	-4.66	-2.971	C_3H_9N
nonanol	0110but	-5.35	-3.862	$C_4H_{11}N$
nonanol	0146met	-5.61	-6.486	$C_3H_8O_2$
nonanol	0215pbr	-10.36	-9.231	C_6H_5OBr
nonanol	0518non	-9.05	-9.151	$C_9H_{20}O$
dichloromethane	0008noc	-5.18	-4.547	C_8H_{18}
dichloromethane	0036tol	-5.53	-5.497	C_7H_8
dichloromethane	0045eth	-3.82	-4.261	C_2H_6O
dichloromethane	0053phe	-7.50	-7.156	C_6H_6O
dichloromethane	0057pcr	-7.71	-7.729	C_7H_8O
dichloromethane	0062dio	-5.33	-6.259	$C_4H_8O_2$
dichloromethane	0139thi	-7.11	-6.109	C_6H_6S
dichloromethane	0161dic	-3.80	-3.213	CH_2Cl_2
dichloromethane	0215pbr	-9.09	-8.745	C_6H_5OBr
dichloromethane	0217wat	-2.63	-4.962	H_2O
dichloromethane	0506nit	-5.05	-7.480	CH_3NO_2

References

(1) Marenich, A. V.; Kelly, C. P.; Thompson, J. D.; Hawkins, G. D.; Chambers, C. C.; Giesen, D. J.; Winget, P.; Cramer, C. J.; Truhlar, D. G. Minnesota Solvation Database – version 2012, University of Minnesota, November 26, 2012. https://comp.chem.umn.edu/mnsol/MNSol-v2012_Manual.pdf retrieved on 05/12/2020.

(2) Marenich, A. V.; Kelly, C. P.; Thompson, J. D.; Hawkins, G. D.; Chambers, C. C.; Giesen, D. J.; Winget, P.; Cramer, C. J.; Truhlar, D. G., Minnesota Solvation Database – version 2012, University of Minnesota, November 26, 2012. https://conservancy.umn.edu/bitstream/handle/11299/213300/MNSolDatabase_v2012.zip retrieved on 17/05/2019.

(3) Kříž, K.; Řezáč, J. Reparametrization of the COSMO solvent model for semiempirical methods PM6 and PM7, *J. Chem. Inf. Model.* **2019**, *59, 229−235*. DOI: 10.1021/acs.jcim.8b00681

(4) Mobley, D. L.; Dill, K. A.; Chodera, J. D."Treating entropy and conformational changes in implicit solvent simulations of small molecules", *J. Phys. Chem. B*, **2008**, *112*, 938−946

(5) Guthrie, J. P. Blind challenge for computational solvation free energies: introduction and overview, *J. Phys. Chem. B*, **2009**, *113*, 4501–4507. DOI: 10.1021/jp806724u

(6) Guthrie, J. P. SAMPL4, a blind challenge for computational solvation free energies: the compounds considered, *J. Comput.-Aided Mol. Des.* **2014**, *28*, 151−168. DOI: 10.1007/s10822-014-9738-y
